# Modeling Pharmacokinetics in Individual Patients Using Therapeutic Drug Monitoring and Artificial Population Quasi-Models: A Study with Piperacillin

**DOI:** 10.3390/pharmaceutics16030358

**Published:** 2024-03-04

**Authors:** Gellért Balázs Karvaly, István Vincze, Michael Noel Neely, István Zátroch, Zsuzsanna Nagy, Ibolya Kocsis, Csaba Kopitkó

**Affiliations:** 1 Department of Laboratory Medicine, Semmelweis University, 1089 Budapest, Hungary; vincze.istvan@pharma.semmelweis-univ.hu (I.V.); kocsis.ibolya@med.semmelweis-univ.hu (I.K.); 2 Laboratory of Applied Pharmacokinetics and Bioinformatics, The Saban Research Institute, University of Southern California, Los Angeles, CA 90027, USA; mneely@chla.usc.edu; 3 Central Department of Anaesthesiology and Intensive Care, Uzsoki Teaching Hospital, 1145 Budapest, Hungary; zatroch.istvan@uzsoki.hu (I.Z.); kopitko.csaba@uzsoki.hu (C.K.); 4 Central Laboratory, Uzsoki Teaching Hospital, 1145 Budapest, Hungary; nagy.zsuzsanna@uzsoki.hu

**Keywords:** therapeutic drug monitoring, piperacillin, tazobactam, nonparametric adaptive grid, Bayesian models, pharmacokinetics, model-informed precision dosing, intensive care

## Abstract

Population pharmacokinetic (pop-PK) models constructed for model-informed precision dosing often have limited utility due to the low number of patients recruited. To augment such models, an approach is presented for generating fully artificial quasi-models which can be employed to make individual estimates of pharmacokinetic parameters. Based on 72 concentrations obtained in 12 patients, one- and two-compartment pop-PK models with or without creatinine clearance as a covariate were generated for piperacillin using the nonparametric adaptive grid algorithm. Thirty quasi-models were subsequently generated for each model type, and nonparametric maximum a posteriori probability Bayesian estimates were established for each patient. A significant difference in performance was found between one- and two-compartment models. Acceptable agreement was found between predicted and observed piperacillin concentrations, and between the estimates of the random-effect pharmacokinetic variables obtained using the so-called support points of the pop-PK models or the quasi-models as priors. The mean squared errors of the predictions made using the quasi-models were similar to, or even considerably lower than those obtained when employing the pop-PK models. Conclusion: fully artificial nonparametric quasi-models can efficiently augment pop-PK models containing few support points, to make individual pharmacokinetic estimates in the clinical setting.

## 1. Introduction

Model-informed precision dosing (MIPD) is an emerging clinical discipline which allows the guidance of individualized drug therapies based on the therapeutic monitoring of drug concentrations and pharmacokinetic modeling. The construction of population (pop-PK) and, subsequently, individual models allows the prediction of each patient’s future exposure to the monitored substance. The clinical implementation of MIPD requires an efficient laboratory assay, suitable computer modeling software, and the efforts of a multidisciplinary team consisting of clinicians, nurses, pharmacists and laboratory analysts [1,2,3]. This discipline has become especially useful in optimizing antibiotic treatments at intensive care units due to the very high vulnerability and, in terms of the pharmacokinetically relevant physiological functions and parameters, variability of critically ill patients [4,5].

The quality of the prediction of individual drug concentrations has a crucial impact on finding the optimal dosage regimen and, eventually, on therapeutic success. Interest in augmenting pharmacokinetic models with the help of machine learning algorithms has recently increased because the construction of models which efficiently represent a broader set of patients has often proved to be an overwhelming task, especially in pediatric populations, as well as in populations diagnosed with rare diseases, or living with special conditions (e.g., oncological and organ-transplant recipients, or patients receiving intensive care) [6,7,8,9]. Augmented pop-PK models are expected to overcome the limitations posed by the availability of a low number of subjects and/or data points, and may therefore facilitate the implementation of MIPD [10].

**Figure 1:**
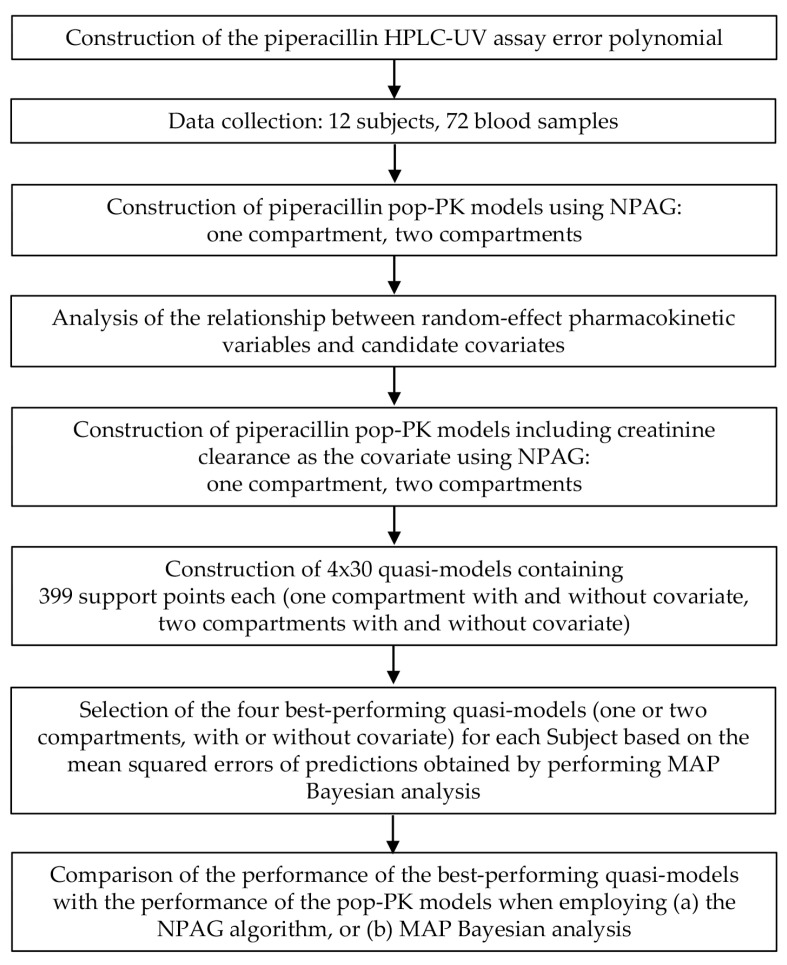
Overview of the workflow for constructing and applying fully artificial quasi-models to make estimates of the individual pharmacokinetic properties of piperacillin. HPLC-UV, high-performance liquid chromatography coupled with ultraviolet light absorbance detection. MAP, maximum a posteriori probability. NPAG, nonparametric adaptive grid modeling. Pop-PK: population pharmacokinetic model.

In this work, a novel approach to constructing a set of fully artificial population pharmacokinetic quasi-models (QM) is put forward. The most suitable of which is then selected for each patient individually to estimate their exposure to the drug administered. The term “fully artificial quasi-model” refers to the fact that, unlike pop-PK models which are based on drug concentrations measured in the blood of human subjects, a set of data, none of which actually represent the true characteristics of any human, is generated using a computer algorithm. This does not eliminate the need to collect data from humans, which remains crucial for establishing the modeled *ranges* of the random-effect pharmacokinetic variables, but the number of subjects required can be considerably lower. The workflow for constructing the quasi-models, and applying them to make individual parameter estimates, is displayed in Figure 1.

The nonparametric adaptive grid (NPAG) algorithm of Leary and Burke was employed for constructing mixed-effects pop-PK models based on the data obtained from human subjects. This iterative approach allows the nonparametric estimation of the joint population distribution of pharmacokinetic model parameter values by establishing a set of grid points of modest size, finding the maximum likelihood solution for that grid, and then refining the grid based on the optimal discrete so-called support points (i.e., vectors containing estimates of the pharmacokinetic parameter along with a probability value) and by adding a modestly sized set of new support points. The process continues until a convergence criterion defined by the modeler is reached, and no further improvement of the likelihood of the estimates of the random-effect variables can be attained [11,12,13].

In contrast to parametric modeling, which is based on the generation of measures of central tendency and dispersion, and the approximation of the likelihood function, NPAG relies on determining the exact likelihood function to describe the population, making the approach statistically consistent. No assumptions of the distributions of the random-effect variables are made, which makes NPAG superior in detecting subpopulations and outliers [12]. Nonparametric maximum a posteriori probability (MAP) Bayesian analysis in turn uses the pop-PK support points to find the pharmacokinetic parameters which apply to individual patients [14]. The utility of nonparametric pharmacokinetic modeling in the clinical setting has been demonstrated [15].

The modeled substance was piperacillin administered to critically ill adults diagnosed with community-acquired pneumonia, who were receiving treatment at the intensive care unit of a public hospital. This work is part of a larger clinical study conducted as described in a protocol published earlier, the overall aim of which is to establish a multidisciplinary methodology for the evaluation of pharmacological intervention in the first 5 days of the patient receiving intensive care for community-acquired pneumonia. The objectives include monitoring intra-individual changes in the pharmacokinetic properties of piperacillin and tazobactam, as well as in the concentrations of endogenous steroids and inflammatory markers which characterize the clinical status [16]. The pharmacokinetic modeling of the beta-lactamase was not performed since its serum concentrations were in strong correlation with those of piperacillin, which is in line with previous findings. There is no evidence that tazobactam concentrations should be taken into consideration when making a decision on the antibiotic regimen employed [17].

## 2. Materials and Methods

This investigator-initiated, unicentric, observational, one-arm study has been approved by the National Institute of Pharmacy and Nutrition (Budapest, Hungary, identifier of approval document: 261-IK/2020), the National Competent Authority of Hungary for medical research ethics. The Principal Investigator was Cs. Kopitkó. Twelve adults, admitted to the Central Department of Anesthesiology and Intensive Care, Uzsoki Teaching Hospital (Budapest, Hungary) with the diagnosis of community-acquired pneumonia, were recruited (Table 1). All subjects received standard care, including mechanical ventilation. The administration of a daily dose of 16 g (30.98 mmol) piperacillin + 2 g (6.66 mmol) tazobactam divided into four doses, given every 6 h as a 3-h intravenous infusion, was initiated empirically and immediately following admittance. No other antibiotics were given. Blood samples were collected by trained personnel in certified collection tubes (Greiner Bio-One Hungary Ltd., Budapest, Hungary) by accessing the vena cava superior, after finishing the first 8 AM infusion on the day following the day of admission, as described in [16]. Care was taken by the nurses of the Central Department of Anesthesiology and Intensive Care to adhere to all professional standards and institutional protocols, including drug administration and sample collection, as well as to document all activities and events related to the research, which was crucial for obtaining valid and credible outcomes. The sampling times, documented by the healthcare team, were 0.25 h, 0.5 h, 1 h, 1.5 h, 2 h and 2.5 h post-infusion. The samples were pretreated as necessary by the personnel of the Central Department of Anesthesiology and Intensive Care under the supervision of the Principal Investigator, and were subsequently sent to the laboratories where the various assays were performed. Native blood samples were kept at ambient temperature for no longer than 15 min, and were centrifuged thereafter at 10 °C and 2500× *g*, for 10 min. An aliquot of 0-h serum, as well as K_3_-EDTA-anticoagulated, heparinized and sodium citrate-treated whole blood samples were transferred to the Central Laboratory, Uzsoki Teaching Hospital, for routine laboratory assays, whole blood count and hematocrit measurement. A total of 250 µL serum separated for interleukin-6 measurement was frozen and sent to the Central Laboratory, Department of Laboratory Medicine, Semmelweis University (Budapest, Hungary). To 100 µL serum pipetted in a microcentrifuge tube, 20 µL Chromsystems Priming Solution (Cat. 61012) was added before freezing and transporting the samples for the evaluation of piperacillin and tazobactam concentrations at the Laboratory of Mass Spectrometry and Separation Technology, Department of Laboratory Medicine, Semmelweis University.

Piperacillin concentrations were determined using a Jasco series 4000 robust high-performance liquid chromatograph equipped with an MD-4010 photodiode array detector (ABL&E-JASCO Hungary Ltd., Budapest, Hungary). The Chromsystems^®^ Antibiotics in serum/plasma—HPLC in vitro diagnostic (CE-IVD) reagent kit, analytical column, multilevel calibrators and controls were employed for the analysis according to the instructions of the reagent kit manufacturer (ABL&E-JASCO Hungary Ltd., Budapest, Hungary). The analytical column was thermostatted at 30 °C, and the detection wavelength was 252 nm. The preparation of serum samples consisted of adding 200 µL of internal standard solution (supplied with the reagent kit) to the stabilized serum, vortexing the mixture at 2000 rpm for 1 min, separating the supernatant by centrifugation (10 °C, 10,000× *g*, 5 min), and diluting 100 µL of supernatant with 100 µL of dilution buffer (also supplied with the reagent kit). Calibration equations were obtained by performing 1/concentration^2^-weighted linear regression on piperacillin/internal standard peak area ratios. Internal controls were run at the beginning of each batch. In addition, the performance of the assay was tested regularly by participation in an external quality assessment scheme (#890—Antibiotics 02, Instand e.V., Düsseldorf, Germany).

The assay error polynomial (the fixed-effect component of the pop-PK models) was determined experimentally by quantitating piperacillin concentrations after spiking it in known concentrations to blank serum samples. Piperacillin was spiked at 20 different concentration levels and to 20 independent serum samples at each spiking level, in addition to the unspiked sera, all of which had been collected from different individuals for diagnostic purposes and had been left over from the laboratory tests. The de-identification of these samples had been performed, and no patient-related data were accessed by the authors. The standard deviation (SD) of measured piperacillin concentrations was calculated at each concentration level, including the blanks, and the regression of unweighted linear, second-degree and third-degree polynomials on the nominal concentration-SD data pairs was performed using Microsoft Excel to find the equation which could best describe this relationship, based on the work of Jelliffe and Tahani [18].

Nonparametric pharmacokinetic modeling was performed pursuant to the pioneering theoretical work of Roger W. Jelliffe and his co-workers, and by using the software tools they developed for this purpose. One- and two-compartment pop-PK models (#1 and #2) were constructed using 72 pieces of concentration data obtained from the 12 subjects recruited, and the NPAG algorithm incorporated into the Pmetrics^TM^ package run in the R environment (Laboratory of Applied Pharmacokinetics and Bioinformatics, University of Southern California, Los Angeles, CA, USA) [12,19]. The single-compartment models included the elimination rate constant (K) and the apparent volume of distribution (V), while the two-compartment models contained K, the rate constant of mass transfer from the central to the peripheral compartment (KCP) and of mass transfer from the peripheral to the central compartment (KPC), as well as the volume of the central compartment (V_c_) as random-effect variables. Single-compartment models take into account a hypothetical, equilibrated fluid compartment without anatomic reality in which the drug is distributed evenly, and eliminated in a single process regardless of its route, with the exception of cases where relevant covariates are included in the model to account for various specific routes. Two-compartment models represent a central and a peripheral fluid compartment. The central compartment refers to the intravascular water space as well as extravascular spaces which are rapidly equilibrated with it, and into which the transport of the drug is not limited. The peripheral compartment corresponds to fluid spaces which are accessed by the drug, and which can even accumulate the drug, but which are also not well equilibrated with the central compartment. Again, elimination is considered as a single-route process unless covariates describing the impact of various routes are included in the model.

The evaluation of the models’ performance was based on the strength of the correlation between predicted and observed concentrations, the slope and intercept of the regression line fit to these pairs of concentrations, as well as their weighted squared residuals (bias) and the bias-adjusted weighted squared residuals (imprecision). The performance indicators −2 × log-likelihood, and Akaike and Bayesian information criteria were also assessed. The decision to include candidate covariates or not was made by investigating their linear correlation, as well as the linear correlation of the square roots and the natural logarithms of their values, with the posteriors of the random-effect pharmacokinetic variables: K, KCP/KPC and V or V_c_. A Pearson’s correlation coefficient of at least 0.80 was considered strong enough for inclusion. One- and two-compartment models of piperacillin (models #3 and #4, respectively) were subsequently constructed by including creatinine clearance as a covariate, calculated as proposed by Jelliffe, and by making an estimate of the elimination rate constant by using the function K = KI + KS × CRCL where KI is the rate constant accounting for non-renal elimination, KS is the rate constant accounting for renal elimination, and CRCL is the creatinine clearance [20,21]. KI and KS were the random-effect variables in these models.

Individual pharmacokinetic parameters (IPKP) of each subject were estimated in NPAG runs conducted using Pmetrics^TM^ (version 2.1, IPKP-NPAG), as well as by using the BestDose^TM^ standalone clinical pharmacokinetic modeling software (desktop version 1.127b, IPKP-NPB) which performs nonparametric maximum a posteriori probability (MAP) Bayesian analysis (Laboratory of Applied Pharmacokinetics and Bioinformatics, University of Southern California, Los Angeles, CA, USA). Fully artificial quasi-models were constructed by generating 399 random values with uniform distribution for each random-effect variable, and by creating 399 support points which contained a random value assigned to each parameter in the order they were created in, as well as a probability value of 1/399. The amount of the support points generated was the highest allowed by the BestDose^TM^ software. The ranges the random values were generated in were made equivalent to those employed for the priors entered into the NPAG models. The dosing error, the model misspecification, and the timing error were set to 0.01. A total of 30 QMs were created for each type of PK model, and their performance was compared by calculating the mean squared errors of the predictions: ∑(c_obs_ − c_pred_)^2^/n_dp_, where n_dp_ stands for the number of data points; in the present work, n_dp_ = 6.

## 3. Results

### 3.1. Analytical Considerations

**Figure 2:**
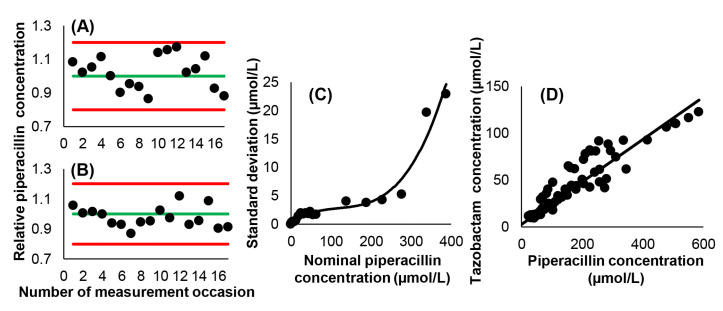
Characteristics of the employed piperacillin HPLC-UV assay. (**A**) Ratios of the measured and nominal concentrations of piperacillin in the low-level internal control samples. The green line corresponds to the nominal concentration, while red lines represent the limits of measurement acceptability. (**B**) Ratios of the measured and nominal concentrations of piperacillin in the high-level internal control samples. (**C**) Relationship between piperacillin concentration and the standard deviation of measurement results. (**D**) Relationship between piperacillin and tazobactam concentrations. Ordinary linear least squares regression yielded a Pearson’s correlation coefficient of r = 0.9245.

The performance of the piperacillin assay was monitored in each run. The internal control measurements yielded acceptable results in terms of the reagent kit manufacturer’s specifications. A nonlinear relationship existed between the concentrations of piperacillin and their standard deviations, and was defined by the third-degree polynomial SD = 0.255056 + 0.049873 × c *−* 0.000361 × c^2^ + 0.000001 × c^3^ with a determination coefficient of r^2^ = 0.9564 (where c is the concentration of piperacillin, Figure 2). The correlation between piperacillin and tazobactam concentrations was strong, confirming previous findings, and could be described with the equation c_tazobactam_ = 0.2267 × c_piperacillin_ + 2.6802 [17]. The slope of this equation displayed very close correspondence to 0.2150; which is the molar ratio of the administered drugs.

### 3.2. Population Pharmacokinetic Models

**Figure 3:**
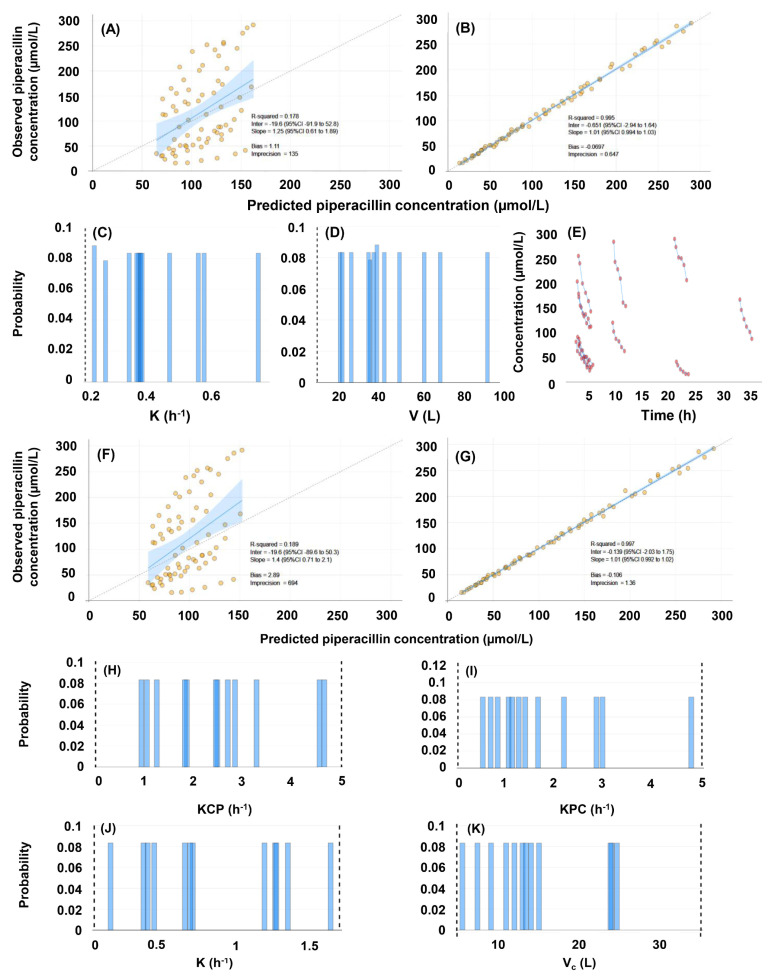
Evaluation of the population pharmacokinetic models of piperacillin, constructed with the inclusion of 72 concentrations obtained in 12 human subjects, and with no covariate. (**A**–**E**), evaluation of the single-compartment model (#1). (**A**) Comparison of predicted and observed concentrations based on population priors. (**B**) Comparison of predicted and observed concentrations based on individual posteriors. (**C**) Marginals of the elimination rate constant. (**D**) Marginals of the volume of distribution. (**E**) Raw concentration-time plots. (**F**–**K**), evaluation of the two-compartment model (#2). (**F**) Comparison of predicted and observed concentrations based on population priors. (**G**) Comparison of predicted and observed concentrations based on individual posteriors. (**H**–**I**) Marginals of the intercompartmental mass transfer rate constants: (**H**) central to peripheral compartment, (**I**) peripheral to central compartment. (**J**) Marginals of the elimination rate constant. (**K**) Marginals of the volume of distribution.

**Figure 4:**
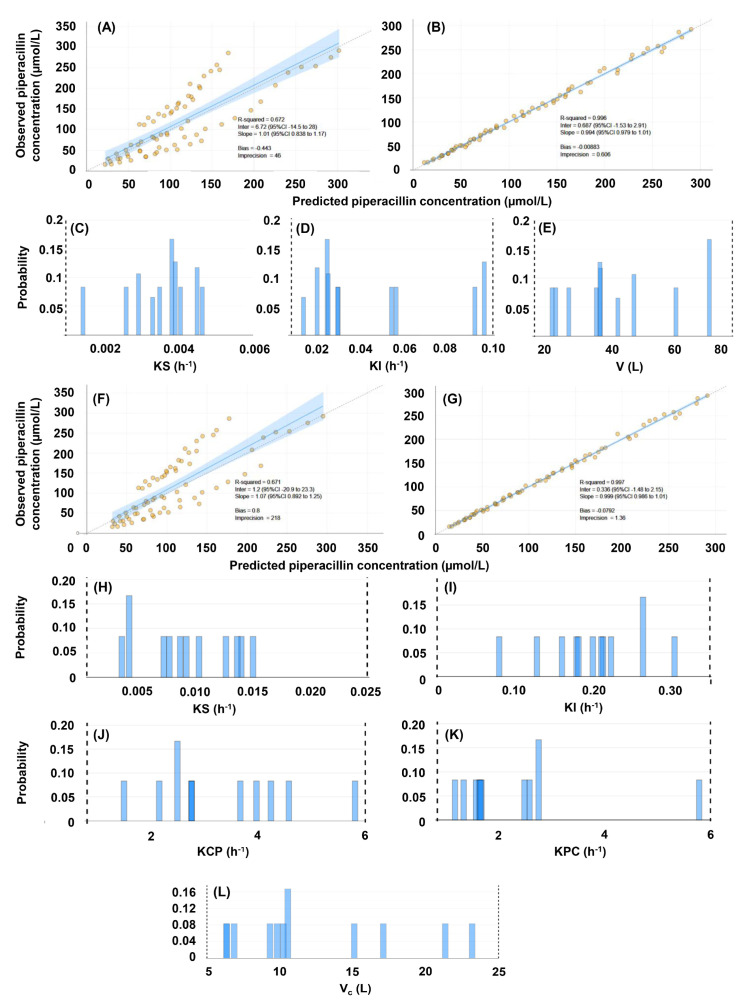
Evaluation of the population pharmacokinetic models of piperacillin, constructed with the inclusion of 72 concentrations obtained in 12 human subjects, and with the inclusion of creatinine clearance as a covariate. (**A**–**E**), evaluation of the single-compartment model (#3). (**A**) Comparison of predicted and observed concentrations based on population priors. (**B**) Comparison of predicted and observed concentrations based on individual posteriors. (**C**) Marginals of the renal elimination rate constant. (**D**) Marginals of the non-renal elimination rate constant. (**E**) Marginals of the volume of the central compartment. (**F**–**L**), evaluation of the two-compartment model (#4). (**F**) Comparison of predicted and observed concentrations based on population priors. (**G**) Comparison of predicted and observed concentrations based on individual posteriors. (**H**) Marginals of the renal elimination rate constant. (**J**) Marginals of the rate constant of mass transfer from the central to the peripheral compartment. (**K**) Marginals of the rate constant of mass transfer from the peripheral to the central compartment. (**L**) Marginals of the volume of the central compartment.

The summary of the performance characteristics of the constructed population pharmacokinetic models #1–#4 is demonstrated in Table 2, as well as in Figure 3 and Figure 4. All models performed well in terms of the agreement between observed and estimated posterior concentrations (slopes: 0.994–1.01, intercepts: −0.651–0.687, r^2^ = 0.995–0.997), bias and imprecision. A statistically significant difference was found between one-and two-compartment models generated either without or with the inclusion of creatinine clearance as a covariate (*p* < 0.001). No statistical impact of including creatinine clearance as a covariate was identified, irrespective of the number of pharmacokinetic compartments. The posterior ranges of the random-effect pharmacokinetic variables which were subsequently considered when generating the random values for the support points of the QMs are displayed in Table 3.

### 3.3. Individual Pharmacokinetic Models

**Figure 5:**
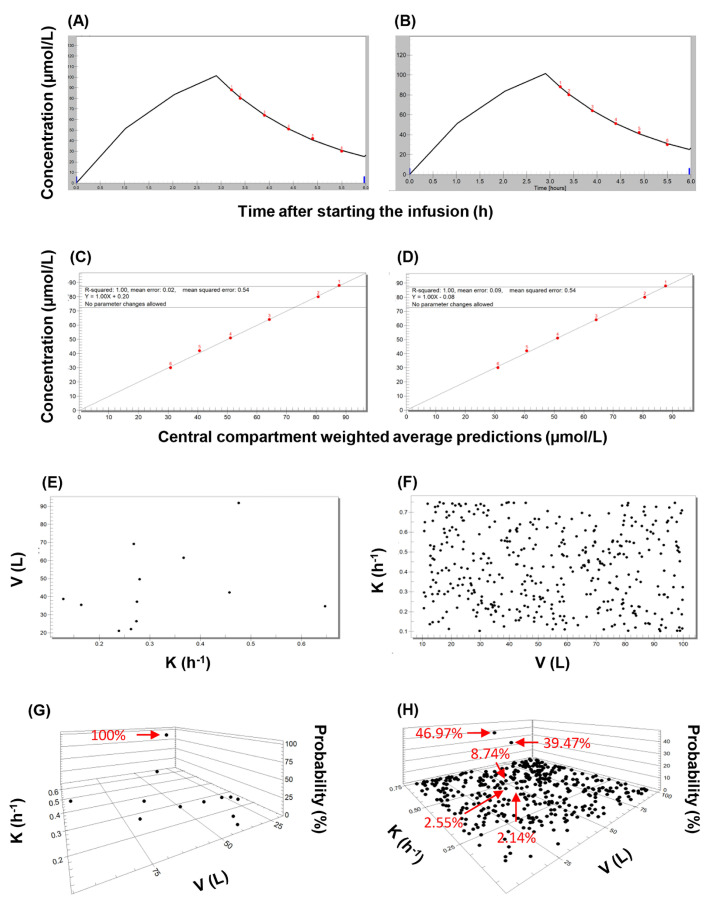
An overview of the main features of applying the nonparametric expectation maximization algorithm to population-based and quasi-models. The evaluation of piperacillin concentrations observed in subject 1 is shown as an illustration. (**A**) Concentration-time plot showing the observed values (red dots), and the curve fitted using the single-compartment population pharmacokinetic model with no covariate (model #1). (**B**) Concentration-time plot showing the observed values (red dots), and the curve fitted using the best-performing single-compartment quasi-model. (**C**) Correlation plot of observed piperacillin concentrations and those predicted using population pharmacokinetic model #1. (**D**) Correlation plot of observed piperacillin concentrations and those predicted using the best-performing single-compartment quasi-model. (**E**) Two-dimensional plot showing the support points of model #1. (**F**) Two-dimensional plot showing the support points of the best-performing single-compartment quasi-model employed as priors. (**G**) Three-dimensional plot of the posterior support points of model #1. (**H**) Three-dimensional plot of the posterior support points of the best-performing single-compartment quasi-model.

Exemplary results of fitting the QMs to the individual concentration data series are displayed in Figure 5. The support points generated using random values covered the entire parameter space. When pop-PK models were used as priors, the posterior probabilities of only one or two support points were increased. When models #1 and #2 were applied, the support point with the increased probability corresponded to that generated for the given individual using NPAG. On the contrary, high posterior probabilities were obtained for several support points when applying the quasi-models, especially when two-compartment models were fitted.

**Figure 6:**
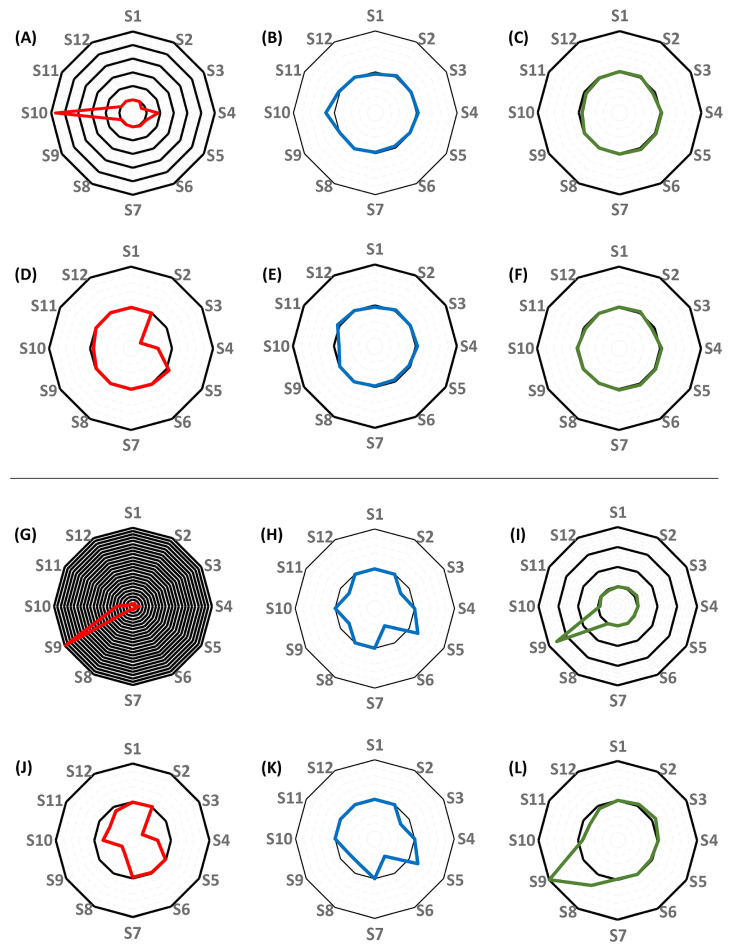
Radar plots showing the differences between the performance of the best-performing single-compartment quasi-models and of the single-compartment population pharmacokinetic models (#1 and #3) for each subject. (**A**–**F**), comparison of the performance of models constructed without the inclusion of creatinine clearance as a covariate. (**A**–**C**), the comparison of the mean squared errors (MSE) of predictions (**A**), the elimination rate constants (**B**), and the volumes of distribution (**C**) obtained by applying the best-performing quasi-models to the estimates obtained using nonparametric adaptive grid (NPAG) modeling and the population PK models. (**D**,**F**), the comparison of the MSE’s of predictions (**D**), the elimination rate constants (**E**), and the volumes of distribution (**F**) obtained by applying the best-performing quasi-models to the estimates obtained when conducting nonparametric maximum a posteriori probability (MAP) Bayesian analysis on the population PK models. (**G**–**L**), comparison of the performance of models constructed with the inclusion of creatinine clearance as a covariate. (**G**–**I**), the comparison of the MSE’s of predictions (**G**), the elimination rate constants (**H**), and the volumes of distribution (**I**) obtained by applying the best-performing quasi-models to the estimates obtained using NPAG and the population PK models. (**J**–**L**), the comparison of the MSEs of predictions (**J**), the elimination rate constants (**K**), and the volumes of distribution (**L**) obtained by applying the best-performing quasi-models to the estimates obtained when conducting MAP Bayesian analysis on the population PK models. Each black circle represents an additional 100% increase in the ratio of values obtained by applying the best-performing quasi-models and of values obtained by applying the NPAG or the MAP Bayesian algorithm to the population PK models, with the central black circle corresponding to 100% agreement.

**Figure 7:**
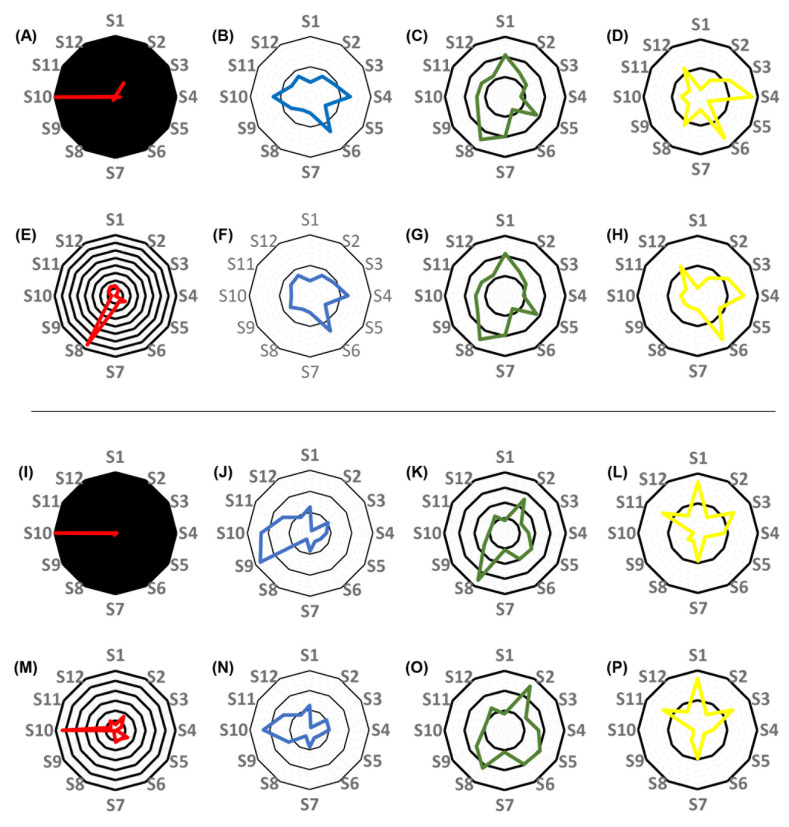
Radar plots showing the differences between the performance of the best-performing two-compartment quasi-models and of the two-compartment population pharmacokinetic models (#2 and #4) for each subject. (**A**–**H**), comparison of the performance of models constructed without the inclusion of creatinine clearance as a covariate. (**A**–**D**), the comparison of the mean squared errors (MSE) of predictions (**A**), the elimination rate constants (**B**), the volumes of the central compartment (**C**), and the ratios of the central-to peripheral compartment and the peripheral-to-central compartment mass transfer rate constants (KCP/KPC ratio, (**D**)) obtained by applying the best-performing quasi-models to the estimates obtained when applying nonparametric adaptive grid (NPAG) modeling and the population PK data. (**E**–**H**), the comparison of the MSEs of predictions (E), the elimination rate constants (**F**), the volumes of the central compartment (**G**), and the KCP/KPC ratios (**H**) obtained by applying the best-performing quasi-models to the estimates obtained when applying nonparametric maximum a posteriori probability (MAP) Bayesian analysis on the population PK data. (**I**–**P**), comparison of the performance of models constructed with the inclusion of creatinine clearance as a covariate. (**I**–**L**), the comparison of the MSEs of predictions (**I**), the elimination rate constants (**J**), the volumes of the central compartment (**K**), and the KCP/KPC ratios (**L**) obtained by applying the best-performing quasi-models to the estimates obtained when applying NPAG algorithm and the population PK data. (**M**–**P**), the comparison of the MSEs of predictions (**M**), the elimination rate constants (**N**), the volumes of the central compartment (**O**), and the ratios of the KCP/KPC ratios (**P**) obtained employing the best-performing quasi-models to the estimates obtained when applying MAP Bayesian analysis on the population PK data. Each black circle represents an additional 100% increase in the ratio of values obtained by applying the best-performing quasi-models and of values obtained by applying the NPAG or the MAP Bayesian algorithm to the population PK models, with the central black circle corresponding to 100% agreement.

The mean squared errors, as well as the estimates of the random-effect pharmaco-kinetic variables obtained in each subject by applying the best-performing QMs, were compared to those obtained by using the support points of the pop-PK models as priors, and either the NPAG or the MAP Bayesian algorithm. The results of these comparisons are displayed in Table 4 and visualized in Figure 6 and Figure 7. When a single-compartment model with no covariate was employed, the MSEs obtained using the respective best-performing quasi-models ranged between 0.70 and 1.08 for 10 out of 12 subjects. The comparison of the MSEs with those obtained when using the pop-PK model support points as priors yielded IPKP-QM/IPKP-NPAG and IPKP-QM/IPKP-NPB ratios of 0.70–1.08 and 0.27–1.07, respectively. A ratio of 0.01–1.00 indicates better performance of the quasi-model-based predictions, a ratio of 1.00 corresponds to equivalence, while a ratio of >1.00 corresponds to better performance of the estimates made by using the pop-PK model support points. Each 0.01 increment corresponds to an additional 1% difference. The ratios of the individual elimination rate constants were 0.95–1.21 and 0.88–1.05, respectively, while the ratios of the volumes of distribution were 0.91–1.04 and 0.93–1.04, respectively. When creatinine clearance was included as a covariate in the single compartment model, the MSE ratios were 0.37–1.83 and 0.29–1.01, respectively. The ratios of the renal component of the elimination rate constant (KS) were 0.50–1.25 in both comparisons. The ratios of V ranged between 0.89–1.10 and 0.81–1.32, respectively, for 11 of the 12 subjects. When a two-compartment model with no covariate was applied, the MSE ratios were 0.09–111 and 0.01–1.60, respectively. The ratios of K were 0.49–1.33 in both comparisons, while the ratios of the estimated V_c_ were 0.75–2.45 and 0.75–2.49, respectively. The KCP/KPC ratios ranged between 0.27 and 1.80, and 0.27 and 1.66, respectively. Finally, when a two-compartment model with a creatinine clearance covariate was employed, the MSE ratios were 1.02–5.47 and 0.05–1.61, respectively (with the exception of subject 10, for whom the MSE ratios obtained were 181 and 5.33, respectively). The ratios of KS were 0.14–2.75 and 0.14–2.33, and the ratios of the estimated V_c_ were 0.84–3.55 and 0.83–2.56, respectively. The KCP/KPC ratios were 0.15–1.70 and 0.15–1.72, respectively. The comprehensive evaluation of the performance of QMs is provided in the Supplementary File.

The concentration curves fitted by nonparametric MAP Bayesian analysis using the best-performing individual QMs as priors, along with the quality of the fits, are available in the Supplementary File. The determination coefficients of the relationship between the predicted and observed piperacillin concentrations were 0.933–0.999, 0.922–0.999, 0.935–0.999 and 0.935–0.999 when applying model #1–#4, respectively. Considerable mismatches between predicted and observed concentrations were observed when two-compartment QMs were applied with no covariate for the data of subject 9. In the case of subject 10, the determination coefficients obtained when single-compartment models (model #1 and model #3) were applied were 0.831–0.834 and 0.833–0.834, respectively. The underlying reason for the weaker agreement appeared to be a single sample (sample 2) for which the measured piperacillin concentration was consistently lower than predicted. Two-compartment quasi-models were clearly not suitable for this patient, as reflected by the determination coefficients 0.292–0.568 and 0.359–0.513 obtained for models #2 and #4, respectively. The poor agreement between predicted and observed values could be traced back to the algorithm included in the nonparametric MAP Bayesian analysis; when the pop-PK models were employed as priors, running NPAG resulted in determination coefficients of 0.985–0.987, whereas the application of the MAP Bayesian algorithm resulted in coefficients of 0.136–0.831.

## 4. Discussion

Several piperacillin population pharmacokinetic models are available in the literature, predominantly with the consideration of single- or, more often, two-compartment models including creatinine clearance as a covariate [22,23,24]. Nevertheless, it has recently become apparent that the suitability of these models for making estimates of individual pharmacokinetic properties can be highly variable. A multicenter study revealed only three models which provided acceptable estimates and absolute prediction errors. Interestingly, the authors of this study concluded that the accuracy of estimates was gender-dependent [24].

Nonparametric pharmacokinetic modeling is a powerful tool for guiding individualized drug therapy. The pharmacokinetic characteristics of the individuals included in the modeling process are retained instead of constructing statistical summaries which do not show individual values. Consequently, the identification of subpopulations and individual outliers, which are often of special clinical interest, is feasible. The evaluation and comparison of nonparametric models is straightforward, and the limitations of the constructed models are transparent.

Multimodel approaches have been proposed to improve individual estimates for drugs such as tacrolimus in liver, lung and bowel transplanted patients. For each individual, a model could be selected by employing a weight-based algorithm which compared the median prediction error of each model to that of a set of nine models picked randomly from a library consisting of 70 population models [25]. Such efforts can be supported further by employing models augmented by computer algorithms. Mao et al. demonstrated the efficiency of models based on machine learning (ML), specifically of those built using an artificial neural network algorithm, to be superior to that of population pharmacokinetic models when estimating cyclosporin A concentrations [8]. Hybrid pop-PK-ML models have been shown to have a performance superior to that of pop-PK models alone with iohexol and isavuconazole [26,27]. In addition to improving modeling performance, the tremendous saving on modeling time offered by ML algorithms is an important advantage [28]. Such efforts indicate that the application of ML-based approaches, should they rely on the integrated analysis of several pop-PK models or on artificial algorithms, could be the next important step towards the implementation of personalized drug therapy.

The methodology presented in this work is similar to an earlier concept created by Jelliffe et al., and based on hybrid Bayesian analysis [29]. Jelliffe et al. proposed the augmentation of nonparametric population PK model support points with further ones, generated by the modeler. These additional support points were evenly distributed in a parameter subspace (not necessarily within the parameter space defined by the pop-PK model), formed a symmetric grid, and were assigned equal probabilities. Our methodology relies on the generation of random support points with equal probabilities (currently within the parameter space defined by the population PK model), and on building multiple models. This approach is based on a simple computing algorithm, and is not bound by the set of support points of the pop-PK model. Instead of employing the conventional workflow of generating a population model, and then applying it to individual data, any number of such QMs can be generated and tested until a set of support points displaying acceptable performance in estimating drug exposure in the given patient is found. An important advantage of employing QMs is the simplicity and the low time-consumption of their generation, as well as the transparency of their operation. In most of our analyses, the performance of the best-performing QMs was equivalent to, or better than that of MAP Bayesian analysis or of the NPAG model; although the latter actually contained the set of the estimated pharmacokinetic properties of each individual. Two-compartment models of piperacillin were superior in performance to single-compartment models when considering statistical indicators at population level but, in view of the MSE’s obtained, could not be favored in all individuals. Our results demonstrate that the type of pharmacokinetic model which provides the best performance may have to be determined for each individual. As an example, a two-compartment model could not be fitted to the data of subject 10, while a single-compartment model with creatinine clearance included as a covariate provided considerably worse fits for subject 9 in comparison to all other models employed. This indicates that flexibility in selecting the most suitable type of the PK model from a set of models in each individual case may be relevant to clinical practice, and also highlights the importance of collecting a set of blood samples from each patient which is sufficiently large for comparing the performance of various PK models (Table 5).

Usually, the subjects are divided into a training set and a test (or validation) set when evaluating the performance of a pharmacokinetic model [30]. In the presented work, the training and testing set had to be the same due to the fact that the support points of the pop-PK models and the QMs were independent of each other. The NPAG algorithm employs several thousand support points for selecting the best fit; therefore, it is extremely reliable in making the most efficient estimates in a setting of rich sampling such as the one employed in the present work. The results show that the differences in IPKP-NPAG versus IPKP-NPB estimates were larger than those obtained for IPKP-NPB versus IPKP-QM estimates. The performance of QMs compared to that of the pop-PK models when employing the MAP Bayesian algorithm provides evidence that they are efficient alternatives to pop-PK models.

The inclusion of creatinine clearance as a covariate did not lead to the improvement of the pharmacokinetic models (Table 2). When CRCL was included in the single-compartment model #3, the shrinkage was considerably higher than that observed for model #1. Since this effect of the inclusion of the CRCL covariate was not observed in the case of two-compartment models (#2 and #4), it seems rational to conclude that the result of testing this covariate was helpful in model selection. It is rational to assume that the importance of including CRCL as a covariate may become even more apparent when the course of piperacillin pharmacokinetics is monitored over several days, considering the fact that the renal function of critically ill patients is often unstable.

Creatinine clearance was estimated using the equation described by Jelliffe, which allows calculation without measuring urinary creatinine levels. This equation was incorporated into the BestDose^TM^ software. Calculation is based on Equation (1):

(1) $$\frac{0.4\times BW\times (c_{2}-c_{1})}{T}=P_{adj}-\frac{c_{1}+c_{2}}{2}\times CCR\times 1440$$

where BW corresponds to body weight in kg, c_1_ and c_2_ are the first and second serum creatinine concentrations in mg/dL, respectively, P_adj_ is the adjusted creatinine production in mg and CCR is the creatinine clearance in hundreds of mL per minute [20]. It must be noted that estimating creatinine clearance by measuring serum creatinine is not an optimal approach in the critically ill, as sarcopenia or a poor clinical status influence serum creatinine concentrations [31]. Furthermore, creatinine is not only filtered, but also secreted by the kidneys [32]. The impact of changes in the clinical status on serum creatinine can be detected with a delay of 24–36 h, and only when at least half of the nephrons have ceased to function. Since the present work focused on piperacillin pharmacokinetics in the first 24 h of intensive care, these factors are not expected to have influenced our findings.

Evaluating inulin clearance is often considered as the gold standard for characterizing the glomerular filtration rate, but is impractical in the clinical setting [33]. Various biomarkers (such as cystatin C) have been proposed, and it seems that multimarker panels may be superior to single laboratory parameters in this respect [34]. An important yet challenging area of future research is the incorporation of novel biomarkers of renal function in pharmacokinetic models, especially in unstable, critically ill patients. Jelliffe’s formula is valuable as it takes the instability of renal function into account, and is compatible with interacting multiple modeling, which is an efficient approach to describing even the rapidly changing pharmacokinetics of a drug in unstable patients [35]. Combined with QMs, this approach may be highly useful in detecting changes in the clinical status of critically ill patients receiving piperacillin.

The following limitations apply to the results presented: (1) the efficiency of QMs was tested only within the ranges of the random-effect pharmacokinetic variables established in the NPAG runs; (2) the number of subjects included in the study was small, and model optimization was not performed by, for example, evaluating the inclusion of covariates other than creatinine clearance; and (3) the QMs did not allow the inference of real pharmacokinetic information on the population involved in the modeling process. The goal of making efficient individual estimates was nevertheless attained; therefore, the QMs have the potential to be applicable for predicting future concentrations in individuals, which is the most important aim in the clinical setting. From a clinical standpoint, a minor limitation is that obtaining six concentration points can be difficult in real clinical scenarios. A further exploration of the performance of the QMs on sets of less richly samples patients will, therefore, be necessary.

Finally, the extensive experimental elaboration of the assay error polynomial proved to be essential for achieving the described performance of the QMs. While the options of an additive or a multiplicative error model are available when running NPAG, and the definition of a higher value for the respective constant may compensate for a less accurate assay error model, the definition of additional error terms is more complex in the BestDose^TM^ software, and the compensation may require entering unrealistic values for dosing and sampling error, or may not be possible at all. Most often, linear equations and second-degree polynomials can be applied to describe the quantitative relationship between analyte concentration and the method standard deviation of liquid chromatography-tandem mass spectrometry methods and immunoassays, respectively. In our case, a third-degree polynomial fit was clearly appropriate for the employed HPLC-UV assay [36,37].

## 5. Conclusions

The joint concept of rich sampling and the use of fully artificial nonparametric quasi-models can provide an efficient augmentation tool for planning and monitoring individualized drug regimens, and may therefore be considered as an alternative to limited sampling strategies employed when a larger set of subjects is available. Nonparametric QMs can be especially useful when the number of subjects available for pop-PK modeling is small, which is a common scenario in the clinical setting, especially in the case of drugs which have been introduced to the market only recently.

The performance of two-compartment pharmacokinetic models has proved to be superior to that of single-compartment models, pointing to the benefit of rich sampling. Including creatinine clearance as a covariate did not lead to a significant improvement in model performance. Nevertheless, such models may be valuable in the future when longitudinal analyses are conducted in patients with unstable renal function.

## Figures and Tables

**Table 1:** Demographic properties of the subjects included in the study. Values or medians with ranges in parentheses are displayed. ICU, intensive care unit.

Characteristic	Value
Number of subjects	12
Age (years)	69.7 (45.3–86.4)
Male gender (%)	58
APACHE II score on admission to ICU (no unit)	25 (19–37)
CURB-65 mortality score on admission to ICU (no unit)	6.8 (2.7–27.8)
SAPS-E mortality score on admission to ICU (no unit)	42.3 (7.9–59.7)
SOFA mortality score on admission to ICU (no unit)	33.3 (33.3–50.0)
Body mass index on admission to ICU (kg/m^2^)	29.6 (24.2–51.9)
Mean arterial pressure (mm Hg)	73.7 (56.7–120.7)
Serum creatinine (µmol/L)	98 (34–224)
Sodium (mmol/L)	137 (135–144)
Potassium (mmol/L)	4.4 (3.6–5.8)
Glucose (mmol/L)	9.1 (5.6–13.7)
Urea (mmol/L)	11.1 (2.6–41.8)
Total bilirubin (µmol/L)	15.9 (5.5–82.3)
Procalcitonin (µg/L)	0.5 (0.0–126.6)
C-reactive protein (mg/L)	129.4 (7.5–546.8)
White blood cell count (×10^9^/L)	17.0 (9.0–31.1)
Thrombocyte count (×10^3^/L)	274 (86–714)
Serum lactate (mmol/L)	1.8 (1.1–3.0)
Base excess (mEq/L)	5.0 (−8.4–13.7)
Hematocrit (L/L)	0.4 (0.3–0.7)
Interleukin-6 (ng/L)	32.0 (4.8–3629.0)
Pharmacokinetically relevant drugs administered on the day of blood sample collection (% of subjects):	
Dexmedetomidine	8.3
Alprazolam	16.6
Methylprednisolone	25.0
Ibuprofen	8.3
Fentanyl	8.3
Norepinephrine	66.7

**Table 2:** Performance of the constructed population pharmacokinetic models. AIC, Akaike information criterion. BIC, Bayesian information criterion. CRCL, creatinine clearance. LL, log-likelihood. #SP, number of support points.

Model No.	Compart-ments	Cova-riate	#SP	Bias (*p*-Value of Difference from 0)	Impre-cision	−2 × LL	AIC	BIC	Shrinkage (%)
#1	1	None	12	−0.0697 (0.6591)	0.6465	478.8	485.2	491.6	0.030–0.012
#2	2	None	12	−0.1061 (0.8310)	1.3578	423.5	434.4	444.8	0.000–0.002
#3	1	CRCL	10	−0.0088 (0.8637)	0.6061	474.4	483.0	491.5	0.348–14.91
#4	2	CRCL	11	−0.0792 (0.6000)	1.3616	422.4	435.7	448.1	0.000–0.006

**Table 3:** Posterior ranges of the random-effect pharmacokinetic variables considered for generating the quasi-models. K, elimination rate constant. KCP, rate constant of mass transfer from the central to the peripheral compartment. KPC, rate constant of mass transfer from the peripheral to the central compartment. KI, non-renal elimination rate constant. KS, renal elimination rate constant. V, volume of distribution. V_c_, volume of the central compartment.

Models with No Covariate Included																																									
Model No.							Compartments							K (1/h)							V (L) or V_c_ (L)							KCP (1/h)							KPC (1/h)						
#1							1							0.10–0.75							10–100																				
#2							2							0.10–1.70							5–35							0.05–5.00							0.05–5.00						
**Models with Creatinine Clearance Included as a Covariate**																																									
**Model No.**						**Compartments**						**KS (1/h)**						**KI (1/h)**						**V (L) or V_c_ (L)**						**KCP (1/h)**						**KPC (1/h)**					
#3						1						0.001–0.006						0.005–0.100						15–80																	
#4						2						0.0005–0.0250						0.00–0.35						5–25						0.2–6.0						0.2–6.0					

**Table 4:** Comparison of the performance of quasi-models (QM) and population pharmacokinetic (pop-PK) models when applying nonparametric adaptive grid (NPAG) modeling, or nonparametric maximum a posteriori probability (MAP) Bayesian analysis. Ratios of the mean squared errors (MSE) and of pharmacokinetic parameters are shown. In the case of MSE, a ratio of 0.01–1.00 corresponds to the superior performance of the quasi-model-based estimation, a ratio of 1.00 corresponds to equivalence, while a ratio of >1.00 corresponds to the better performance of the pop-PK model-based estimation; each 0.01 increment corresponds to an additional 1% difference. K, elimination rate constant. KCP, rate constant of mass transfer from the central to the peripheral compartment. KPC, rate constant of mass transfer from the peripheral to the central compartment. KS, renal elimination rate constant. V, apparent volume of distribution. Vc, apparent volume of the central compartment.

Model	Comparator	Value Obtained for QM/Value Obtained for pop-PK Model, NPAG	Value Obtained for QM/Value Obtained for pop-PK Model, MAP Bayesian Analysis
#1	MSE	0.70–1.08 (Subject 4: 1.83, Subject 10: 5.67)	0.27–1.07
	K	0.95–1.21	0.88–1.05
	V	0.91–1.04	0.93–1.04
#2	MSE	0.09–111	0.01–1.60 (Subject 8: 7.40)
	K	0.49–1.33	0.49–1.33
	Vc	0.75–2.45	0.75–2.49
	KCP/KPC	0.27–1.80	0.27–1.66
#3	MSE	0.37–1.83 (Subject 9: 32.54, Subject 10: 4.89)	0.29–1.01
	KS	0.50–1.25	0.50–1.25
	V	0.89–1.10 (Subject 9: 3.55)	0.81–1.32 (Subject 9: 1.99)
#4	MSE	1.02–5.47 (Subject 10: 181)	0.05–1.61 (Subject 10: 5.33)
	KS	0.14–2.75	0.14–2.33
	Vc	0.84–3.55	0.83–2.56
	KCP/KPC	0.15–1.70	0.15–1.72

**Table 5:** Mean squared errors obtained for each subject using the best-performing quasi-model. CRCL, creatinine clearance.

	Mean Squared Errors of the Quasi-Models Showing Best Performance			
Number of Subject	One Compartment, No Covariate	One Compartment, CRCL Covariate	Two Compartments, No Covariate	Two Compartments, CRCL Covariate
1	0.538	0.534	0.679	0.629
2	22.552	22.853	15.219	16.599
3	1.623	1.727	0.755	1.196
4	66.147	65.318	9.096	13.452
5	14.362	13.305	5.292	5.968
6	49.163	47.331	46.657	46.923
7	16.046	15.458	13.018	8.677
8	22.919	22.909	28.703	24.227
9	26.125	612.196	18.442	23.742
10	25.978	25.999	455.938	743.067
11	99.281	99.798	96.929	94.461
12	10.755	10.732	6.882	5.302

## Data Availability

All relevant data are contained in the article.

## Supplementary Materials

The following supporting information can be downloaded at: https://www.mdpi.com/article/10.3390/pharmaceutics16030358/s1, Figure S1. Individual concentration-time curves obtained when applying the best-performing quasi-models to the observed piperacillin concentrations. Each row shows the curves fitted, along with the correlation plots of the observed and predicted concentrations for a Subject (S1–S12). The horizontal and vertical axes of the fitted curve plots represent time (h) and piperacillin concentration (µmol/L), respectively. The origin (time = 0 h) corresponds to the time of the initiation of treatment with piperacillin/tazobactam. The axes of the correlation plots are predicted and observed piperacillin concentrations (µmol/L). CRCL, creatinine clearance. Table S1. Coefficients of correlation between the values of the covariates tested, and the pharmacokinetic variables modeled. K, elimination rate constant. KCP, rate constant of the mass transfer from the central to the peripheral compartmetnt. KPC, rate constant of the mass transfer from the peripheral to the central compartment. V, Volume of distribution. Vc, volume of the central compartment. Table S2: Performance of the quasi-models for Subject 1; Table S3: Performance of the quasi-models for Subject 2; Table S4: Performance of the quasi-models for Subject 3; Table S5: Performance of the quasi-models for Subject 4; Table S6: Performance of the quasi-models for Subject 5; Table S7: Performance of the quasi-models for Subject 6; Table S8: Performance of the quasi-models for Subject 7; Table S9: Performance of the quasi-models for Subject 8; Table S10: Performance of the quasi-models for Subject 9; Table S11: Performance of the quasi-models for Subject 10; Table S12: Performance of the quasi-models for Subject 11; Table S13: Performance of the quasi-models for Subject 12.
