# Supplementary material for: Modeling Pharmacokinetics in Individual Patients Using Therapeutic Drug Monitoring and Artificial Population Quasi-Models: A Study with Piperacillin
Source: Pharmaceutics. 2024 Mar 4;16(3):358. doi: 10.3390/pharmaceutics16030358 (PMC10975186; doi:10.3390/pharmaceutics16030358)
Supplement: Supplementary file 1 [file pharmaceutics-16-00358-s001.zip › pharmaceutics-2663802-supplementary.pdf]

Supplementary Material

# Modeling Pharmacokinetics in Individual Patients Using Therapeutic Drug Monitoring and Artificial Population Quasi-Models: A Study with Piperacillin

Gellért Balázs Karvaly, István Vincze, Michael Noel Neely, István Zátroch, Zsuzsanna Nagy, Ibolya Kocsis and Csaba Kopitkó

## 1. Source code for modeling using Pmetrics:

```
Library(Pmetrics)


# Run 1 - 1-compt, no covariate

pipdata <- PM_data$new(data = "data.csv")

pipmodel.onecompt.1 <- PM_model$new("model.txt")

pip.onecompt.1 <- PM_fit$new(model = pipmodel.onecompt.1, data =
pipdata)

pip.onecompt.1$run(cycles=5000)


# Run 2 - 2-compt, no covariate

pipmodel.twocompt.1 <- PM_model$new("model.txt")

pip.twocompt.1 <- PM_fit$new(model = pipmodel.twocompt.1, data =
pipdata)

pip.twocompt.1$run(cycles=5000)


# Run 3 - 1-compt, covariate: CRCL

pipmodel.onecompt.cov.1 <- PM_model$new("model.txt")

pip.onecompt.cov.1 <- PM_fit$new(model = pipmodel.onecompt.cov.1,
data = pipdata)

pip.onecompt.cov.1$run(cycles=5000)


# Run 4 - 2-compt, covariate: CRCL

pipmodel.twocompt.cov.1 <- PM_model$new("model.txt")

pip.twocompt.cov.1 <- PM_fit$new(model = pipmodel.twocompt.cov.1,
data = pipdata)

pip.twocompt.cov.1$run(cycles=5000)
```

```
# Evaluation of models

pip.subj=NULL

pip.1ct.data = PM_load(1)

pip.1ct.sum=pip.1ct.data$op$summary(pred.type =
"post",icen="median")

pip.1ct.post = pip.1ct.data$final$data$postMed # posteriors

pip.1ct.supp = pip.1ct.data$final$popPoints # support points

pip.1ct.stat=subset(pip.1ct.data$op$data,pip.1ct.data$op$data$pred.t
ype=="post"&pip.1ct.data$op$data$icen=="mean")

pip.1ct.stat.list=split(pip.1ct.stat,pip.1ct.stat$id) # list by
subject initials


pip.1ct.mse=NULL

pip.1ct.regr=data.frame(NA,NA,NA)


for (s in 1:length(pip.1ct.stat.list)) {

      pip.1ct.mse[[s]]=mean(pip.1ct.stat.list[[s]]$ds)

      pip.subj[[s]]=pip.1ct.stat.list[[s]][1,1]

      pip.1ct.regr[[s,1]]=lm(pip.1ct.stat.list[[s]]$pred~pip.1ct.stat
.list[[s]]$obs)$coefficients[[2]]

      pip.1ct.regr[[s,2]]=lm(pip.1ct.stat.list[[s]]$pred~pip.1ct.stat
.list[[s]]$obs)$coefficients[[1]]

      pip.1ct.regr[[s,3]]=cor(x=pip.1ct.stat.list[[s]]$pred,
      y=pip.1ct.stat.list[[s]]$obs, method="pearson")

}


pip.1ct.mse=unlist(pip.1ct.mse)

pip.subj=unlist(pip.subj)

pip.1ct.mse.wr=data.frame(pip.subj,pip.1ct.mse)

pip.1ct.regr=cbind(pip.subj, pip.1ct.regr)

colnames(pip.1ct.regr)=c("subject","slope","intercept","pearson r")


pip.2ct.data = PM_load(4)
```

```
pip.2ct.sum=pip.2ct.data$op$summary(pred.type =
"post",icen="median")

pip.2ct.post = pip.2ct.data$final$data$postMed # posteriors

pip.2ct.supp = pip.2ct.data$final$popPoints # support points

pip.2ct.stat=subset(pip.2ct.data$op$data,pip.2ct.data$op$data$pred.t
ype=="post"&pip.2ct.data$op$data$icen=="mean")

pip.2ct.stat.list=split(pip.2ct.stat,pip.2ct.stat$id) # list by
subject initials

pip.2ct.mse=NULL

pip.2ct.regr=data.frame(NA,NA,NA)



for (s in 1:length(pip.2ct.stat.list)) {

     pip.2ct.mse[[s]]=mean(pip.2ct.stat.list[[s]]$ds)

     pip.subj[[s]]=pip.2ct.stat.list[[s]][1,1]



     pip.2ct.regr[[s,1]]=lm(pip.2ct.stat.list[[s]]$pred~pip.2ct.stat
.list[[s]]$obs)$coefficients[[2]]


     pip.2ct.regr[[s,2]]=lm(pip.2ct.stat.list[[s]]$pred~pip.2ct.stat
.list[[s]]$obs)$coefficients[[1]]

     pip.2ct.regr[[s,3]]=cor(x=pip.2ct.stat.list[[s]]$pred,
     y=pip.2ct.stat.list[[s]]$obs, method="pearson")

  }


pip.2ct.mse=unlist(pip.2ct.mse)

pip.subj=unlist(pip.subj)

pip.2ct.mse.wr=data.frame(pip.subj,pip.2ct.mse)

pip.2ct.regr=cbind(pip.subj, pip.2ct.regr)

colnames(pip.2ct.regr)=c("subject","slope","intercept","pearson r")


pip.1ct.crcl.data = PM_load(3)

pip.1ct.crcl.sum=pip.1ct.crcl.data$op$summary(pred.type =
"post",icen="median")
```

```
pip.1ct.crcl.post = pip.1ct.crcl.data$final$data$postMed # posteriors

pip.1ct.crcl.supp = pip.1ct.crcl.data$final$popPoints # support points

pip.1ct.crcl.stat=subset(pip.1ct.crcl.data$op$data,pip.1ct.crcl.data$op$data$pred.type=="post"&pip.1ct.crcl.data$op$data$icen=="mean")

pip.1ct.crcl.stat.list=split(pip.1ct.crcl.stat,pip.1ct.crcl.stat$id) # list by subject initials

pip.1ct.crcl.mse=NULL

pip.1ct.crcl.regr=data.frame(NA,NA,NA)


for (s in 1:length(pip.1ct.crcl.stat.list)) {

	pip.1ct.crcl.mse[[s]]=mean(pip.1ct.crcl.stat.list[[s]]$ds)

	pip.subj[[s]]=pip.1ct.crcl.stat.list[[s]][1,1]

	pip.1ct.crcl.regr[[s,1]]=lm(pip.1ct.crcl.stat.list[[s]]$pred~pip.1ct.crcl.stat.list[[s]]$obs)$coefficients[[2]]

	pip.1ct.crcl.regr[[s,2]]=lm(pip.1ct.crcl.stat.list[[s]]$pred~pip.1ct.crcl.stat.list[[s]]$obs)$coefficients[[1]]


	pip.1ct.crcl.regr[[s,3]]=cor(x=pip.1ct.crcl.stat.list[[s]]$pred, y=pip.1ct.crcl.stat.list[[s]]$obs, method="pearson")

}


pip.1ct.crcl.mse=unlist(pip.1ct.crcl.mse)

pip.subj=unlist(pip.subj)

pip.1ct.crcl.mse.wr=data.frame(pip.subj,pip.1ct.crcl.mse)

pip.1ct.crcl.regr=cbind(pip.subj, pip.1ct.crcl.regr)

colnames(pip.1ct.crcl.regr)=c("subject","slope","intercept","pearson r")


pip.2ct.crcl.data = PM_load(4)

pip.2ct.crcl.sum=pip.2ct.crcl.data$op$summary(pred.type = "post",icen="median")

pip.2ct.crcl.post = pip.2ct.crcl.data$final$data$postMed # posteriors

pip.2ct.crcl.supp = pip.2ct.crcl.data$final$popPoints # support points
```

```
pip.2ct.crcl.stat=subset(pip.2ct.crcl.data$op$data,pip.2ct.crcl.data
$op$data$pred.type=="post"&pip.2ct.crcl.data$op$data$icen=="mean")

pip.2ct.crcl.stat.list=split(pip.2ct.crcl.stat,pip.2ct.crcl.stat$id)
# list by subject initials

pip.2ct.crcl.mse=NULL

pip.2ct.crcl.regr=data.frame(NA,NA,NA)


for (s in 1:length(pip.2ct.crcl.stat.list)) {

      pip.2ct.crcl.mse[[s]]=mean(pip.2ct.crcl.stat.list[[s]]$ds)

      pip.subj[[s]]=pip.2ct.crcl.stat.list[[s]][1,1]

      pip.2ct.crcl.regr[[s,1]]=lm(pip.2ct.crcl.stat.list[[s]]$pred~pi
p.2ct.crcl.stat.list[[s]]$obs)$coefficients[[2]]

      pip.2ct.crcl.regr[[s,2]]=lm(pip.2ct.crcl.stat.list[[s]]$pred~pi
p.2ct.crcl.stat.list[[s]]$obs)$coefficients[[1]]

pip.2ct.crcl.regr[[s,3]]=cor(x=pip.2ct.crcl.stat.list[[s]]$pred,
y=pip.2ct.crcl.stat.list[[s]]$obs, method="pearson")

}


pip.2ct.crcl.mse=unlist(pip.2ct.crcl.mse)

pip.subj=unlist(pip.subj)

pip.2ct.crcl.mse.wr=data.frame(pip.subj,pip.2ct.crcl.mse)

pip.2ct.crcl.regr=cbind(pip.subj, pip.2ct.crcl.regr)

colnames(pip.2ct.crcl.regr)=c("subject","slope","intercept","pearson
r")


comps=PM_compare(pip.1ct.data,pip.2ct.data,pip.1ct.crcl.data,pip.2ct
.crcl.data, icen="median")

comp.2ct=PM_compare(pip.2ct.data,pip.2ct.crcl.data, icen="median")


write.csv(pip.1ct.post,file="onecompt-posts.csv")

write.csv(pip.2ct.post,file="twocompt-posts.csv")

write.csv(pip.1ct.crcl.post,file="onecompt-crcl-posts.csv")

write.csv(pip.2ct.crcl.post,file="twocompt-crcl-posts.csv")


write.csv(pip.1ct.supp,file="onecompt-suppoints.csv")
```

```
write.csv(pip.2ct.supp,file="twocompt-suppoints.csv")

write.csv(pip.1ct.crcl.supp,file="onecompt-crcl-suppoints.csv")

write.csv(pip.2ct.crcl.supp,file="twocompt-crcl-suppoints.csv")


write.csv(pip.1ct.mse.wr,file="onecompt-mse.csv")

write.csv(pip.2ct.mse.wr,file="twocompt-mse.csv")

write.csv(pip.1ct.crcl.mse.wr,file="onecompt-crcl-mse.csv")

write.csv(pip.2ct.crcl.mse.wr,file="twocompt-crcl-mse.csv")
```

2. **Final model files used for nonparametric adaptive grid population pharmacokinetic modeling**

- **One compartment, no covariate**

```
#pri
Ke, 0.100000, 0.750000
V, 10.000000, 100.000000

#out
Y(1)=X(1)/V

#err
L=0.100000
0.255000,0.049873,-0.000361,0.000001
```

- **Two compartments, no covariate**

```
#pri
Ke, 0.100000, 1.700000
KPC, 0.050000, 5.000000
KCP, 0.050000, 5.000000
V, 5.000000, 35.000000

#out
Y(1)=X(1)/V

#err
L=0.100000
0.255000,0.049873,-0.000361,0.000001
```

- **One compartment, creatinine clearance covariate**

```
#pri
K, 0.001000, 0.006000
```

```
Ki, 0.005000, 0.100000
V, 15.000000, 80.000000

#cov
CRCL

#sec
Ke=K*CRCL+Ki

#out
Y(1)=X(1)/V

#err
L=0.100000
0.255000,0.049873,-0.000361,0.000001
```

- **Two compartments, creatinine clearance covariate**

```
#pri
K, 0.000500, 0.025000
Ki, 0.000000, 0.350000
V, 5.000000, 25.000000
KCP, 0.200000, 6.000000
KPC, 0.200000, 6.000000

#cov
CRCL

#sec
Ke=K*CRCL+Ki

#out
Y(1)=X(1)/V

#err
L=0.100000
0.255000,0.049873,-0.000361,0.000001
```

## 3. Supplementary Figure

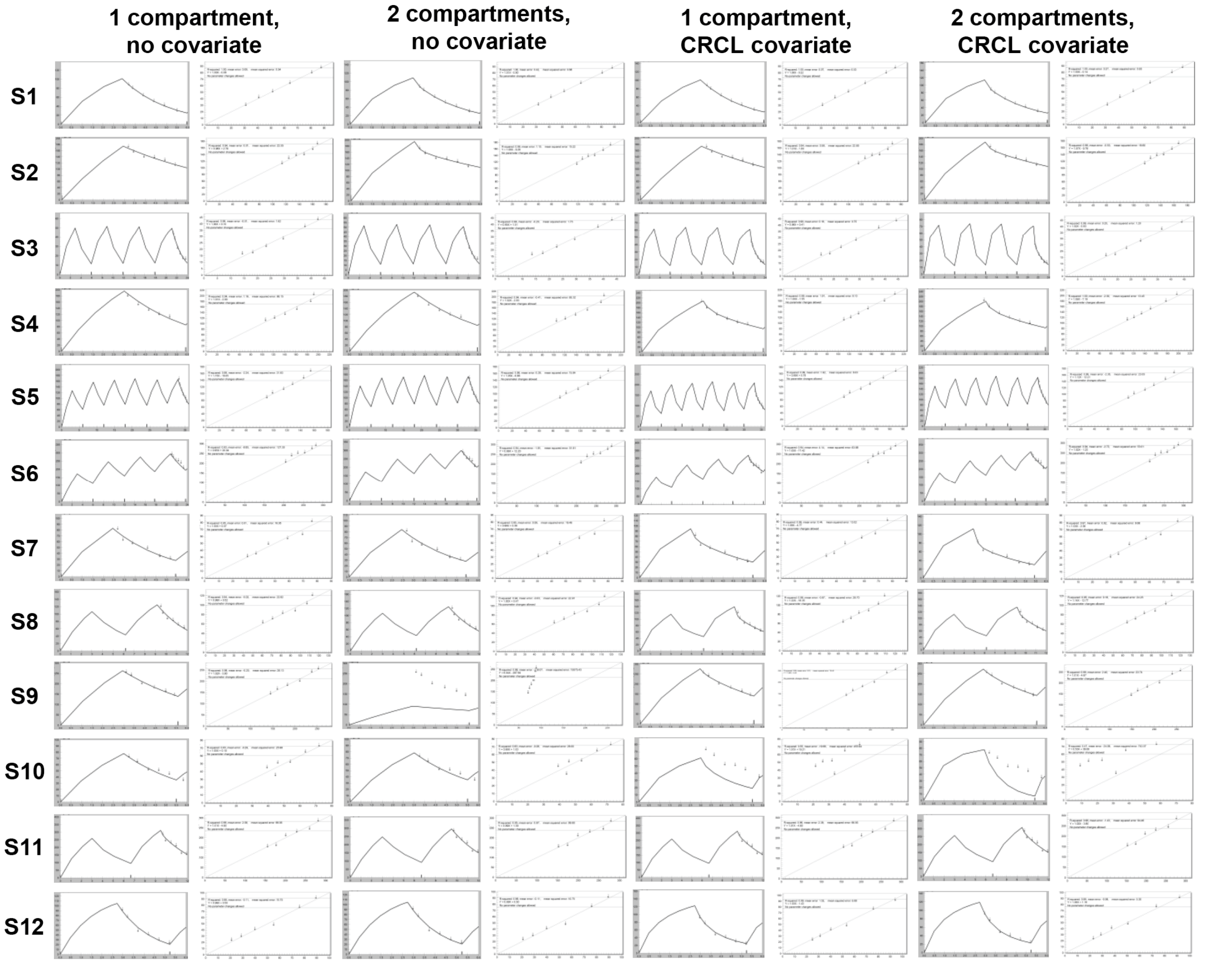


*Supplementary Figure S1. Individual concentration-time curves obtained when applying the best-performing quasi-models to the observed piperacillin concentrations. Each row shows the curves fitted, along with the correlation plots of the observed and predicted concentrations for a Subject (S1-S12). The horizontal and vertical axes of the fitted curve plots represent time (h) and piperacillin concentration (μmol/L), respectively. The origin (time=0 h) corresponds to the time of the initiation of treatment with piperacillin/tazobactam. The axes of the correlation plots are predicted and observed piperacillin concentrations (μmol/L). CRCL, creatinine clearance.*

4. **Supplementary tables.**

**Supplementary table S1. Coefficients of correlation between the values of the covariates tested, and the pharmacokinetic variables modeled. K, elimination rate constant. KCP, rate constant of the mass transfer from the central to the peripheral compartmetnt. KPC, rate constant of the mass transfer from the peripheral to the central compartment. V, Volume of distribution. $V_c$, volume of the central compartment.**

| Candidate covariate | Correlation coefficient | | | | | |
|---|---|---|---|---|---|---|
| | Single-compartment model | | Two-compartment model | | | |
| | K | V | K | KPC | KCP | $V_c$ |
| Serum sodium concentration | 0.0637 | 0.0664 | -0.0384 | 0.0895 | 0.0491 | 0.0281 |
| Serum potassium concentration | 0.5696 | 0.2672 | 0.3140 | -0.2399 | -0.2959 | 0.1741 |
| Serum glucose concentration | -0.2127 | 0.1090 | -0.0297 | -0.2184 | -0.2325 | -0.0199 |
| Serum creatinine concentration | -0.5330 | -0.3787 | -0.6435 | 0.7017 | 0.0507 | 0.2462 |
| Serum urea concentration | -0.6462 | -0.3014 | -0.6294 | 0.7233 | 0.2409 | 0.2153 |
| Serum total bilirubin concentration | -0.3739 | -0.5302 | -0.5920 | 0.7229 | 0.3923 | 0.0462 |
| White blood cell count | 0.2698 | 0.4800 | 0.7408 | -0.4525 | -0.0700 | -0.1677 |
| Platelet count | 0.3196 | 0.1956 | 0.3921 | -0.1077 | 0.1767 | -0.1307 |
| Hematocrit | 0.2567 | -0.0512 | 0.2007 | -0.0058 | 0.2053 | -0.1175 |
| Serum procalcitonin concentration | -0.1020 | -0.0974 | -0.0801 | -0.1923 | -0.1568 | -0.1217 |
| Serum C-reactive protein concentration | 0.0011 | 0.2121 | 0.2909 | -0.0819 | 0.2523 | -0.0362 |
| Serum interleukin-6 concentration | -0.1067 | -0.1398 | 0.0921 | -0.2615 | -0.1315 | -0.3063 |
| Serum cortisol concentration | -0.1809 | -0.2432 | -0.1134 | 0.1785 | 0.0597 | -0.1148 |
| Serum cortisone concentration | 0.3626 | -0.1841 | 0.0851 | 0.2288 | -0.0723 | 0.0044 |
| Serum 11-deoxycortisol concentration | -0.1059 | -0.0985 | -0.0451 | -0.2089 | -0.1494 | -0.1556 |
| Serum corticosterone concentration | -0.1096 | -0.1329 | 0.0391 | -0.1937 | -0.1030 | -0.2311 |
| Creatinine renal clearance | 0.8622 | 0.5002 | 0.8037 | -0.4033 | -0.0723 | 0.0693 |

**Supplementary tables S2-S13. Performance of the fully artificial population quasi-models generated for describing the pharmacokinetics of piperacillin. K, elimination rate constant (models without a covariate). KCP, rate constant of mass transfer from the central to the peripheral compartment. KI, nonrenal component of the elimination rate constant. KPC, coefficient of mass transfer from the peripheral to the central compartment. KS, renal component of the elimination rate constant (models with a covariate). $R^2$, determination coefficient. MAP Bayesian, maximum a posteriori probability Bayesian analysis. MSE, mean squared error. NPAG, nonparametric adaptive grid modeling. Pop-PK, population pharmacokinetic model. V, apparent volume of distribution. $V_c$, apparent volume of the central compartment.**

**Supplementary table S2: Performance of the quasi-models for Subject 1**

*Model #1: One compartment, no covariate*

| Model | K | V | $R^2$ | MSE |
|---|---|---|---|---|
| Pop-PK, NPAG | 0.461 | 41.950 | 0.999 | 0.554 |
| Pop-PK, MAP Bayesian | 0.458 | 42.152 | 0.999 | 0.539 |
| Quasi-model 1 | 0.437 | 45.560 | 0.999 | 0.579 |
| Quasi-model 2 | 0.430 | 46.200 | 0.999 | 4.436 |
| Quasi-model 3 | 0.478 | 41.672 | 0.999 | 0.629 |
| Quasi-model 4 | 0.463 | 42.377 | 0.999 | 0.780 |
| Quasi-model 5 | 0.476 | 40.436 | 0.999 | 1.203 |
| Quasi-model 6 | 0.446 | 42.587 | 0.999 | 0.840 |
| Quasi-model 7 | 0.444 | 43.791 | 0.999 | 1.349 |
| Quasi-model 8 | 0.473 | 41.556 | 0.999 | 0.681 |
| Quasi-model 9 | 0.450 | 42.835 | 0.999 | 0.598 |
| Quasi-model 10 | 0.450 | 43.185 | 0.999 | 0.721 |
| Quasi-model 11 | 0.439 | 43.036 | 0.999 | 2.624 |
| Quasi-model 12 | 0.471 | 39.900 | 0.999 | 5.343 |
| Quasi-model 13 | 0.462 | 41.661 | 0.999 | 1.299 |
| Quasi-model 14 | 0.482 | 39.654 | 0.999 | 2.334 |
| Quasi-model 15 | 0.438 | 42.484 | 0.999 | 0.538 |
| Quasi-model 16 | 0.462 | 42.226 | 0.999 | 0.696 |
| Quasi-model 17 | 0.438 | 44.408 | 0.999 | 1.312 |
| Quasi-model 18 | 0.441 | 44.299 | 0.999 | 1.296 |
| Quasi-model 19 | 0.419 | 45.958 | 0.999 | 1.970 |
| Quasi-model 20 | 0.459 | 42.126 | 0.999 | 0.566 |
| Quasi-model 21 | 0.429 | 45.285 | 0.999 | 1.586 |
| Quasi-model 22 | 0.439 | 44.775 | 0.999 | 2.497 |
| Quasi-model 23 | 0.448 | 43.615 | 0.999 | 1.141 |
| Quasi-model 24 | 0.445 | 42.984 | 0.999 | 1.285 |
| Quasi-model 25 | 0.451 | 43.652 | 0.999 | 1.864 |
| Quasi-model 26 | 0.441 | 43.012 | 0.999 | 2.318 |
| Quasi-model 27 | 0.497 | 38.990 | 0.998 | 3.270 |
| Quasi-model 28 | 0.458 | 42.133 | 0.999 | 0.594 |

| | | | | |
|---|---|---|---|---|
| Quasi-model 29 | 0.453 | 43.137 | 0.999 | 0.944 |
| Quasi-model 30 | 0.471 | 41.987 | 0.999 | 2.233 |

*Model #2: Two compartments, no covariate*

| **Model** | **K** | **KCP** | **KPC** | **V** | **$R^2$** | **MSE** |
|---|---|---|---|---|---|---|
| Pop-PK, NPAG | 1.287 | 4.663 | 2.982 | 13.492 | 0.999 | 0.508 |
| Pop-PK, MAP Bayesian | 2.982 | 1.287 | 4.663 | 13.492 | 0.999 | 0.508 |
| Quasi-model 1 | 0.928 | 3.693 | 3.278 | 18.801 | 0.998 | 8.441 |
| Quasi-model 2 | 0.965 | 3.283 | 3.835 | 20.179 | 0.998 | 1.116 |
| Quasi-model 3 | 0.634 | 0.929 | 2.234 | 28.268 | 0.998 | 2.196 |
| Quasi-model 4 | 0.826 | 2.359 | 3.622 | 19.802 | 0.998 | 3.422 |
| Quasi-model 5 | 0.779 | 2.867 | 3.502 | 21.162 | 0.999 | 5.415 |
| Quasi-model 6 | 1.101 | 3.884 | 3.367 | 15.593 | 0.999 | 11.075 |
| Quasi-model 7 | 1.270 | 3.169 | 2.301 | 13.139 | 0.998 | 0.891 |
| Quasi-model 8 | 0.725 | 2.218 | 2.803 | 25.357 | 0.998 | 2.915 |
| Quasi-model 9 | 0.982 | 1.600 | 2.575 | 17.846 | 0.998 | 2.249 |
| Quasi-model 10 | 0.672 | 1.349 | 3.873 | 26.125 | 0.998 | 3.204 |
| Quasi-model 11 | 0.756 | 1.103 | 3.846 | 20.776 | 0.998 | 9.276 |
| Quasi-model 12 | 0.797 | 1.659 | 2.907 | 24.150 | 0.997 | 2.566 |
| Quasi-model 13 | 0.837 | 2.691 | 4.135 | 20.470 | 0.999 | 12.678 |
| Quasi-model 14 | 0.756 | 2.018 | 2.804 | 23.538 | 0.998 | 0.812 |
| Quasi-model 15 | 0.843 | 1.948 | 3.852 | 23.771 | 0.998 | 14.606 |
| Quasi-model 16 | 0.974 | 2.797 | 3.093 | 18.894 | 0.998 | 5.690 |
| Quasi-model 17 | 0.960 | 3.284 | 3.846 | 23.299 | 0.998 | 1.610 |
| Quasi-model 18 | 0.877 | 1.410 | 2.688 | 19.595 | 0.998 | 2.152 |
| Quasi-model 19 | 0.777 | 1.331 | 2.455 | 25.639 | 0.998 | 1.183 |
| Quasi-model 20 | 0.783 | 2.855 | 3.493 | 21.156 | 0.999 | 6.011 |
| Quasi-model 21 | 1.245 | 3.760 | 2.483 | 11.444 | 0.998 | 4.241 |
| Quasi-model 22 | 1.266 | 3.181 | 2.393 | 12.972 | 0.998 | 0.974 |
| Quasi-model 23 | 0.724 | 1.199 | 2.273 | 25.383 | 0.998 | 1.807 |
| Quasi-model 24 | 0.641 | 1.354 | 2.719 | 28.709 | 0.998 | 0.867 |
| Quasi-model 25 | 0.668 | 1.244 | 3.865 | 26.230 | 0.998 | 3.043 |
| Quasi-model 26 | 0.941 | 3.804 | 3.779 | 18.436 | 0.999 | 6.105 |
| Quasi-model 27 | 1.115 | 2.495 | 2.517 | 16.340 | 0.998 | 11.435 |
| Quasi-model 28 | 0.881 | 2.411 | 2.628 | 18.750 | 0.998 | 2.530 |
| Quasi-model 29 | 0.631 | 1.347 | 3.245 | 28.384 | 0.999 | 0.679 |
| Quasi-model 30 | 1.077 | 2.833 | 2.600 | 15.362 | 0.998 | 21.561 |

*Model #3: One compartment, covariate: creatinine clearance*

| **Model** | **KS** | **KI** | **$V_c$** | **$R^2$** | **MSE** |
|---|---|---|---|---|---|
| Pop-PK, NPAG | 0.003 | 0.011 | 42.715 | 0.999 | 1.097 |
| Pop-PK, MAP Bayesian | 0.003 | 0.011 | 42.305 | 0.999 | 0.536 |
| Quasi-model 1 | 0.003 | 0.037 | 39.970 | 0.999 | 1.949 |
| Quasi-model 2 | 0.003 | 0.059 | 37.068 | 0.998 | 12.316 |

| | | | | | |
|---|---|---|---|---|---|
| Quasi-model 3 | 0.003 | 0.096 | 37.729 | 0.998 | 5.686 |
| Quasi-model 4 | 0.003 | 0.009 | 39.583 | 0.999 | 1.110 |
| Quasi-model 5 | 0.003 | 0.076 | 44.806 | 0.999 | 3.356 |
| Quasi-model 6 | 0.003 | 0.055 | 41.820 | 0.999 | 0.579 |
| Quasi-model 7 | 0.003 | 0.097 | 41.570 | 0.999 | 1.439 |
| Quasi-model 8 | 0.003 | 0.040 | 37.363 | 0.998 | 6.056 |
| Quasi-model 9 | 0.003 | 0.022 | 43.628 | 0.999 | 0.811 |
| Quasi-model 10 | 0.003 | 0.027 | 38.822 | 0.999 | 1.593 |
| Quasi-model 11 | 0.003 | 0.028 | 40.104 | 0.999 | 1.816 |
| Quasi-model 12 | 0.003 | 0.058 | 42.824 | 0.999 | 1.214 |
| Quasi-model 13 | 0.003 | 0.029 | 42.144 | 0.999 | 1.002 |
| Quasi-model 14 | 0.003 | 0.027 | 43.054 | 0.999 | 1.271 |
| Quasi-model 15 | 0.003 | 0.074 | 43.158 | 0.999 | 1.183 |
| Quasi-model 16 | 0.003 | 0.059 | 42.495 | 0.999 | 0.688 |
| Quasi-model 17 | 0.003 | 0.062 | 42.850 | 0.999 | 0.550 |
| Quasi-model 18 | 0.003 | 0.087 | 40.113 | 0.999 | 2.523 |
| Quasi-model 19 | 0.003 | 0.048 | 41.281 | 0.999 | 0.950 |
| Quasi-model 20 | 0.003 | 0.059 | 42.592 | 0.999 | 0.534 |
| Quasi-model 21 | 0.003 | 0.093 | 43.235 | 0.999 | 2.516 |
| Quasi-model 22 | 0.003 | 0.060 | 41.274 | 0.999 | 1.350 |
| Quasi-model 23 | 0.002 | 0.065 | 48.180 | 0.999 | 6.872 |
| Quasi-model 24 | 0.003 | 0.069 | 42.011 | 0.999 | 0.714 |
| Quasi-model 25 | 0.003 | 0.043 | 44.254 | 0.999 | 2.476 |
| Quasi-model 26 | 0.003 | 0.081 | 41.875 | 0.999 | 1.965 |
| Quasi-model 27 | 0.003 | 0.070 | 42.252 | 0.999 | 2.036 |
| Quasi-model 28 | 0.003 | 0.250 | 42.445 | 0.999 | 1.317 |
| Quasi-model 29 | 0.003 | 0.072 | 44.226 | 0.999 | 1.191 |
| Quasi-model 30 | 0.003 | 0.660 | 41.555 | 0.999 | 0.748 |

*Model #4: Two compartments, covariate: creatinine clearance*

| **Model** | **KS** | **KCP** | **KPC** | **KI** | **$V_c$** | **$R^2$** | **MSE** |
|---|---|---|---|---|---|---|---|
| Pop-PK, NPAG | 0.004 | 4.057 | 5.739 | 0.306 | 23.100 | 0.999 | 0.536 |
| Pop-PK, MAP Bayesian | 0.004 | 4.031 | 5.771 | 0.305 | 23.213 | 0.999 | 1.045 |
| Quasi-model 1 | 0.004 | 2.162 | 2.773 | 0.118 | 23.046 | 0.999 | 1.396 |
| Quasi-model 2 | 0.004 | 1.785 | 3.043 | 0.241 | 23.355 | 0.999 | 4.229 |
| Quasi-model 3 | 0.004 | 1.816 | 3.178 | 0.334 | 22.270 | 0.999 | 2.269 |
| Quasi-model 4 | 0.007 | 5.095 | 5.492 | 0.048 | 17.023 | 0.999 | 78.540 |
| Quasi-model 5 | 0.004 | 4.329 | 5.514 | 0.227 | 20.874 | 0.999 | 29.530 |
| Quasi-model 6 | 0.007 | 4.288 | 3.644 | 0.120 | 15.178 | 0.999 | 4.764 |
| Quasi-model 7 | 0.005 | 2.701 | 4.783 | 0.041 | 23.481 | 0.999 | 6.970 |
| Quasi-model 8 | 0.007 | 1.784 | 1.593 | 0.111 | 14.273 | 0.997 | 18.588 |
| Quasi-model 9 | 0.006 | 3.452 | 2.835 | 0.115 | 18.603 | 0.999 | 0.860 |
| Quasi-model 10 | 0.005 | 4.498 | 4.406 | 0.268 | 19.227 | 0.999 | 8.833 |
| Quasi-model 11 | 0.005 | 3.729 | 4.272 | 0.206 | 21.661 | 0.999 | 7.773 |
| Quasi-model 12 | 0.006 | 5.064 | 5.252 | 0.238 | 19.932 | 0.999 | 1.564 |
| Quasi-model 13 | 0.006 | 2.592 | 2.253 | 0.185 | 20.866 | 0.999 | 1.701 |

| | | | | | | | |
|---|---|---|---|---|---|---|---|
| Quasi-model 14 | 0.010 | 3.832 | 2.156 | 0.301 | 8.846 | 0.998 | 83.857 |
| Quasi-model 15 | 0.006 | 4.312 | 3.532 | 0.263 | 17.411 | 0.999 | 8.206 |
| Quasi-model 16 | 0.004 | 1.340 | 1.679 | 0.244 | 19.240 | 0.997 | 2.758 |
| Quasi-model 17 | 0.004 | 4.648 | 5.289 | 0.318 | 23.783 | 0.998 | 4.375 |
| Quasi-model 18 | 0.005 | 2.939 | 3.499 | 0.184 | 19.644 | 0.998 | 20.566 |
| Quasi-model 19 | 0.006 | 3.539 | 3.698 | 0.139 | 21.156 | 0.999 | 1.055 |
| Quasi-model 20 | 0.009 | 2.524 | 1.546 | 0.126 | 13.562 | 0.996 | 3.275 |
| Quasi-model 21 | 0.006 | 4.236 | 3.825 | 0.114 | 19.557 | 0.999 | 9.238 |
| Quasi-model 22 | 0.006 | 3.058 | 2.578 | 0.154 | 18.146 | 0.998 | 2.978 |
| Quasi-model 23 | 0.004 | 2.737 | 3.262 | 0.256 | 22.370 | 0.999 | 0.764 |
| Quasi-model 24 | 0.006 | 4.338 | 4.038 | 0.129 | 19.652 | 0.999 | 6.677 |
| Quasi-model 25 | 0.005 | 2.738 | 2.752 | 0.259 | 19.620 | 0.999 | 0.963 |
| Quasi-model 26 | 0.005 | 3.174 | 3.589 | 0.098 | 22.797 | 0.999 | 2.245 |
| Quasi-model 27 | 0.006 | 1.770 | 1.974 | 0.197 | 16.605 | 0.998 | 16.036 |
| Quasi-model 28 | 0.005 | 4.461 | 3.706 | 0.237 | 19.322 | 0.999 | 0.629 |
| Quasi-model 29 | 0.007 | 3.780 | 3.442 | 0.245 | 18.187 | 0.998 | 7.369 |
| Quasi-model 30 | 0.007 | 4.272 | 3.705 | 0.246 | 17.381 | 0.999 | 38.327 |

**Supplementary table S3: Performance of the quasi-models for Subject 2**

*Model #1: One compartment, no covariate*

| **Model** | **K** | **V** | **$R^2$** | **MSE** |
|---|---|---|---|---|
| Pop-PK, NPAG | 0.162 | 35.678 | 0.969 | 22.774 |
| Pop-PK, MAP | 0.165 | 35.289 | 0.938 | 22.597 |
| Quasi-model 1 | 0.144 | 33.199 | 0.938 | 22.597 |
| Quasi-model 2 | 0.169 | 36.902 | 0.938 | 47.374 |
| Quasi-model 3 | 0.154 | 36.368 | 0.938 | 49.029 |
| Quasi-model 4 | 0.163 | 35.190 | 0.938 | 25.111 |
| Quasi-model 5 | 0.161 | 34.959 | 0.938 | 23.008 |
| Quasi-model 6 | 0.173 | 33.185 | 0.938 | 31.119 |
| Quasi-model 7 | 0.156 | 38.177 | 0.938 | 36.506 |
| Quasi-model 8 | 0.174 | 37.787 | 0.938 | 26.485 |
| Quasi-model 9 | 0.171 | 35.846 | 0.939 | 79.781 |
| Quasi-model 10 | 0.162 | 35.520 | 0.938 | 23.738 |
| Quasi-model 11 | 0.180 | 32.406 | 0.938 | 41.174 |
| Quasi-model 12 | 0.156 | 36.063 | 0.939 | 156.513 |
| Quasi-model 13 | 0.169 | 38.058 | 0.938 | 27.033 |
| Quasi-model 14 | 0.158 | 35.181 | 0.938 | 116.942 |
| Quasi-model 15 | 0.175 | 37.496 | 0.938 | 30.457 |
| Quasi-model 16 | 0.161 | 37.216 | 0.938 | 52.015 |
| Quasi-model 17 | 0.157 | 35.854 | 0.938 | 38.286 |
| Quasi-model 18 | 0.163 | 34.373 | 0.938 | 40.993 |
| Quasi-model 19 | 0.164 | 38.261 | 0.938 | 27.527 |
| Quasi-model 20 | 0.178 | 34.271 | 0.938 | 128.264 |
| Quasi-model 21 | 0.158 | 39.229 | 0.939 | 35.384 |
| Quasi-model 22 | 0.168 | 32.539 | 0.938 | 77.567 |
| Quasi-model 23 | 0.169 | 36.699 | 0.938 | 45.675 |
| Quasi-model 24 | 0.168 | 36.511 | 0.938 | 22.552 |
| Quasi-model 25 | 0.165 | 35.682 | 0.938 | 25.347 |
| Quasi-model 26 | 0.058 | 37.036 | 0.938 | 26.730 |
| Quasi-model 27 | 0.160 | 33.939 | 0.938 | 44.798 |
| Quasi-model 28 | 0.154 | 38.564 | 0.938 | 30.223 |
| Quasi-model 29 | 0.168 | 34.714 | 0.938 | 27.238 |
| Quasi-model 30 | 0.166 | 35.611 | 0.938 | 24.184 |

*Model #2: Two compartments, no covariate*

| **Model** | **K** | **KCP** | **KPC** | **V** | **$R^2$** | **MSE** |
|---|---|---|---|---|---|---|
| Pop-PK, NPAG | 1.292 | 4.678 | 2.995 | 13.550 | 0.985 | 0.508 |
| Pop-PK, MAP Bayesian | 1.287 | 4.663 | 2.982 | 13.492 | 0.964 | 16.332 |
| Quasi-model 1 | 0.339 | 3.805 | 3.425 | 16.118 | 0.954 | 55.386 |
| Quasi-model 2 | 0.360 | 2.551 | 2.674 | 13.973 | 0.966 | 51.596 |
| Quasi-model 3 | 0.270 | 2.945 | 2.773 | 19.548 | 0.954 | 75.722 |
| Quasi-model 4 | 0.229 | 2.058 | 4.336 | 23.488 | 0.948 | 32.384 |
| Quasi-model 5 | 0.337 | 2.903 | 2.184 | 15.357 | 0.960 | 32.789 |

| | | | | | | |
|---|---|---|---|---|---|---|
| Quasi-model 6 | 0.303 | 2.524 | 1.768 | 16.315 | 0.961 | 16.941 |
| Quasi-model 7 | 0.319 | 3.210 | 2.144 | 15.931 | 0.956 | 25.224 |
| Quasi-model 8 | 0.273 | 2.403 | 2.208 | 16.587 | 0.960 | 20.884 |
| Quasi-model 9 | 0.291 | 2.751 | 3.204 | 16.472 | 0.962 | 227.949 |
| Quasi-model 10 | 0.326 | 3.525 | 2.353 | 14.151 | 0.958 | 43.970 |
| Quasi-model 11 | 0.312 | 3.174 | 2.233 | 14.482 | 0.966 | 45.001 |
| Quasi-model 12 | 0.270 | 2.661 | 2.721 | 17.956 | 0.956 | 19.998 |
| Quasi-model 13 | 0.252 | 3.148 | 3.159 | 20.226 | 0.958 | 47.325 |
| Quasi-model 14 | 0.348 | 3.659 | 2.675 | 15.586 | 0.964 | 15.219 |
| Quasi-model 15 | 0.252 | 3.536 | 4.394 | 21.920 | 0.952 | 30.874 |
| Quasi-model 16 | 0.335 | 4.506 | 3.137 | 16.008 | 0.956 | 80.099 |
| Quasi-model 17 | 0.353 | 3.527 | 1.827 | 14.174 | 0.964 | 65.168 |
| Quasi-model 18 | 0.283 | 2.78 | 2.180 | 15.229 | 0.958 | 56.027 |
| Quasi-model 19 | 0.247 | 3.262 | 4.415 | 21.589 | 0.948 | 44.119 |
| Quasi-model 20 | 0.346 | 2.903 | 2.182 | 15.345 | 0.959 | 20.913 |
| Quasi-model 21 | 0.303 | 2.525 | 1.955 | 16.590 | 0.960 | 26.090 |
| Quasi-model 22 | 0.261 | 3.188 | 2.829 | 17.767 | 0.953 | 29.699 |
| Quasi-model 23 | 0.274 | 2.571 | 3.082 | 16.561 | 0.956 | 20.516 |
| Quasi-model 24 | 0.286 | 2.756 | 3.237 | 17.924 | 0.961 | 265.71 |
| Quasi-model 25 | 0.272 | 2.433 | 3.237 | 22.292 | 0.959 | 318.759 |
| Quasi-model 26 | 0.286 | 3.012 | 1.755 | 14.321 | 0.962 | 62.512 |
| Quasi-model 27 | 0.285 | 2.69 | 3.076 | 17.929 | 0.951 | 25.184 |
| Quasi-model 28 | 0.286 | 3.102 | 3.304 | 18.316 | 0.958 | 65.133 |
| Quasi-model 29 | 0.247 | 3.262 | 4.415 | 21.589 | 0.948 | 44.119 |
| Quasi-model 30 | 0.272 | 3.014 | 3.235 | 17.634 | 0.951 | 89.276 |

*Model #3: One compartment, covariate: creatinine clearance*

| **Model** | **KS** | **KI** | **$V_c$** | **$R^2$** | **MSE** |
|---|---|---|---|---|---|
| Pop-PK, NPAG | 0.001 | 0.054 | 35.475 | 0.969 | 22.530 |
| Pop-PK, MAP Bayesian | 0.001 | 0.054 | 35.449 | 0.938 | 22.564 |
| Quasi-model 1 | 0.002 | 0.017 | 32.503 | 0.938 | 110.179 |
| Quasi-model 2 | 0.001 | 0.06 | 32.842 | 0.938 | 29.271 |
| Quasi-model 3 | 0.002 | 0.042 | 33.010 | 0.938 | 73.226 |
| Quasi-model 4 | 0.001 | 0.063 | 37.996 | 0.938 | 76.828 |
| Quasi-model 5 | 0.002 | 0.021 | 37.956 | 0.938 | 48.021 |
| Quasi-model 6 | 0.002 | 0.041 | 34.701 | 0.938 | 51.483 |
| Quasi-model 7 | 0.002 | 0.037 | 33.617 | 0.938 | 67.574 |
| Quasi-model 8 | 0.002 | 0.033 | 35.870 | 0.938 | 35.730 |
| Quasi-model 9 | 0.002 | 0.041 | 34.914 | 0.938 | 24.769 |
| Quasi-model 10 | 0.001 | 0.054 | 31.457 | 0.938 | 123.65 |
| Quasi-model 11 | 0.001 | 0.076 | 37.356 | 0.938 | 23.565 |
| Quasi-model 12 | 0.001 | 0.045 | 36.624 | 0.938 | 41.761 |
| Quasi-model 13 | 0.002 | 0.025 | 32.816 | 0.938 | 42.695 |
| Quasi-model 14 | 0.001 | 0.059 | 37.399 | 0.938 | 22.853 |
| Quasi-model 15 | 0.001 | 0.044 | 34.216 | 0.938 | 37.701 |
| Quasi-model 16 | 0.001 | 0.058 | 34.990 | 0.938 | 41.866 |

| | | | | | |
|---|---|---|---|---|---|
| Quasi-model 17 | 0.002 | 0.028 | 37.158 | 0.938 | 64.372 |
| Quasi-model 18 | 0.002 | 0.038 | 33.073 | 0.938 | 65.665 |
| Quasi-model 19 | 0.002 | 0.044 | 35.424 | 0.938 | 25.573 |
| Quasi-model 20 | 0.002 | 0.013 | 32.029 | 0.938 | 253.216 |
| Quasi-model 21 | 0.001 | 0.064 | 33.307 | 0.938 | 40.453 |
| Quasi-model 22 | 0.002 | 0.049 | 38.053 | 0.938 | 44.842 |
| Quasi-model 23 | 0.002 | 0.04 | 38.323 | 0.938 | 45.101 |
| Quasi-model 24 | 0.001 | 0.053 | 40.584 | 0.938 | 214.218 |
| Quasi-model 25 | 0.002 | 0.045 | 33.557 | 0.938 | 23.568 |
| Quasi-model 26 | 0.002 | 0.046 | 38.011 | 0.938 | 30.580 |
| Quasi-model 27 | 0.002 | 0.056 | 34.010 | 0.938 | 32.331 |
| Quasi-model 28 | 0.002 | 0.52 | 35.402 | 0.938 | 50.117 |
| Quasi-model 29 | 0.001 | 0.081 | 34.733 | 0.938 | 24.320 |
| Quasi-model 30 | 0.001 | 0.048 | 36.134 | 0.938 | 23.489 |

*Model #4: Two compartments, covariate: creatinine clearance*

| **Model** | **KS** | **KCP** | **KPC** | **KI** | **$V_c$** | **$R^2$** | **MSE** |
|---|---|---|---|---|---|---|---|
| Pop-PK, NPAG | 0.007 | 4.405 | 1.157 | 0.201 | 6.300 | 0.986 | 10.245 |
| Pop-PK, MAP Bayesian | 0.007 | 4.398 | 1.144 | 0.200 | 6.303 | 0.972 | 10.328 |
| Quasi-model 1 | 0.002 | 4.963 | 2.945 | 0.155 | 11.105 | 0.952 | 70.987 |
| Quasi-model 2 | 0.002 | 3.358 | 5.881 | 0.083 | 20.999 | 0.943 | 266.620 |
| Quasi-model 3 | 0.002 | 3.980 | 3.410 | 0.158 | 18.613 | 0.950 | 27.805 |
| Quasi-model 4 | 0.001 | 2.605 | 2.394 | 0.175 | 17.639 | 0.959 | 100.505 |
| Quasi-model 5 | 0.002 | 5.313 | 5.626 | 0.146 | 16.227 | 0.946 | 270.863 |
| Quasi-model 6 | 0.003 | 4.114 | 4.194 | 0.076 | 20.590 | 0.949 | 184.376 |
| Quasi-model 7 | 0.002 | 4.314 | 3.839 | 0.153 | 18.524 | 0.957 | 46.081 |
| Quasi-model 8 | 0.003 | 2.801 | 1.834 | 0.180 | 13.867 | 0.960 | 40.052 |
| Quasi-model 9 | 0.003 | 3.800 | 3.935 | 0.050 | 18.593 | 0.949 | 125.268 |
| Quasi-model 10 | 0.002 | 3.921 | 3.080 | 0.118 | 18.009 | 0.962 | 152.032 |
| Quasi-model 11 | 0.003 | 3.631 | 2.203 | 0.130 | 12.166 | 0.957 | 60.456 |
| Quasi-model 12 | 0.003 | 4.123 | 3.806 | 0.078 | 16.880 | 0.954 | 35.214 |
| Quasi-model 13 | 0.003 | 4.386 | 2.526 | 0.123 | 16.047 | 0.959 | 199.367 |
| Quasi-model 14 | 0.003 | 3.903 | 2.885 | 0.135 | 13.045 | 0.954 | 83.213 |
| Quasi-model 15 | 0.002 | 3.993 | 2.179 | 0.193 | 20.058 | 0.954 | 57.918 |
| Quasi-model 16 | 0.003 | 2.990 | 3.153 | 0.080 | 20.138 | 0.955 | 114.024 |
| Quasi-model 17 | 0.001 | 3.906 | 1.680 | 0.204 | 16.134 | 0.960 | 16.599 |
| Quasi-model 18 | 0.003 | 4.675 | 3.644 | 0.158 | 18.128 | 0.956 | 168.955 |
| Quasi-model 19 | 0.003 | 3.892 | 2.884 | 0.140 | 21.547 | 0.958 | 23.92 |
| Quasi-model 20 | 0.003 | 4.312 | 2.804 | 0.208 | 14.872 | 0.955 | 71.574 |
| Quasi-model 21 | 0.003 | 4.800 | 5.511 | 0.035 | 18.554 | 0.945 | 54.664 |
| Quasi-model 22 | 0.002 | 4.002 | 1.938 | 0.249 | 13.879 | 0.960 | 87.428 |
| Quasi-model 23 | 0.001 | 4.276 | 2.298 | 0.202 | 17.760 | 0.959 | 130.169 |
| Quasi-model 24 | 0.002 | 4.480 | 3.128 | 0.165 | 17.306 | 0.951 | 24.091 |
| Quasi-model 25 | 0.003 | 2.492 | 2.663 | 0.077 | 17.791 | 0.955 | 63.028 |
| Quasi-model 26 | 0.003 | 4.593 | 3.714 | 0.079 | 16.473 | 0.950 | 74.783 |
| Quasi-model 27 | 0.003 | 4.342 | 3.488 | 0.154 | 16.953 | 0.957 | 32.449 |

| | | | | | | | |
|---|---|---|---|---|---|---|---|
| Quasi-model 28 | 0.003 | 4.144 | 3.909 | 0.085 | 15.519 | 0.956 | 45.901 |
| Quasi-model 29 | 0.003 | 2.865 | 3.254 | 0.059 | 18.562 | 0.952 | 34.382 |
| Quasi-model 30 | 0.005 | 4.810 | 2.470 | 0.158 | 11.095 | 0.957 | 105.791 |

**Supplementary table S4: Performance of the quasi-models for Subject 3**

*Model #1: One compartment, no covariate*

| Model | K | V | $R^2$ | MSE |
|---|---|---|---|---|
| Pop-PK, NPAG | 0.474 | 91.450 | 0.987 | 2.317 |
| Pop-PK, MAP | 0.476 | 92.001 | 0.984 | 6.067 |
| Quasi-model 1 | 0.461 | 89.607 | 0.982 | 2.238 |
| Quasi-model 2 | 0.470 | 90.449 | 0.983 | 1.949 |
| Quasi-model 3 | 0.481 | 82.046 | 0.984 | 2.294 |
| Quasi-model 4 | 0.483 | 83.816 | 0.984 | 1.704 |
| Quasi-model 5 | 0.454 | 89.465 | 0.984 | 1.724 |
| Quasi-model 6 | 0.512 | 78.904 | 0.986 | 1.740 |
| Quasi-model 7 | 0.480 | 84.732 | 0.983 | 1.782 |
| Quasi-model 8 | 0.487 | 83.046 | 0.985 | 1.639 |
| Quasi-model 9 | 0.486 | 83.262 | 0.984 | 1.668 |
| Quasi-model 10 | 0.475 | 84.334 | 0.983 | 1.820 |
| Quasi-model 11 | 0.480 | 83.990 | 0.984 | 1.675 |
| Quasi-model 12 | 0.494 | 81.344 | 0.984 | 1.657 |
| Quasi-model 13 | 0.451 | 91.323 | 0.983 | 2.029 |
| Quasi-model 14 | 0.454 | 92.637 | 0.983 | 2.105 |
| Quasi-model 15 | 0.478 | 85.830 | 0.984 | 1.623 |
| Quasi-model 16 | 0.453 | 91.849 | 0.982 | 2.276 |
| Quasi-model 17 | 0.470 | 87.035 | 0.984 | 1.800 |
| Quasi-model 18 | 0.463 | 87.917 | 0.983 | 1.817 |
| Quasi-model 19 | 0.482 | 83.777 | 0.984 | 1.738 |
| Quasi-model 20 | 0.466 | 90.453 | 0.984 | 1.789 |
| Quasi-model 21 | 0.458 | 88.865 | 0.983 | 2.070 |
| Quasi-model 22 | 0.485 | 85.101 | 0.984 | 1.685 |
| Quasi-model 23 | 0.466 | 89.013 | 0.983 | 2.257 |
| Quasi-model 24 | 0.450 | 93.472 | 0.982 | 2.399 |
| Quasi-model 25 | 0.454 | 91.701 | 0.983 | 2.127 |
| Quasi-model 26 | 0.460 | 90.304 | 0.983 | 1.917 |
| Quasi-model 27 | 0.470 | 84.822 | 0.984 | 1.684 |
| Quasi-model 28 | 0.500 | 79.364 | 0.985 | 1.626 |
| Quasi-model 29 | 0.459 | 91.201 | 0.983 | 1.978 |
| Quasi-model 30 | 0.464 | 85.779 | 0.984 | 1.703 |

*Model #2: Two compartments, no covariate*

| Model | K | KCP | KPC | V | $R^2$ | MSE |
|---|---|---|---|---|---|---|
| Pop-PK, NPAG | 1.356 | 1.263 | 0.718 | 23.750 | 0.997 | 8.441 |
| Pop-PK, MAP Bayesian | 1.360 | 1.282 | 0.719 | 23.853 | 0.986 | 5.530 |

| | | | | | | |
|---|---|---|---|---|---|---|
| Quasi-model 1 | 1.236 | 3.145 | 1.622 | 27.474 | 0.991 | 1.800 |
| Quasi-model 2 | 1.204 | 2.626 | 2.008 | 31.969 | 0.987 | 3.801 |
| Quasi-model 3 | 1.140 | 1.987 | 1.886 | 31.165 | 0.987 | 1.783 |
| Quasi-model 4 | 1.253 | 1.882 | 1.419 | 23.788 | 0.992 | 6.220 |
| Quasi-model 5 | 1.267 | 2.483 | 1.539 | 28.146 | 0.989 | 1.479 |
| Quasi-model 6 | 1.376 | 2.932 | 1.852 | 26.581 | 0.989 | 1.715 |
| Quasi-model 7 | 1.205 | 3.117 | 2.102 | 33.801 | 0.985 | 6.858 |
| Quasi-model 8 | 1.079 | 2.460 | 2.104 | 31.573 | 0.988 | 3.686 |
| Quasi-model 9 | 1.062 | 1.878 | 1.738 | 32.105 | 0.988 | 2.131 |
| Quasi-model 10 | 1.434 | 3.538 | 1.952 | 27.547 | 0.986 | 3.412 |
| Quasi-model 11 | 1.185 | 1.349 | 0.817 | 24.079 | 0.992 | 0.920 |
| Quasi-model 12 | 1.316 | 1.237 | 0.983 | 24.066 | 0.992 | 2.307 |
| Quasi-model 13 | 1.407 | 3.838 | 2.064 | 27.801 | 0.989 | 1.553 |
| Quasi-model 14 | 1.174 | 3.736 | 2.259 | 29.337 | 0.988 | 7.468 |
| Quasi-model 15 | 1.210 | 2.005 | 1.447 | 29.446 | 0.990 | 1.922 |
| Quasi-model 16 | 1.240 | 2.151 | 1.383 | 25.735 | 0.991 | 2.419 |
| Quasi-model 17 | 1.237 | 1.817 | 1.775 | 27.038 | 0.990 | 1.682 |
| Quasi-model 18 | 1.137 | 1.982 | 1.887 | 31.240 | 0.987 | 1.540 |
| Quasi-model 19 | 1.302 | 2.332 | 1.803 | 27.301 | 0.989 | 2.430 |
| Quasi-model 20 | 1.268 | 2.799 | 1.933 | 28.163 | 0.987 | 1.705 |
| Quasi-model 21 | 1.313 | 2.934 | 1.860 | 26.586 | 0.989 | 1.412 |
| Quasi-model 22 | 1.190 | 3.123 | 2.113 | 33.777 | 0.985 | 5.164 |
| Quasi-model 23 | 1.259 | 2.764 | 2.074 | 28.195 | 0.987 | 1.824 |
| Quasi-model 24 | 1.384 | 3.608 | 2.268 | 27.569 | 0.986 | 1.812 |
| Quasi-model 25 | 1.435 | 3.520 | 1.940 | 26.895 | 0.988 | 2.440 |
| Quasi-model 26 | 1.248 | 3.419 | 2.008 | 29.059 | 0.988 | 1.217 |
| Quasi-model 27 | 1.197 | 3.862 | 1.870 | 28.681 | 0.993 | 0.755 |
| Quasi-model 28 | 1.332 | 3.287 | 2.187 | 27.393 | 0.987 | 2.134 |
| Quasi-model 29 | 1.430 | 2.473 | 1.369 | 26.282 | 0.99 | 2.702 |
| Quasi-model 30 | 1.216 | 2.612 | 2.201 | 29.627 | 0.989 | 1.163 |

*Model #3: One compartment, covariate: creatinine clearance*

| **Model** | **KS** | **KI** | **$V_c$** | **$R^2$** | **MSE** |
|---|---|---|---|---|---|
| Pop-PK, NPAG | 0.004 | 0.022 | 72.525 | 0.990 | 4.721 |
| Pop-PK, MAP Bayesian | 0.004 | 0.022 | 72.608 | 0.990 | 5.930 |
| Quasi-model 1 | 0.003 | 0.016 | 78.576 | 0.986 | 1.953 |
| Quasi-model 2 | 0.003 | 0.018 | 77.008 | 0.987 | 1.917 |
| Quasi-model 3 | 0.003 | 0.066 | 74.390 | 0.986 | 3.815 |
| Quasi-model 4 | 0.003 | 0.086 | 73.844 | 0.987 | 2.253 |
| Quasi-model 5 | 0.003 | 0.065 | 79.137 | 0.985 | 2.258 |
| Quasi-model 6 | 0.003 | 0.055 | 75.168 | 0.987 | 2.207 |
| Quasi-model 7 | 0.003 | 0.015 | 78.107 | 0.986 | 2.092 |
| Quasi-model 8 | 0.003 | 0.071 | 73.997 | 0.987 | 2.229 |
| Quasi-model 9 | 0.003 | 0.076 | 72.904 | 0.987 | 2.760 |
| Quasi-model 10 | 0.003 | 0.017 | 78.528 | 0.986 | 1.756 |
| Quasi-model 11 | 0.003 | 0.045 | 78.020 | 0.986 | 2.876 |

| | | | | | |
|---|---|---|---|---|---|
| Quasi-model 12 | 0.003 | 0.073 | 76.698 | 0.986 | 2.134 |
| Quasi-model 13 | 0.003 | 0.086 | 75.113 | 0.987 | 2.066 |
| Quasi-model 14 | 0.003 | 0.038 | 74.868 | 0.988 | 2.320 |
| Quasi-model 15 | 0.003 | 0.078 | 79.764 | 0.986 | 1.727 |
| Quasi-model 16 | 0.003 | 0.059 | 80.031 | 0.986 | 2.045 |
| Quasi-model 17 | 0.003 | 0.031 | 76.413 | 0.987 | 1.926 |
| Quasi-model 18 | 0.003 | 0.027 | 75.678 | 0.987 | 2.147 |
| Quasi-model 19 | 0.003 | 0.082 | 75.270 | 0.987 | 2.083 |
| Quasi-model 20 | 0.003 | 0.018 | 76.956 | 0.986 | 2.045 |
| Quasi-model 21 | 0.003 | 0.024 | 78.633 | 0.986 | 1.905 |
| Quasi-model 22 | 0.003 | 0.028 | 71.143 | 0.987 | 4.298 |
| Quasi-model 23 | 0.003 | 0.062 | 78.368 | 0.986 | 1.770 |
| Quasi-model 24 | 0.003 | 0.090 | 74.923 | 0.986 | 2.775 |
| Quasi-model 25 | 0.003 | 0.074 | 78.269 | 0.986 | 2.285 |
| Quasi-model 26 | 0.003 | 0.053 | 77.809 | 0.986 | 2.121 |
| Quasi-model 27 | 0.003 | 0.073 | 76.735 | 0.987 | 1.998 |
| Quasi-model 28 | 0.003 | 0.087 | 75.819 | 0.986 | 3.062 |
| Quasi-model 29 | 0.003 | 0.012 | 77.417 | 0.986 | 1.989 |
| Quasi-model 30 | 0.003 | 0.096 | 77.643 | 0.986 | 1.888 |

*Model #4: Two compartments, covariate: creatinine clearance*

| **Model** | **KS** | **KCP** | **KPC** | **KI** | **$V_c$** | **$R^2$** | **MSE** |
|---|---|---|---|---|---|---|---|
| Pop-PK, NPAG | 0.010 | 1.679 | 0.751 | 0.222 | 17.100 | 0.997 | 0.603 |
| Pop-PK, MAP Bayesian | 0.010 | 1.703 | 0.755 | 0.222 | 17.132 | 0.989 | 1.733 |
| Quasi-model 1 | 0.009 | 3.917 | 1.923 | 0.229 | 20.842 | 0.990 | 13.678 |
| Quasi-model 2 | 0.013 | 2.834 | 1.374 | 0.08 | 15.025 | 0.990 | 1.256 |
| Quasi-model 3 | 0.01 | 1.282 | 0.76 | 0.343 | 13.680 | 0.987 | 7.555 |
| Quasi-model 4 | 0.009 | 3.162 | 1.464 | 0.112 | 20.206 | 0.992 | 5.846 |
| Quasi-model 5 | 0.008 | 1.452 | 0.092 | 0.288 | 16.691 | 0.991 | 11.324 |
| Quasi-model 6 | 0.011 | 2.388 | 1.045 | 0.149 | 16.744 | 0.989 | 8.551 |
| Quasi-model 7 | 0.013 | 1.084 | 0.550 | 0.175 | 9.765 | 0.978 | 16.063 |
| Quasi-model 8 | 0.010 | 4.493 | 1.563 | 0.183 | 20.605 | 0.989 | 1.286 |
| Quasi-model 9 | 0.015 | 3.745 | 1.638 | 0.192 | 13.544 | 0.989 | 6.612 |
| Quasi-model 10 | 0.007 | 3.005 | 1.788 | 0.336 | 24.893 | 0.987 | 1.801 |
| Quasi-model 11 | 0.011 | 4.511 | 1.495 | 0.158 | 20.617 | 0.989 | 2.478 |
| Quasi-model 12 | 0.009 | 4.408 | 2.036 | 0.292 | 23.484 | 0.986 | 3.349 |
| Quasi-model 13 | 0.009 | 1.147 | 0.815 | 0.039 | 18.808 | 0.990 | 4.236 |
| Quasi-model 14 | 0.010 | 5.238 | 1.844 | 0.253 | 20.063 | 0.986 | 1.879 |
| Quasi-model 15 | 0.011 | 1.738 | 0.956 | 0.020 | 19.679 | 0.988 | 4.105 |
| Quasi-model 16 | 0.011 | 3.598 | 1.596 | 0.064 | 19.977 | 0.988 | 7.132 |
| Quasi-model 17 | 0.008 | 2.618 | 1.553 | 0.296 | 22.582 | 0.989 | 2.714 |
| Quasi-model 18 | 0.009 | 3.365 | 1.436 | 0.146 | 22.287 | 0.989 | 2.739 |
| Quasi-model 19 | 0.014 | 2.930 | 1.077 | 0.284 | 12.765 | 0.988 | 1.830 |
| Quasi-model 20 | 0.012 | 3.079 | 1.230 | 0.169 | 13.076 | 0.989 | 8.693 |
| Quasi-model 21 | 0.010 | 2.962 | 0.967 | 0.170 | 21.292 | 0.989 | 1.196 |
| Quasi-model 22 | 0.009 | 2.146 | 1.001 | 0.009 | 19.502 | 0.993 | 10.474 |

| | | | | | | | |
|---|---|---|---|---|---|---|---|
| Quasi-model 23 | 0.010 | 1.379 | 0.790 | 0.283 | 13.746 | 0.989 | 15.076 |
| Quasi-model 24 | 0.009 | 1.942 | 0.940 | 0.201 | 20.221 | 0.989 | 1.607 |
| Quasi-model 25 | 0.009 | 2.644 | 1.408 | 0.209 | 22.548 | 0.989 | 2.302 |
| Quasi-model 26 | 0.010 | 4.394 | 1.342 | 0.217 | 21.345 | 0.990 | 5.602 |
| Quasi-model 27 | 0.013 | 4.052 | 1.276 | 0.057 | 16.367 | 0.988 | 1.754 |
| Quasi-model 28 | 0.010 | 2.370 | 1.139 | 0.275 | 20.511 | 0.990 | 1.352 |
| Quasi-model 29 | 0.016 | 2.819 | 1.005 | 0.119 | 9.349 | 0.989 | 5.352 |
| Quasi-model 30 | 0.011 | 4.456 | 1.589 | 0.174 | 19.170 | 0.988 | 4.812 |

**Supplementary table S5: Performance of the quasi-models for Subject 4**

*Model #1: One compartment, no covariate*

| Model | K | V | $R^2$ | MSE |
|---|---|---|---|---|
| Pop-PK, NPAG | 0.272 | 26.650 | 0.984 | 36.060 |
| Pop-PK, MAP | 0.273 | 26.255 | 0.939 | 101.22 |
| Quasi-model 1 | 0.268 | 28.396 | 0.938 | 71.117 |
| Quasi-model 2 | 0.259 | 32.883 | 0.937 | 304.078 |
| Quasi-model 3 | 0.279 | 26.564 | 0.940 | 131.192 |
| Quasi-model 4 | 0.271 | 28.638 | 0.939 | 118.108 |
| Quasi-model 5 | 0.286 | 26.488 | 0.941 | 91.942 |
| Quasi-model 6 | 0.276 | 29.649 | 0.939 | 238.243 |
| Quasi-model 7 | 0.273 | 28.075 | 0.939 | 159.972 |
| Quasi-model 8 | 0.283 | 28.618 | 0.940 | 109.114 |
| Quasi-model 9 | 0.266 | 29.921 | 0.939 | 157.863 |
| Quasi-model 10 | 0.276 | 29.716 | 0.939 | 193.094 |
| Quasi-model 11 | 0.276 | 28.866 | 0.940 | 87.607 |
| Quasi-model 12 | 0.272 | 27.526 | 0.938 | 71.739 |
| Quasi-model 13 | 0.284 | 25.140 | 0.939 | 72.167 |
| Quasi-model 14 | 0.267 | 28.829 | 0.939 | 78.059 |
| Quasi-model 15 | 0.290 | 27.995 | 0.941 | 116.547 |
| Quasi-model 16 | 0.266 | 27.716 | 0.938 | 77.563 |
| Quasi-model 17 | 0.268 | 30.310 | 0.938 | 244.136 |
| Quasi-model 18 | 0.290 | 29.988 | 0.940 | 110.571 |
| Quasi-model 19 | 0.254 | 28.255 | 0.937 | 129.064 |
| Quasi-model 20 | 0.284 | 27.348 | 0.941 | 66.147 |
| Quasi-model 21 | 0.290 | 29.618 | 0.940 | 177.571 |
| Quasi-model 22 | 0.270 | 27.571 | 0.939 | 75.959 |
| Quasi-model 23 | 0.268 | 26.334 | 0.939 | 200.753 |
| Quasi-model 24 | 0.261 | 30.449 | 0.939 | 257.478 |
| Quasi-model 25 | 0.271 | 30.019 | 0.939 | 120.036 |
| Quasi-model 26 | 0.278 | 30.482 | 0.939 | 208.885 |
| Quasi-model 27 | 0.272 | 26.544 | 0.939 | 87.027 |
| Quasi-model 28 | 0.269 | 28.143 | 0.939 | 80.276 |
| Quasi-model 29 | 0.288 | 28.909 | 0.941 | 193.378 |
| Quasi-model 30 | 0.281 | 25.781 | 0.940 | 199.467 |

*Model #2: Two compartments, no covariate*

| Model | K | KCP | KPC | V | $R^2$ | MSE |
|---|---|---|---|---|---|---|
| Pop-PK, NPAG | 0.428 | 0.966 | 0.867 | 12.95 | 1.000 | 1.116 |
| Pop-PK, MAP Bayesian | 0.454 | 1.838 | 1.411 | 11.060 | 0.994 | 1434.563 |
| Quasi-model 1 | 0.521 | 3.837 | 2.134 | 9.934 | 0.997 | 177.345 |
| Quasi-model 2 | 0.469 | 2.156 | 1.362 | 10.780 | 0.994 | 33.283 |
| Quasi-model 3 | 0.573 | 3.975 | 2.836 | 11.512 | 0.987 | 288.82 |
| Quasi-model 4 | 0.482 | 2.787 | 2.545 | 15.576 | 0.992 | 42.556 |
| Quasi-model 5 | 0.497 | 1.594 | 1.562 | 14.041 | 0.993 | 20.523 |
| Quasi-model 6 | 0.442 | 1.379 | 1.564 | 14.871 | 0.989 | 22.169 |
| Quasi-model 7 | 0.416 | 1.212 | 1.104 | 13.371 | 0.994 | 70.384 |
| Quasi-model 8 | 0.591 | 2.951 | 1.275 | 13.543 | 0.998 | 360.764 |
| Quasi-model 9 | 0.374 | 1.341 | 1.455 | 13.190 | 0.992 | 629.852 |
| Quasi-model 10 | 0.472 | 2.282 | 2.646 | 16.211 | 0.990 | 398.170 |
| Quasi-model 11 | 0.385 | 1.923 | 2.316 | 16.546 | 0.978 | 334.610 |
| Quasi-model 12 | 0.700 | 2.982 | 1.082 | 10.238 | 0.998 | 36.567 |
| Quasi-model 13 | 0.483 | 2.925 | 3.223 | 13.230 | 0.986 | 70.965 |
| Quasi-model 14 | 0.357 | 2.141 | 1.575 | 14.042 | 0.995 | 42.072 |
| Quasi-model 15 | 0.57 | 2.738 | 1.736 | 12.755 | 0.992 | 20.030 |
| Quasi-model 16 | 0.521 | 2.088 | 1.551 | 12.001 | 0.996 | 47.791 |
| Quasi-model 17 | 0.567 | 3.100 | 1.545 | 10.167 | 0.995 | 9.096 |
| Quasi-model 18 | 0.530 | 3.985 | 2.831 | 11.489 | 0.992 | 138.501 |
| Quasi-model 19 | 0.481 | 2.564 | 2.569 | 15.567 | 0.987 | 23.439 |
| Quasi-model 20 | 0.498 | 1.582 | 1.556 | 14.054 | 0.994 | 23.695 |
| Quasi-model 21 | 0.439 | 1.380 | 1.565 | 14.875 | 0.989 | 20.764 |
| Quasi-model 22 | 0.416 | 1.206 | 1.116 | 13.266 | 0.994 | 79.353 |
| Quasi-model 23 | 0.591 | 3.264 | 1.288 | 13.546 | 0.998 | 373.423 |
| Quasi-model 24 | 0.404 | 1.506 | 1.685 | 14.967 | 0.990 | 268.341 |
| Quasi-model 25 | 0.471 | 2.295 | 2.639 | 13.750 | 0.989 | 157.614 |
| Quasi-model 26 | 0.430 | 1.715 | 2.192 | 15.047 | 0.985 | 86.466 |
| Quasi-model 27 | 0.425 | 3.098 | 3.314 | 14.478 | 0.977 | 65.843 |
| Quasi-model 28 | 0.238 | 0.572 | 0.488 | 19.163 | 0.990 | 72.704 |
| Quasi-model 29 | 0.404 | 2.113 | 2.171 | 11.990 | 0.989 | 81.648 |
| Quasi-model 30 | 0.488 | 1.768 | 1.039 | 12.580 | 0.997 | 128.298 |

*Model #3: One compartment, covariate: creatinine clearance*

| Model | KS | KI | $V_c$ | $R^2$ | MSE |
|---|---|---|---|---|---|
| Pop-PK, NPAG | 0.003 | 0.027 | 26.375 | 0.984 | 35.757 |
| Pop-PK, MAP Bayesian | 0.003 | 0.027 | 26.225 | 0.939 | 100.592 |
| Quasi-model 1 | 0.003 | 0.060 | 28.983 | 0.939 | 89.518 |
| Quasi-model 2 | 0.002 | 0.048 | 27.770 | 0.941 | 110.379 |
| Quasi-model 3 | 0.003 | 0.059 | 27.318 | 0.940 | 67.853 |
| Quasi-model 4 | 0.003 | 0.045 | 26.135 | 0.940 | 82.869 |
| Quasi-model 5 | 0.003 | 0.074 | 26.643 | 0.941 | 76.017 |
| Quasi-model 6 | 0.003 | 0.081 | 27.027 | 0.941 | 65.318 |
| Quasi-model 7 | 0.003 | 0.034 | 28.422 | 0.939 | 84.036 |

| | | | | | |
|---|---|---|---|---|---|
| Quasi-model 8 | 0.003 | 0.023 | 29.055 | 0.940 | 152.749 |
| Quasi-model 9 | 0.003 | 0.045 | 27.216 | 0.938 | 72.328 |
| Quasi-model 10 | 0.003 | 0.048 | 27.513 | 0.940 | 107.703 |
| Quasi-model 11 | 0.003 | 0.046 | 27.307 | 0.940 | 100.119 |
| Quasi-model 12 | 0.003 | 0.054 | 30.371 | 0.940 | 109.210 |
| Quasi-model 13 | 0.003 | 0.044 | 27.892 | 0.940 | 101.274 |
| Quasi-model 14 | 0.003 | 0.901 | 30.312 | 0.939 | 198.963 |
| Quasi-model 15 | 0.003 | 0.058 | 28.544 | 0.940 | 167.230 |
| Quasi-model 16 | 0.003 | 0.043 | 30.074 | 0.938 | 159.524 |
| Quasi-model 17 | 0.003 | 0.047 | 27.757 | 0.939 | 84.826 |
| Quasi-model 18 | 0.003 | 0.069 | 27.877 | 0.940 | 100.184 |
| Quasi-model 19 | 0.003 | 0.062 | 26.170 | 0.939 | 72.858 |
| Quasi-model 20 | 0.003 | 0.053 | 25.835 | 0.941 | 68.281 |
| Quasi-model 21 | 0.002 | 0.098 | 28.958 | 0.939 | 124.591 |
| Quasi-model 22 | 0.003 | 0.034 | 28.909 | 0.940 | 67.671 |
| Quasi-model 23 | 0.003 | 0.061 | 27.912 | 0.940 | 102.970 |
| Quasi-model 24 | 0.003 | 0.058 | 28.854 | 0.939 | 106.295 |
| Quasi-model 25 | 0.003 | 0.075 | 31.053 | 0.938 | 262.235 |
| Quasi-model 26 | 0.003 | 0.043 | 28.190 | 0.940 | 72.171 |
| Quasi-model 27 | 0.003 | 0.070 | 27.356 | 0.939 | 69.345 |
| Quasi-model 28 | 0.003 | 0.058 | 29.637 | 0.939 | 133.678 |
| Quasi-model 29 | 0.003 | 0.037 | 29.374 | 0.940 | 109.575 |
| Quasi-model 30 | 0.003 | 0.077 | 28.309 | 0.940 | 73.815 |

*Model #4: Two compartments, covariate: creatinine clearance*

| **Model** | **KS** | **KCP** | **KPC** | **KI** | **$V_c$** | **$R^2$** | **MSE** |
|---|---|---|---|---|---|---|---|
| Pop-PK, NPAG | 0.009 | 2.375 | 1.041 | 0.180 | 6.900 | 0.999 | 2.772 |
| Pop-PK, MAP Bayesian | 0.007 | 4.395 | 1.146 | 0.200 | 6.313 | 0.990 | 151.068 |
| Quasi-model 1 | 0.008 | 4.850 | 3.155 | 0.170 | 9.067 | 0.991 | 345.149 |
| Quasi-model 2 | 0.006 | 3.193 | 1.981 | 0.237 | 12.751 | 0.993 | 57.430 |
| Quasi-model 3 | 0.003 | 3.625 | 3.504 | 0.153 | 13.221 | 0.975 | 220.867 |
| Quasi-model 4 | 0.003 | 2.736 | 2.239 | 0.286 | 10.268 | 0.994 | 274.017 |
| Quasi-model 5 | 0.003 | 3.052 | 2.152 | 0.095 | 15.211 | 0.987 | 149.965 |
| Quasi-model 6 | 0.004 | 3.240 | 2.019 | 0.241 | 13.120 | 0.990 | 48.408 |
| Quasi-model 7 | 0.004 | 1.491 | 1.238 | 0.207 | 12.191 | 0.996 | 34.988 |
| Quasi-model 8 | 0.009 | 3.001 | 1.590 | 0.013 | 10.470 | 0.997 | 56.405 |
| Quasi-model 9 | 0.004 | 3.87 | 2.486 | 0.181 | 11.023 | 0.986 | 124.68 |
| Quasi-model 10 | 0.005 | 1.915 | 1.847 | 0.053 | 13.444 | 0.990 | 31.199 |
| Quasi-model 11 | 0.007 | 3.925 | 1.988 | 0.177 | 10.810 | 0.996 | 13.452 |
| Quasi-model 12 | 0.001 | 1.166 | 1.553 | 0.306 | 15.882 | 0.986 | 20.044 |
| Quasi-model 13 | 0.002 | 2.771 | 3.591 | 0.247 | 13.760 | 0.977 | 175.738 |
| Quasi-model 14 | 0.004 | 2.335 | 2.550 | 0.145 | 14.835 | 0.988 | 22.226 |
| Quasi-model 15 | 0.003 | 2.893 | 2.795 | 0.218 | 14.892 | 0.981 | 56.153 |
| Quasi-model 16 | 0.004 | 2.071 | 1.861 | 0.144 | 12.190 | 0.989 | 288.210 |
| Quasi-model 17 | 0.004 | 2.154 | 1.887 | 0.128 | 12.560 | 0.992 | 81.225 |
| Quasi-model 18 | 0.004 | 1.871 | 2.371 | 0.178 | 16.418 | 0.984 | 59.429 |

| | | | | | | | |
|---|---|---|---|---|---|---|---|
| Quasi-model 19 | 0.003 | 1.077 | 1.236 | 0.142 | 13.470 | 0.988 | 860.981 |
| Quasi-model 20 | 0.006 | 3.507 | 2.315 | 0.166 | 10.295 | 0.996 | 84.763 |
| Quasi-model 21 | 0.006 | 4.105 | 2.300 | 0.073 | 10.827 | 0.991 | 405.058 |
| Quasi-model 22 | 0.003 | 1.636 | 2.011 | 0.190 | 14.451 | 0.988 | 290.924 |
| Quasi-model 23 | 0.006 | 2.749 | 1.657 | 0.124 | 9.723 | 0.995 | 45.009 |
| Quasi-model 24 | 0.004 | 1.136 | 0.903 | 0.144 | 15.660 | 0.994 | 276.014 |
| Quasi-model 25 | 0.004 | 1.350 | 1.659 | 0.207 | 17.544 | 0.991 | 15.733 |
| Quasi-model 26 | 0.005 | 2.588 | 1.601 | 0.113 | 11.186 | 0.997 | 98.008 |
| Quasi-model 27 | 0.004 | 2.264 | 1.778 | 0.055 | 13.148 | 0.992 | 72.788 |
| Quasi-model 28 | 0.007 | 4.541 | 2.238 | 0.205 | 10.722 | 0.995 | 20.486 |
| Quasi-model 29 | 0.003 | 4.686 | 5.265 | 0.222 | 13.625 | 0.979 | 363.393 |
| Quasi-model 30 | 0.002 | 1.233 | 1.271 | 0.260 | 15.320 | 0.993 | 20.060 |

**Supplementary table S6: Performance of the quasi-models for Subject 5**

*Model #1: One compartment, no covariate*

| **Model** | **K** | **V** | **$R^2$** | **MSE** |
|---|---|---|---|---|
| Pop-PK, NPAG | 0.272 | 37.450 | 0.991 | 13.523 |
| Pop-PK, MAP | 0.276 | 37.093 | 0.982 | 13.386 |
| Quasi-model 1 | 0.241 | 43.447 | 0.98 | 31.829 |
| Quasi-model 2 | 0.259 | 40.664 | 0.981 | 27.391 |
| Quasi-model 3 | 0.261 | 41.444 | 0.981 | 42.235 |
| Quasi-model 4 | 0.278 | 38.216 | 0.982 | 28.478 |
| Quasi-model 5 | 0.284 | 34.600 | 0.982 | 19.969 |
| Quasi-model 6 | 0.329 | 31.392 | 0.984 | 39.915 |
| Quasi-model 7 | 0.324 | 31.560 | 0.984 | 22.123 |
| Quasi-model 8 | 0.289 | 35.061 | 0.982 | 15.065 |
| Quasi-model 9 | 0.247 | 43.113 | 0.981 | 14.631 |
| Quasi-model 10 | 0.269 | 38.894 | 0.982 | 18.737 |
| Quasi-model 11 | 0.256 | 38.088 | 0.98 | 50.876 |
| Quasi-model 12 | 0.266 | 38.270 | 0.981 | 14.362 |
| Quasi-model 13 | 0.305 | 34.536 | 0.983 | 29.327 |
| Quasi-model 14 | 0.265 | 38.734 | 0.981 | 15.066 |
| Quasi-model 15 | 0.229 | 45.445 | 0.979 | 33.753 |
| Quasi-model 16 | 0.312 | 32.763 | 0.984 | 23.709 |
| Quasi-model 17 | 0.301 | 32.309 | 0.983 | 64.636 |
| Quasi-model 18 | 0.267 | 38.728 | 0.981 | 15.632 |
| Quasi-model 19 | 0.288 | 35.495 | 0.982 | 14.644 |
| Quasi-model 20 | 0.749 | 99.849 | 0.979 | 67.223 |
| Quasi-model 21 | 0.262 | 39.629 | 0.981 | 16.207 |
| Quasi-model 22 | 0.301 | 32.341 | 0.983 | 66.004 |
| Quasi-model 23 | 0.288 | 35.491 | 0.982 | 14.49 |
| Quasi-model 24 | 0.284 | 36.943 | 0.982 | 20.465 |
| Quasi-model 25 | 0.243 | 41.753 | 0.98 | 24.581 |
| Quasi-model 26 | 0.254 | 39.814 | 0.981 | 17.548 |
| Quasi-model 27 | 0.324 | 31.506 | 0.984 | 27.805 |
| Quasi-model 28 | 0.315 | 32.308 | 0.983 | 19.241 |

| | | | | |
|---|---|---|---|---|
| Quasi-model 29 | 0.268 | 38.342 | 0.981 | 14.681 |
| Quasi-model 30 | 0.290 | 33.477 | 0.982 | 15.012 |

*Model #2: Two compartments, no covariate*

| **Model** | **K** | **KCP** | **KPC** | **V** | **$R^2$** | **MSE** |
|---|---|---|---|---|---|---|
| Pop-PK, NPAG | 0.748 | 1.906 | 1.065 | 12.05 | 0.998 | 2.196 |
| Pop-PK, MAP Bayesian | 0.745 | 1.883 | 1.085 | 12.039 | 0.995 | 3.557 |
| Quasi-model 1 | 0.669 | 3.668 | 2.097 | 15.963 | 0.989 | 30.629 |
| Quasi-model 2 | 0.699 | 3.868 | 2.486 | 14.215 | 0.987 | 20.513 |
| Quasi-model 3 | 0.541 | 1.030 | 0.887 | 17.357 | 0.993 | 6.822 |
| Quasi-model 4 | 0.528 | 2.781 | 3.245 | 16.751 | 0.989 | 23.12 |
| Quasi-model 5 | 0.588 | 3.138 | 2.501 | 17.421 | 0.986 | 32.766 |
| Quasi-model 6 | 0.444 | 2.315 | 4.044 | 22.292 | 0.99 | 23.108 |
| Quasi-model 7 | 0.486 | 1.759 | 2.140 | 19.214 | 0.987 | 27.305 |
| Quasi-model 8 | 0.441 | 2.503 | 3.603 | 22.292 | 0.987 | 12.049 |
| Quasi-model 9 | 0.449 | 3.103 | 4.774 | 24.328 | 0.983 | 112.095 |
| Quasi-model 10 | 0.641 | 2.819 | 1.540 | 15.505 | 0.993 | 55.511 |
| Quasi-model 11 | 0.404 | 2.184 | 3.410 | 24.322 | 0.986 | 22.510 |
| Quasi-model 12 | 0.741 | 2.478 | 1.496 | 12.377 | 0.995 | 8.027 |
| Quasi-model 13 | 0.741 | 3.689 | 2.290 | 13.420 | 0.990 | 39.204 |
| Quasi-model 14 | 0.339 | 0.756 | 2.208 | 29.366 | 0.987 | 21.405 |
| Quasi-model 15 | 0.492 | 2.588 | 3.288 | 18.893 | 0.987 | 16.103 |
| Quasi-model 16 | 0.712 | 3.658 | 2.099 | 14.718 | 0.989 | 23.919 |
| Quasi-model 17 | 0.688 | 3.977 | 2.495 | 14.270 | 0.985 | 15.565 |
| Quasi-model 18 | 0.508 | 1.743 | 2.501 | 19.080 | 0.992 | 5.975 |
| Quasi-model 19 | 0.574 | 2.920 | 3.252 | 19.226 | 0.989 | 14.742 |
| Quasi-model 20 | 0.587 | 3.130 | 2.497 | 17.429 | 0.986 | 37.192 |
| Quasi-model 21 | 0.441 | 2.313 | 4.044 | 22.297 | 0.990 | 21.123 |
| Quasi-model 22 | 0.485 | 1.748 | 2.143 | 19.283 | 0.987 | 15.108 |
| Quasi-model 23 | 0.44 | 2.503 | 3.589 | 22.298 | 0.987 | 12.468 |
| Quasi-model 24 | 0.451 | 3.621 | 3.768 | 24.286 | 0.983 | 138.902 |
| Quasi-model 25 | 0.639 | 2.826 | 1.569 | 15.546 | 0.992 | 58.993 |
| Quasi-model 26 | 0.430 | 0.675 | 0.779 | 21.874 | 0.993 | 5.292 |
| Quasi-model 27 | 0.479 | 3.377 | 3.128 | 21.261 | 0.985 | 83.252 |
| Quasi-model 28 | 0.477 | 2.018 | 1.840 | 21.648 | 0.990 | 56.173 |
| Quasi-model 29 | 0.545 | 4.333 | 4.057 | 19.156 | 0.984 | 52.471 |
| Quasi-model 30 | 0.506 | 2.158 | 2.106 | 20.049 | 0.988 | 37.232 |

*Model #3: One compartment, covariate: creatinine clearance*

| **Model** | **KS** | **KI** | **$V_c$** | **$R^2$** | **MSE** |
|---|---|---|---|---|---|
| Pop-PK, NPAG | 0.004 | 0.096 | 36.775 | 0.991 | 13.498 |
| Pop-PK, MAP Bayesian | 0.004 | 0.096 | 36.503 | 0.982 | 13.435 |
| Quasi-model 1 | 0.004 | 0.084 | 36.695 | 0.982 | 13.509 |
| Quasi-model 2 | 0.004 | 0.087 | 35.247 | 0.982 | 14.299 |

| | | | | | |
|---|---|---|---|---|---|
| Quasi-model 3 | 0.004 | 0.070 | 40.651 | 0.981 | 15.377 |
| Quasi-model 4 | 0.003 | 0.098 | 43.134 | 0.980 | 24.080 |
| Quasi-model 5 | 0.005 | 0.049 | 36.791 | 0.982 | 13.593 |
| Quasi-model 6 | 0.004 | 0.082 | 34.268 | 0.982 | 15.006 |
| Quasi-model 7 | 0.005 | 0.018 | 35.899 | 0.982 | 14.365 |
| Quasi-model 8 | 0.005 | 0.055 | 35.445 | 0.982 | 13.305 |
| Quasi-model 9 | 0.004 | 0.082 | 37.044 | 0.982 | 13.484 |
| Quasi-model 10 | 0.005 | 0.041 | 36.854 | 0.982 | 16.240 |
| Quasi-model 11 | 0.004 | 0.074 | 37.402 | 0.982 | 14.896 |
| Quasi-model 12 | 0.004 | 0.184 | 39.915 | 0.981 | 15.866 |
| Quasi-model 13 | 0.005 | 0.021 | 36.651 | 0.982 | 13.346 |
| Quasi-model 14 | 0.004 | 0.031 | 39.164 | 0.981 | 14.009 |
| Quasi-model 15 | 0.005 | 0.020 | 39.394 | 0.981 | 18.806 |
| Quasi-model 16 | 0.005 | 0.027 | 35.886 | 0.982 | 13.737 |
| Quasi-model 17 | 0.005 | 0.071 | 35.592 | 0.982 | 14.921 |
| Quasi-model 18 | 0.005 | 0.032 | 34.675 | 0.983 | 16.370 |
| Quasi-model 19 | 0.005 | 0.048 | 38.372 | 0.981 | 14.578 |
| Quasi-model 20 | 0.003 | 0.097 | 38.685 | 0.981 | 13.920 |
| Quasi-model 21 | 0.005 | 0.053 | 34.847 | 0.983 | 15.383 |
| Quasi-model 22 | 0.003 | 0.087 | 36.639 | 0.981 | 15.840 |
| Quasi-model 23 | 0.004 | 0.061 | 39.344 | 0.981 | 21.607 |
| Quasi-model 24 | 0.005 | 0.020 | 37.724 | 0.982 | 13.555 |
| Quasi-model 25 | 0.004 | 0.062 | 35.741 | 0.982 | 19.907 |
| Quasi-model 26 | 0.004 | 0.098 | 37.213 | 0.982 | 13.477 |
| Quasi-model 27 | 0.005 | 0.036 | 37.307 | 0.982 | 13.516 |
| Quasi-model 28 | 0.004 | 0.067 | 38.411 | 0.981 | 14.572 |
| Quasi-model 29 | 0.003 | 0.092 | 41.728 | 0.980 | 23.205 |
| Quasi-model 30 | 0.005 | 0.007 | 40.736 | 0.981 | 27.077 |

*Model #4: Two compartments, covariate: creatinine clearance*

| **Model** | **KS** | **KCP** | **KPC** | **KI** | **$V_c$** | **$R^2$** | **MSE** |
|---|---|---|---|---|---|---|---|
| Pop-PK, NPAG | 0.014 | 2.375 | 1.099 | 0.212 | 9.900 | 0.998 | 3.321 |
| Pop-PK, MAP Bayesian | 0.014 | 2.385 | 1.093 | 0.211 | 9.827 | 0.995 | 4.272 |
| Quasi-model 1 | 0.007 | 2.790 | 3.097 | 0.226 | 18.247 | 0.985 | 79.112 |
| Quasi-model 2 | 0.009 | 3.135 | 2.703 | 0.175 | 18.023 | 0.989 | 10.961 |
| Quasi-model 3 | 0.007 | 3.958 | 3.443 | 0.145 | 21.966 | 0.983 | 20.953 |
| Quasi-model 4 | 0.008 | 2.830 | 2.814 | 0.227 | 20.188 | 0.993 | 5.968 |
| Quasi-model 5 | 0.010 | 3.666 | 2.051 | 0.124 | 15.751 | 0.99 | 31.248 |
| Quasi-model 6 | 0.010 | 5.048 | 3.13 | 0.139 | 15.351 | 0.985 | 39.013 |
| Quasi-model 7 | 0.007 | 4.226 | 4.101 | 0.162 | 20.614 | 0.986 | 16.744 |
| Quasi-model 8 | 0.003 | 2.473 | 2.384 | 0.335 | 19.038 | 0.985 | 15.612 |
| Quasi-model 9 | 0.011 | 3.813 | 2.063 | 0.106 | 16.476 | 0.987 | 23.050 |
| Quasi-model 10 | 0.006 | 4.108 | 2.567 | 0.307 | 17.584 | 0.985 | 21.175 |
| Quasi-model 11 | 0.008 | 2.031 | 1.995 | 0.118 | 18.710 | 0.988 | 38.798 |
| Quasi-model 12 | 0.011 | 4.195 | 2.804 | 0.166 | 15.762 | 0.968 | 11.775 |
| Quasi-model 13 | 0.008 | 3.596 | 2.368 | 0.185 | 17.477 | 0.989 | 17.494 |

| | | | | | | | |
|---|---|---|---|---|---|---|---|
| Quasi-model 14 | 0.008 | 4.757 | 4.276 | 0.123 | 21.208 | 0.982 | 119.62 |
| Quasi-model 15 | 0.009 | 3.714 | 2.115 | 0.096 | 15.053 | 0.989 | 40.202 |
| Quasi-model 16 | 0.004 | 1.338 | 1.685 | 0.294 | 19.241 | 0.989 | 15.409 |
| Quasi-model 17 | 0.008 | 4.426 | 3.943 | 0.150 | 17.987 | 0.986 | 41.253 |
| Quasi-model 18 | 0.007 | 2.627 | 2.739 | 0.121 | 18.818 | 0.986 | 94.956 |
| Quasi-model 19 | 0.005 | 2.994 | 3.475 | 0.238 | 21.165 | 0.985 | 36.229 |
| Quasi-model 20 | 0.013 | 3.634 | 1.862 | 0.207 | 12.597 | 0.990 | 19.157 |
| Quasi-model 21 | 0.007 | 3.963 | 3.236 | 0.122 | 20.111 | 0.988 | 19.966 |
| Quasi-model 22 | 0.008 | 4.932 | 3.952 | 0.152 | 16.173 | 0.985 | 27.756 |
| Quasi-model 23 | 0.008 | 2.742 | 3.267 | 0.217 | 22.360 | 0.984 | 31.997 |
| Quasi-model 24 | 0.012 | 3.120 | 2.391 | 0.159 | 13.294 | 0.990 | 62.133 |
| Quasi-model 25 | 0.006 | 3.556 | 4.105 | 0.269 | 19.596 | 0.985 | 15.464 |
| Quasi-model 26 | 0.010 | 4.536 | 3.064 | 0.178 | 14.088 | 0.985 | 24.234 |
| Quasi-model 27 | 0.009 | 4.008 | 2.690 | 0.225 | 15.683 | 0.986 | 21.684 |
| Quasi-model 28 | 0.005 | 3.783 | 2.200 | 0.235 | 19.356 | 0.988 | 28.555 |
| Quasi-model 29 | 0.007 | 2.814 | 1.487 | 0.296 | 14.979 | 0.990 | 20.875 |
| Quasi-model 30 | 0.007 | 3.782 | 3.227 | 0.260 | 18.919 | 0.986 | 13.797 |

**Supplementary table S7: Performance of the quasi-models for Subject 6**

*Model #1: One compartment, no covariate*

| Model | K | V | $R^2$ | MSE |
|---|---|---|---|---|
| Pop-PK, NPAG | 0.129 | 38.35 | 0.968 | 45.317 |
| Pop-PK, MAP | 0.128 | 38.538 | 0.935 | 48.281 |
| Quasi-model 1 | 0.122 | 39.807 | 0.936 | 49.163 |
| Quasi-model 2 | 0.107 | 46.602 | 0.936 | 225.889 |
| Quasi-model 3 | 0.118 | 40.116 | 0.936 | 69.622 |
| Quasi-model 4 | 0.121 | 41.008 | 0.936 | 79.005 |
| Quasi-model 5 | 0.156 | 32.727 | 0.934 | 72.591 |
| Quasi-model 6 | 0.105 | 46.462 | 0.936 | 104.960 |
| Quasi-model 7 | 0.152 | 33.333 | 0.935 | 67.003 |
| Quasi-model 8 | 0.187 | 29.076 | 0.933 | 252.746 |
| Quasi-model 9 | 0.136 | 35.951 | 0.935 | 64.245 |
| Quasi-model 10 | 0.145 | 35.439 | 0.935 | 63.187 |
| Quasi-model 11 | 0.180 | 27.586 | 0.933 | 211.478 |
| Quasi-model 12 | 0.143 | 36.112 | 0.935 | 127.302 |
| Quasi-model 13 | 0.120 | 43.737 | 0.936 | 342.101 |
| Quasi-model 14 | 0.139 | 36.451 | 0.935 | 72.985 |
| Quasi-model 15 | 0.150 | 34.486 | 0.935 | 73.008 |
| Quasi-model 16 | 0.180 | 28.182 | 0.933 | 140.035 |
| Quasi-model 17 | 0.162 | 31.848 | 0.934 | 104.782 |
| Quasi-model 18 | 0.122 | 40.115 | 0.936 | 51.145 |
| Quasi-model 19 | 0.121 | 41.193 | 0.935 | 70.073 |
| Quasi-model 20 | 0.121 | 37.553 | 0.936 | 249.717 |
| Quasi-model 21 | 0.146 | 36.418 | 0.935 | 272.057 |
| Quasi-model 22 | 0.131 | 40.000 | 0.935 | 63.023 |

| | | | | |
|---|---|---|---|---|
| Quasi-model 23 | 0.130 | 37.117 | 0.936 | 50.216 |
| Quasi-model 24 | 0.178 | 28.729 | 0.934 | 118.164 |
| Quasi-model 25 | 0.106 | 46.060 | 0.936 | 99.051 |
| Quasi-model 26 | 0.157 | 31.458 | 0.934 | 153.182 |
| Quasi-model 27 | 0.143 | 34.687 | 0.935 | 67.861 |
| Quasi-model 28 | 0.128 | 38.951 | 0.935 | 64.144 |
| Quasi-model 29 | 0.139 | 37.007 | 0.935 | 88.566 |
| Quasi-model 30 | 0.130 | 36.494 | 0.935 | 209.178 |

*Model #2: Two compartments, no covariate*

| Model | K | KCP | KPC | V | $R^2$ | MSE |
|---|---|---|---|---|---|---|
| Pop-PK, NPAG | 0.204 | 2.500 | 4.777 | 24.050 | 0.968 | 45.728 |
| Pop-PK, MAP Bayesian | 0.204 | 2.504 | 4.792 | 24.042 | 0.936 | 50.773 |
| Quasi-model 1 | 0.319 | 2.205 | 2.288 | 16.035 | 0.937 | 158.016 |
| Quasi-model 2 | 0.470 | 2.164 | 1.283 | 9.843 | 0.935 | 94.458 |
| Quasi-model 3 | 0.254 | 2.963 | 4.266 | 20.309 | 0.937 | 52.552 |
| Quasi-model 4 | 0.222 | 2.066 | 4.341 | 23.369 | 0.936 | 118.055 |
| Quasi-model 5 | 0.211 | 3.053 | 2.863 | 21.910 | 0.938 | 75.657 |
| Quasi-model 6 | 0.160 | 1.053 | 4.175 | 31.169 | 0.937 | 47.349 |
| Quasi-model 7 | 0.318 | 3.697 | 2.256 | 15.966 | 0.938 | 60.548 |
| Quasi-model 8 | 0.233 | 2.392 | 3.842 | 21.913 | 0.937 | 81.124 |
| Quasi-model 9 | 0.331 | 2.346 | 3.207 | 14.015 | 0.937 | 173.002 |
| Quasi-model 10 | 0.193 | 2.331 | 0.547 | 27.863 | 0.931 | 51.615 |
| Quasi-model 11 | 0.159 | 0.498 | 3.013 | 31.341 | 0.937 | 54.124 |
| Quasi-model 12 | 0.271 | 2.665 | 2.715 | 17.946 | 0.937 | 46.657 |
| Quasi-model 13 | 0.251 | 3.215 | 3.161 | 20.182 | 0.936 | 57.740 |
| Quasi-model 14 | 0.345 | 3.680 | 2.689 | 15.664 | 0.938 | 187.323 |
| Quasi-model 15 | 0.346 | 1.844 | 1.740 | 13.854 | 0.936 | 158.478 |
| Quasi-model 16 | 0.334 | 4.509 | 3.126 | 14.924 | 0.937 | 95.392 |
| Quasi-model 17 | 0.351 | 3.524 | 1.799 | 14.176 | 0.937 | 82.048 |
| Quasi-model 18 | 0.463 | 4.270 | 1.810 | 10.148 | 0.939 | 60.628 |
| Quasi-model 19 | 0.252 | 3.788 | 4.421 | 19.438 | 0.936 | 47.772 |
| Quasi-model 20 | 0.211 | 3.073 | 2.858 | 21.918 | 0.938 | 67.938 |
| Quasi-model 21 | 0.158 | 1.054 | 4.177 | 31.162 | 0.937 | 48.389 |
| Quasi-model 22 | 0.223 | 2.695 | 3.755 | 21.738 | 0.937 | 48.326 |
| Quasi-model 23 | 0.233 | 2.392 | 3.855 | 21.912 | 0.937 | 83.004 |
| Quasi-model 24 | 0.331 | 2.345 | 3.227 | 14.015 | 0.937 | 172.843 |
| Quasi-model 25 | 0.146 | 0.380 | 0.507 | 29.240 | 0.923 | 56.522 |
| Quasi-model 26 | 0.236 | 1.734 | 1.612 | 19.422 | 0.938 | 63.684 |
| Quasi-model 27 | 0.287 | 2.691 | 3.072 | 17.894 | 0.937 | 126.656 |
| Quasi-model 28 | 0.213 | 1.097 | 2.150 | 23.413 | 0.936 | 73.842 |
| Quasi-model 29 | 0.194 | 2.527 | 3.025 | 25.023 | 0.938 | 73.125 |
| Quasi-model 30 | 0.346 | 4.603 | 3.014 | 14.055 | 0.936 | 172.926 |

*Model #3: One compartment, covariate: creatinine clearance*

| Model | KS | KI | $V_c$ | $R^2$ | MSE |
|---|---|---|---|---|---|
| Pop-PK, NPAG | 0.004 | 0.017 | 36.798 | 0.968 | 47.115 |
| Pop-PK, MAP Bayesian | 0.004 | 0.017 | 36.769 | 0.935 | 48.476 |
| Quasi-model 1 | 0.003 | 0.04 | 39.952 | 0.935 | 48.594 |
| Quasi-model 2 | 0.002 | 0.067 | 37.622 | 0.935 | 47.667 |
| Quasi-model 3 | 0.005 | 0.012 | 36.878 | 0.935 | 48.273 |
| Quasi-model 4 | 0.003 | 0.063 | 35.662 | 0.935 | 49.477 |
| Quasi-model 5 | 0.003 | 0.049 | 37.569 | 0.935 | 48.077 |
| Quasi-model 6 | 0.004 | 0.033 | 34.312 | 0.935 | 58.788 |
| Quasi-model 7 | 0.004 | 0.015 | 39.319 | 0.935 | 51.642 |
| Quasi-model 8 | 0.003 | 0.04 | 37.353 | 0.935 | 51.810 |
| Quasi-model 9 | 0.003 | 0.035 | 37.083 | 0.935 | 55.580 |
| Quasi-model 10 | 0.003 | 0.068 | 37.135 | 0.935 | 49.116 |
| Quasi-model 11 | 0.002 | 0.062 | 37.795 | 0.935 | 50.202 |
| Quasi-model 12 | 0.004 | 0.034 | 34.048 | 0.935 | 57.099 |
| Quasi-model 13 | 0.004 | 0.033 | 35.075 | 0.935 | 52.143 |
| Quasi-model 14 | 0.003 | 0.045 | 35.685 | 0.935 | 48.954 |
| Quasi-model 15 | 0.002 | 0.064 | 38.637 | 0.935 | 61.942 |
| Quasi-model 16 | 0.003 | 0.042 | 36.337 | 0.935 | 54.814 |
| Quasi-model 17 | 0.002 | 0.075 | 38.865 | 0.936 | 48.478 |
| Quasi-model 18 | 0.003 | 0.073 | 35.960 | 0.935 | 51.910 |
| Quasi-model 19 | 0.003 | 0.076 | 37.833 | 0.935 | 47.589 |
| Quasi-model 20 | 0.002 | 0.073 | 35.982 | 0.935 | 53.317 |
| Quasi-model 21 | 0.003 | 0.038 | 36.147 | 0.935 | 50.950 |
| Quasi-model 22 | 0.002 | 0.072 | 36.765 | 0.935 | 47.331 |
| Quasi-model 23 | 0.004 | 0.029 | 36.582 | 0.935 | 48.941 |
| Quasi-model 24 | 0.004 | 0.039 | 34.894 | 0.935 | 49.733 |
| Quasi-model 25 | 0.002 | 0.076 | 36.200 | 0.935 | 54.791 |
| Quasi-model 26 | 0.003 | 0.068 | 34.611 | 0.935 | 62.083 |
| Quasi-model 27 | 0.004 | 0.038 | 35.709 | 0.935 | 52.683 |
| Quasi-model 28 | 0.005 | 0.012 | 35.117 | 0.935 | 52.367 |
| Quasi-model 29 | 0.002 | 0.079 | 36.196 | 0.935 | 142.621 |
| Quasi-model 30 | 0.002 | 0.073 | 40.908 | 0.935 | 49.133 |

*Model #4: Two compartments, covariate: creatinine clearance*

| Model | KS | KCP | KPC | KI | $V_c$ | $R^2$ | MSE |
|---|---|---|---|---|---|---|---|
| Pop-PK, NPAG | 0.013 | 5.797 | 2.143 | 0.159 | 9.300 | 0.964 | 44.527 |
| Pop-PK, MAP Bayesian | 0.013 | 5.798 | 2.149 | 0.161 | 9.308 | 0.938 | 46.682 |
| Quasi-model 1 | 0.011 | 4.182 | 2.689 | 0.121 | 14.421 | 0.937 | 63.638 |
| Quasi-model 2 | 0.007 | 3.102 | 3.051 | 0.108 | 17.712 | 0.937 | 64.023 |
| Quasi-model 3 | 0.004 | 2.572 | 3.298 | 0.109 | 19.321 | 0.937 | 61.082 |
| Quasi-model 4 | 0.004 | 2.602 | 3.029 | 0.175 | 21.889 | 0.937 | 53.008 |
| Quasi-model 5 | 0.002 | 2.377 | 2.610 | 0.144 | 16.826 | 0.936 | 93.821 |
| Quasi-model 6 | 0.007 | 4.307 | 2.027 | 0.121 | 13.654 | 0.938 | 49.713 |
| Quasi-model 7 | 0.001 | 2.263 | 4.720 | 0.177 | 23.167 | 0.937 | 56.471 |

| | | | | | | | |
|---|---|---|---|---|---|---|---|
| Quasi-model 8 | 0.002 | 3.547 | 3.215 | 0.207 | 17.224 | 0.937 | 90.736 |
| Quasi-model 9 | 0.006 | 1.760 | 1.972 | 0.116 | 18.276 | 0.937 | 46.923 |
| Quasi-model 10 | 0.007 | 3.925 | 2.639 | 0.186 | 12.776 | 0.938 | 52.709 |
| Quasi-model 11 | 0.002 | 2.796 | 5.991 | 0.141 | 23.065 | 0.936 | 147.315 |
| Quasi-model 12 | 0.009 | 4.340 | 3.295 | 0.092 | 15.014 | 0.937 | 86.037 |
| Quasi-model 13 | 0.009 | 4.175 | 1.863 | 0.097 | 11.058 | 0.937 | 46.938 |
| Quasi-model 14 | 0.005 | 5.270 | 3.745 | 0.127 | 18.475 | 0.937 | 80.216 |
| Quasi-model 15 | 0.001 | 4.725 | 5.154 | 0.225 | 20.061 | 0.937 | 48.920 |
| Quasi-model 16 | 0.007 | 4.221 | 3.995 | 0.146 | 18.023 | 0.937 | 55.013 |
| Quasi-model 17 | 0.007 | 1.450 | 1.672 | 0.063 | 19.569 | 0.935 | 69.714 |
| Quasi-model 18 | 0.008 | 4.813 | 2.910 | 0.116 | 12.711 | 0.938 | 91.572 |
| Quasi-model 19 | 0.007 | 3.439 | 5.392 | 0.066 | 21.554 | 0.936 | 55.346 |
| Quasi-model 20 | 0.002 | 3.183 | 4.684 | 0.209 | 18.767 | 0.937 | 47.621 |
| Quasi-model 21 | 0.008 | 3.064 | 3.473 | 0.066 | 17.093 | 0.936 | 66.364 |
| Quasi-model 22 | 0.002 | 2.137 | 4.054 | 0.173 | 22.253 | 0.936 | 130.062 |
| Quasi-model 23 | 0.006 | 2.820 | 3.300 | 0.060 | 18.414 | 0.937 | 52.216 |
| Quasi-model 24 | 0.004 | 4.458 | 4.473 | 0.164 | 18.236 | 0.937 | 55.354 |
| Quasi-model 25 | 0.004 | 5.046 | 3.489 | 0.209 | 13.130 | 0.936 | 92.622 |
| Quasi-model 26 | 0.007 | 4.045 | 2.453 | 0.103 | 12.765 | 0.938 | 47.532 |
| Quasi-model 27 | 0.003 | 4.350 | 4.023 | 0.199 | 20.000 | 0.937 | 56.952 |
| Quasi-model 28 | 0.003 | 4.118 | 3.275 | 0.247 | 15.319 | 0.937 | 71.938 |
| Quasi-model 29 | 0.004 | 1.531 | 3.950 | 0.107 | 24.394 | 0.936 | 67.269 |
| Quasi-model 30 | 0.004 | 4.789 | 4.836 | 0.154 | 16.174 | 0.937 | 105.683 |

**Supplementary table S8: Performance of the quasi-models for Subject 7**

*Model #1: One compartment, no covariate*

| **Model** | **K** | **V** | **$R^2$** | **MSE** |
|---|---|---|---|---|
| Pop-PK, NPAG | 0.370 | 61.750 | 0.972 | 16.136 |
| Pop-PK, MAP | 0.368 | 61.296 | 0.946 | 16.061 |
| Quasi-model 1 | 0.369 | 61.281 | 0.946 | 16.346 |
| Quasi-model 2 | 0.375 | 60.379 | 0.946 | 16.102 |
| Quasi-model 3 | 0.361 | 63.788 | 0.946 | 16.660 |
| Quasi-model 4 | 0.349 | 65.248 | 0.945 | 17.792 |
| Quasi-model 5 | 0.366 | 63.268 | 0.946 | 16.121 |
| Quasi-model 6 | 0.385 | 60.202 | 0.946 | 16.500 |
| Quasi-model 7 | 0.344 | 65.096 | 0.945 | 16.925 |
| Quasi-model 8 | 0.378 | 61.597 | 0.946 | 17.490 |
| Quasi-model 9 | 0.358 | 63.897 | 0.946 | 16.271 |
| Quasi-model 10 | 0.372 | 60.699 | 0.946 | 16.244 |
| Quasi-model 11 | 0.368 | 61.286 | 0.946 | 16.094 |
| Quasi-model 12 | 0.377 | 61.576 | 0.946 | 16.598 |
| Quasi-model 13 | 0.369 | 62.252 | 0.946 | 16.352 |
| Quasi-model 14 | 0.360 | 61.233 | 0.946 | 16.857 |
| Quasi-model 15 | 0.422 | 55.203 | 0.946 | 15.952 |
| Quasi-model 16 | 0.360 | 63.403 | 0.946 | 16.463 |
| Quasi-model 17 | 0.353 | 61.129 | 0.945 | 19.735 |

| | | | | |
|---|---|---|---|---|
| Quasi-model 18 | 0.378 | 59.905 | 0.946 | 16.242 |
| Quasi-model 19 | 0.367 | 60.546 | 0.946 | 16.210 |
| Quasi-model 20 | 0.358 | 62.375 | 0.946 | 16.046 |
| Quasi-model 21 | 0.316 | 68.328 | 0.945 | 17.500 |
| Quasi-model 22 | 0.358 | 63.528 | 0.946 | 17.301 |
| Quasi-model 23 | 0.371 | 58.782 | 0.946 | 21.280 |
| Quasi-model 24 | 0.351 | 64.795 | 0.946 | 17.530 |
| Quasi-model 25 | 0.372 | 60.916 | 0.946 | 16.278 |
| Quasi-model 26 | 0.340 | 65.831 | 0.945 | 18.326 |
| Quasi-model 27 | 0.388 | 57.875 | 0.946 | 16.226 |
| Quasi-model 28 | 0.388 | 64.570 | 0.945 | 17.181 |
| Quasi-model 29 | 0.350 | 63.557 | 0.945 | 16.756 |
| Quasi-model 30 | 0.392 | 57.988 | 0.947 | 17.298 |

*Model #2: Two compartments, no covariate*

| Model | K | KCP | KPC | V | $R^2$ | MSE |
|---|---|---|---|---|---|---|
| Pop-PK, NPAG | 1.212 | 2.7 | 1.263 | 15.05 | 0.986 | 8.299 |
| Pop-PK, MAP Bayesian | 1.214 | 2.71 | 1.279 | 15.138 | 0.973 | 8.128 |
| Quasi-model 1 | 0.871 | 4.626 | 3.002 | 24.667 | 0.957 | 15.684 |
| Quasi-model 2 | 0.701 | 3.568 | 3.016 | 24.837 | 0.959 | 25.198 |
| Quasi-model 3 | 0.786 | 3.126 | 2.222 | 29.534 | 0.962 | 17.267 |
| Quasi-model 4 | 0.813 | 3.775 | 2.491 | 26.299 | 0.959 | 27.863 |
| Quasi-model 5 | 0.907 | 3.708 | 2.549 | 24.884 | 0.962 | 14.395 |
| Quasi-model 6 | 0.696 | 4.667 | 3.113 | 31.205 | 0.957 | 19.676 |
| Quasi-model 7 | 0.813 | 4.524 | 3.559 | 27.552 | 0.958 | 23.303 |
| Quasi-model 8 | 0.865 | 3.956 | 2.668 | 22.398 | 0.961 | 13.438 |
| Quasi-model 9 | 0.801 | 3.483 | 2.858 | 27.630 | 0.958 | 28.607 |
| Quasi-model 10 | 0.957 | 3.532 | 2.239 | 23.593 | 0.962 | 63.851 |
| Quasi-model 11 | 0.658 | 3.510 | 3.680 | 29.670 | 0.438 | 13.018 |
| Quasi-model 12 | 0.971 | 4.344 | 2.601 | 24.135 | 0.963 | 30.846 |
| Quasi-model 13 | 0.694 | 3.404 | 3.665 | 29.088 | 0.958 | 34.910 |
| Quasi-model 14 | 0.722 | 3.350 | 2.950 | 27.800 | 0.722 | 21.295 |
| Quasi-model 15 | 0.891 | 3.143 | 2.456 | 26.086 | 0.963 | 23.371 |
| Quasi-model 16 | 0.871 | 4.635 | 3.001 | 24.671 | 0.957 | 16.927 |
| Quasi-model 17 | 0.788 | 3.566 | 1.995 | 23.205 | 0.96 | 21.467 |
| Quasi-model 18 | 0.638 | 3.294 | 3.462 | 33.287 | 0.958 | 15.769 |
| Quasi-model 19 | 0.778 | 3.774 | 3.219 | 28.711 | 0.958 | 30.320 |
| Quasi-model 20 | 0.704 | 3.868 | 2.772 | 29.077 | 0.959 | 19.687 |
| Quasi-model 21 | 1.329 | 4.412 | 1.772 | 14.169 | 0.964 | 14.376 |
| Quasi-model 22 | 0.812 | 4.527 | 3.567 | 27.615 | 0.959 | 27.725 |
| Quasi-model 23 | 0.804 | 3.943 | 2.376 | 25.123 | 0.84 | 16.059 |
| Quasi-model 24 | 0.766 | 3.729 | 2.857 | 29.500 | 0.958 | 32.075 |
| Quasi-model 25 | 0.953 | 3.516 | 2.247 | 23.664 | 0.959 | 26.056 |
| Quasi-model 26 | 0.824 | 3.740 | 2.945 | 28.309 | 0.958 | 22.751 |
| Quasi-model 27 | 0.794 | 4.265 | 2.485 | 25.430 | 0.958 | 26.994 |
| Quasi-model 28 | 0.839 | 3.157 | 2.825 | 28.196 | 0.961 | 14.064 |

| | | | | | | |
|---|---|---|---|---|---|---|
| Quasi-model 29 | 0.79 | 4.117 | 3.230 | 27.730 | 0.955 | 32.327 |
| Quasi-model 30 | 0.753 | 4.558 | 3.802 | 29.217 | 0.959 | 17.839 |

*Model #3: One compartment, covariate: creatinine clearance*

| **Model** | **KS** | **KI** | **$V_c$** | **$R^2$** | **MSE** |
|---|---|---|---|---|---|
| Pop-PK, NPAG | 0.003 | 0.092 | 61.475 | 0.972 | 16.138 |
| Pop-PK, MAP Bayesian | 0.003 | 0.091 | 61.540 | 0.946 | 16.064 |
| Quasi-model 1 | 0.003 | 0.05 | 60.139 | 0.946 | 16.053 |
| Quasi-model 2 | 0.002 | 0.079 | 63.332 | 0.946 | 16.172 |
| Quasi-model 3 | 0.002 | 0.098 | 63.754 | 0.946 | 16.346 |
| Quasi-model 4 | 0.003 | 0.078 | 63.247 | 0.946 | 16.323 |
| Quasi-model 5 | 0.003 | 0.066 | 60.351 | 0.946 | 15.458 |
| Quasi-model 6 | 0.003 | 0.086 | 63.034 | 0.946 | 16.493 |
| Quasi-model 7 | 0.003 | 0.031 | 61.955 | 0.946 | 16.521 |
| Quasi-model 8 | 0.003 | 0.043 | 66.005 | 0.946 | 17.228 |
| Quasi-model 9 | 0.003 | 0.040 | 67.122 | 0.945 | 18.641 |
| Quasi-model 10 | 0.003 | 0.051 | 61.892 | 0.946 | 16.358 |
| Quasi-model 11 | 0.003 | 0.018 | 63.165 | 0.946 | 16.420 |
| Quasi-model 12 | 0.003 | 0.067 | 61.659 | 0.946 | 16.075 |
| Quasi-model 13 | 0.003 | 0.051 | 60.631 | 0.946 | 16.295 |
| Quasi-model 14 | 0.003 | 0.066 | 62.961 | 0.946 | 16.474 |
| Quasi-model 15 | 0.003 | 0.081 | 64.431 | 0.946 | 20.421 |
| Quasi-model 16 | 0.003 | 0.046 | 58.505 | 0.946 | 17.873 |
| Quasi-model 17 | 0.003 | 0.024 | 63.926 | 0.946 | 16.206 |
| Quasi-model 18 | 0.003 | 0.018 | 63.229 | 0.946 | 18.498 |
| Quasi-model 19 | 0.003 | 0.025 | 57.893 | 0.946 | 17.373 |
| Quasi-model 20 | 0.003 | 0.039 | 59.837 | 0.947 | 17.664 |
| Quasi-model 21 | 0.003 | 0.016 | 63.082 | 0.946 | 16.393 |
| Quasi-model 22 | 0.003 | 0.078 | 61.438 | 0.946 | 16.383 |
| Quasi-model 23 | 0.003 | 0.095 | 57.699 | 0.946 | 16.424 |
| Quasi-model 24 | 0.003 | 0.078 | 62.387 | 0.946 | 16.610 |
| Quasi-model 25 | 0.003 | 0.044 | 59.928 | 0.947 | 16.691 |
| Quasi-model 26 | 0.003 | 0.038 | 61.514 | 0.946 | 16.041 |
| Quasi-model 27 | 0.003 | 0.084 | 62.554 | 0.946 | 16.245 |
| Quasi-model 28 | 0.003 | 0.075 | 63.662 | 0.946 | 16.301 |
| Quasi-model 29 | 0.003 | 0.032 | 64.773 | 0.946 | 16.523 |
| Quasi-model 30 | 0.003 | 0.017 | 62.326 | 0.946 | 16.837 |

*Model #4: Two compartments, covariate: creatinine clearance*

| **Model** | **KS** | **KCP** | **KPC** | **KI** | **$V_c$** | **$R^2$** | **MSE** |
|---|---|---|---|---|---|---|---|
| Pop-PK, NPAG | 0.014 | 3.419 | 1.099 | 0.180 | 10.300 | 0.988 | 7.315 |
| Pop-PK, MAP Bayesian | 0.014 | 3.391 | 1.118 | 0.181 | 10.252 | 0.976 | 7.363 |
| Quasi-model 1 | 0.009 | 5.397 | 3.345 | 0.010 | 23.179 | 0.963 | 19.228 |
| Quasi-model 2 | 0.110 | 3.859 | 1.586 | 0.025 | 16.575 | 0.966 | 14.710 |

| | | | | | | | |
|---|---|---|---|---|---|---|---|
| Quasi-model 3 | 0.009 | 5.346 | 1.990 | 0.161 | 14.615 | 0.967 | 10.873 |
| Quasi-model 4 | 0.008 | 4.567 | 2.507 | 0.243 | 21.391 | 0.961 | 14.206 |
| Quasi-model 5 | 0.007 | 4.138 | 2.006 | 0.332 | 18.953 | 0.966 | 16.106 |
| Quasi-model 6 | 0.010 | 4.779 | 1.861 | 0.139 | 17.647 | 0.964 | 24.715 |
| Quasi-model 7 | 0.005 | 3.285 | 1.948 | 0.312 | 23.582 | 0.967 | 22.098 |
| Quasi-model 8 | 0.009 | 5.056 | 2.094 | 0.044 | 17.747 | 0.962 | 18.951 |
| Quasi-model 9 | 0.007 | 5.784 | 3.289 | 0.084 | 24.905 | 0.966 | 21.761 |
| Quasi-model 10 | 0.009 | 4.605 | 2.201 | 0.253 | 19.819 | 0.961 | 18.809 |
| Quasi-model 11 | 0.007 | 4.933 | 2.479 | 0.200 | 18.612 | 0.959 | 15.101 |
| Quasi-model 12 | 0.007 | 4.135 | 1.848 | 0.199 | 19.724 | 0.985 | 25.489 |
| Quasi-model 13 | 0.008 | 4.039 | 2.278 | 0.153 | 20.016 | 0.983 | 16.313 |
| Quasi-model 14 | 0.010 | 4.891 | 2.266 | 0.148 | 21.323 | 0.959 | 14.537 |
| Quasi-model 15 | 0.005 | 5.101 | 2.727 | 0.199 | 20.440 | 0.957 | 34.369 |
| Quasi-model 16 | 0.009 | 4.936 | 2.020 | 0.121 | 15.208 | 0.966 | 57.147 |
| Quasi-model 17 | 0.014 | 5.006 | 1.626 | 0.034 | 12.503 | 0.963 | 12.290 |
| Quasi-model 18 | 0.008 | 3.450 | 1.325 | 0.120 | 19.935 | 0.970 | 43.492 |
| Quasi-model 19 | 0.008 | 4.649 | 2.765 | 0.248 | 16.700 | 0.957 | 39.407 |
| Quasi-model 20 | 0.006 | 4.676 | 3.547 | 0.169 | 24.590 | 0.960 | 15.092 |
| Quasi-model 21 | 0.008 | 4.470 | 2.780 | 0.146 | 21.595 | 0.956 | 13.626 |
| Quasi-model 22 | 0.008 | 4.695 | 1.958 | 0.162 | 20.499 | 0.963 | 13.866 |
| Quasi-model 23 | 0.006 | 4.639 | 3.172 | 0.220 | 22.645 | 0.957 | 17.566 |
| Quasi-model 24 | 0.012 | 3.565 | 1.228 | 0.147 | 11.201 | 0.974 | 8.677 |
| Quasi-model 25 | 0.007 | 5.746 | 5.257 | 0.053 | 24.586 | 0.954 | 98.285 |
| Quasi-model 26 | 0.011 | 4.268 | 1.366 | 0.185 | 13.171 | 0.973 | 9.163 |
| Quasi-model 27 | 0.008 | 4.169 | 1.975 | 0.142 | 20.067 | 0.967 | 20.792 |
| Quasi-model 28 | 0.007 | 5.545 | 3.026 | 0.220 | 22.227 | 0.957 | 17.053 |
| Quasi-model 29 | 0.009 | 2.624 | 1.392 | 0.035 | 19.045 | 0.969 | 10.19 |
| Quasi-model 30 | 0.010 | 5.254 | 2.007 | 0.265 | 16.607 | 0.959 | 26.476 |

**Supplementary table S9: Performance of the quasi-models for Subject 8**

*Model #1: One compartment, no covariate*

| **Model** | **K** | **V** | **$R^2$** | **MSE** |
|---|---|---|---|---|
| Pop-PK, NPAG | 0.279 | 49.15 | 0.968 | 23.591 |
| Pop-PK, MAP | 0.281 | 49.737 | 0.939 | 23.026 |
| Quasi-model 1 | 0.274 | 48.002 | 0.939 | 36.485 |
| Quasi-model 2 | 0.289 | 47.524 | 0.939 | 23.979 |
| Quasi-model 3 | 0.255 | 53.331 | 0.938 | 24.342 |
| Quasi-model 4 | 0.306 | 45.752 | 0.940 | 23.322 |
| Quasi-model 5 | 0.292 | 47.310 | 0.939 | 23.093 |
| Quasi-model 6 | 0.245 | 59.564 | 0.939 | 31.349 |
| Quasi-model 7 | 0.282 | 46.373 | 0.940 | 50.850 |
| Quasi-model 8 | 0.302 | 46.050 | 0.940 | 24.441 |
| Quasi-model 9 | 0.252 | 53.850 | 0.936 | 28.967 |
| Quasi-model 10 | 0.249 | 53.539 | 0.936 | 29.828 |
| Quasi-model 11 | 0.273 | 52.046 | 0.938 | 23.426 |

| | | | | |
|---|---|---|---|---|
| Quasi-model 12 | 0.282 | 48.778 | 0.939 | 23.626 |
| Quasi-model 13 | 0.252 | 54.734 | 0.936 | 28.012 |
| Quasi-model 14 | 0.275 | 49.734 | 0.938 | 25.445 |
| Quasi-model 15 | 0.271 | 50.546 | 0.938 | 23.464 |
| Quasi-model 16 | 0.299 | 46.925 | 0.94 | 23.125 |
| Quasi-model 17 | 0.297 | 45.614 | 0.94 | 23.935 |
| Quasi-model 18 | 0.278 | 49.638 | 0.939 | 23.260 |
| Quasi-model 19 | 0.282 | 51.913 | 0.939 | 24.283 |
| Quasi-model 20 | 0.264 | 52.006 | 0.937 | 26.156 |
| Quasi-model 21 | 0.284 | 48.912 | 0.939 | 22.919 |
| Quasi-model 22 | 0.274 | 51.250 | 0.938 | 23.909 |
| Quasi-model 23 | 0.259 | 52.961 | 0.937 | 25.278 |
| Quasi-model 24 | 0.270 | 47.516 | 0.938 | 51.556 |
| Quasi-model 25 | 0.277 | 49.354 | 0.938 | 25.197 |
| Quasi-model 26 | 0.261 | 51.702 | 0.937 | 25.035 |
| Quasi-model 27 | 0.287 | 48.064 | 0.939 | 24.030 |
| Quasi-model 28 | 0.267 | 52.032 | 0.939 | 23.261 |
| Quasi-model 29 | 0.254 | 54.208 | 0.938 | 24.289 |
| Quasi-model 30 | 0.291 | 47.721 | 0.940 | 23.055 |

*Model #2: Two compartments, no covariate*

| **Model** | **K** | **KCP** | **KPC** | **V** | **$R^2$** | **MSE** |
|---|---|---|---|---|---|---|
| Pop-PK, NPAG | 1.644 | 1.906 | 1.065 | 5.75 | 0.994 | 4.138 |
| Pop-PK, MAP Bayesian | 1.646 | 2.466 | 0.546 | 5.648 | 0.99 | 3.877 |
| Quasi-model 1 | 0.667 | 3.817 | 1.579 | 19.524 | 0.954 | 78.711 |
| Quasi-model 2 | 0.960 | 4.135 | 1.282 | 12.431 | 0.954 | 56.993 |
| Quasi-model 3 | 0.682 | 3.171 | 1.629 | 17.746 | 0.961 | 34.189 |
| Quasi-model 4 | 0.662 | 4.542 | 1.749 | 18.348 | 0.957 | 44.053 |
| Quasi-model 5 | 0.634 | 2.161 | 1.332 | 18.559 | 0.965 | 33.378 |
| Quasi-model 6 | 0.673 | 3.598 | 2.128 | 23.817 | 0.952 | 82.834 |
| Quasi-model 7 | 0.830 | 3.058 | 1.995 | 13.991 | 0.959 | 32.429 |
| Quasi-model 8 | 0.450 | 4.192 | 2.897 | 30.497 | 0.952 | 39.076 |
| Quasi-model 9 | 0.466 | 4.875 | 4.555 | 30.118 | 0.947 | 68.414 |
| Quasi-model 10 | 0.798 | 4.470 | 2.142 | 16.781 | 0.956 | 53.407 |
| Quasi-model 11 | 0.814 | 4.255 | 1.896 | 14.647 | 0.952 | 32.713 |
| Quasi-model 12 | 0.581 | 4.251 | 2.022 | 23.983 | 0.951 | 63.422 |
| Quasi-model 13 | 0.588 | 4.106 | 2.417 | 22.673 | 0.941 | 94.283 |
| Quasi-model 14 | 0.668 | 4.279 | 2.546 | 21.652 | 0.941 | 105.975 |
| Quasi-model 15 | 0.578 | 4.371 | 2.863 | 25.943 | 0.941 | 142.364 |
| Quasi-model 16 | 0.670 | 3.692 | 1.568 | 19.455 | 0.959 | 95.754 |
| Quasi-model 17 | 0.735 | 4.145 | 1.289 | 20.222 | 0.953 | 65.380 |
| Quasi-model 18 | 0.680 | 3.184 | 1.639 | 17.671 | 0.951 | 34.981 |
| Quasi-model 19 | 0.661 | 4.396 | 1.750 | 18.345 | 0.957 | 58.888 |
| Quasi-model 20 | 0.544 | 2.903 | 2.423 | 26.090 | 0.946 | 53.361 |
| Quasi-model 21 | 0.473 | 3.947 | 3.624 | 28.903 | 0.942 | 91.278 |
| Quasi-model 22 | 0.827 | 3.574 | 1.763 | 14.090 | 0.957 | 28.703 |

| | | | | | | |
|---|---|---|---|---|---|---|
| Quasi-model 23 | 0.448 | 4.199 | 4.253 | 30.150 | 0.948 | 44.587 |
| Quasi-model 24 | 0.518 | 4.789 | 4.329 | 23.945 | 0.946 | 54.089 |
| Quasi-model 25 | 0.605 | 4.493 | 3.136 | 22.655 | 0.951 | 41.854 |
| Quasi-model 26 | 0.482 | 3.745 | 3.436 | 28.213 | 0.955 | 50.368 |
| Quasi-model 27 | 0.657 | 4.790 | 2.265 | 19.975 | 0.942 | 61.208 |
| Quasi-model 28 | 0.592 | 4.435 | 2.997 | 22.677 | 0.953 | 38.801 |
| Quasi-model 29 | 0.569 | 4.012 | 3.631 | 23.276 | 0.946 | 33.948 |
| Quasi-model 30 | 0.467 | 4.144 | 4.306 | 30.144 | 0.942 | 49.258 |

*Model #3: One compartment, covariate: creatinine clearance*

| **Model** | **KS** | **KI** | **$V_c$** | **$R^2$** | **MSE** |
|---|---|---|---|---|---|
| Pop-PK, NPAG | 0.003 | 0.023 | 47.824 | 0.969 | 23.766 |
| Pop-PK, MAP Bayesian | 0.004 | 0.096 | 36.560 | 0.946 | 56.184 |
| Quasi-model 1 | 0.003 | 0.051 | 50.347 | 0.938 | 23.675 |
| Quasi-model 2 | 0.003 | 0.029 | 47.086 | 0.939 | 22.991 |
| Quasi-model 3 | 0.003 | 0.081 | 52.840 | 0.938 | 23.528 |
| Quasi-model 4 | 0.003 | 0.037 | 49.473 | 0.939 | 24.631 |
| Quasi-model 5 | 0.003 | 0.033 | 55.297 | 0.937 | 26.292 |
| Quasi-model 6 | 0.002 | 0.096 | 50.015 | 0.939 | 23.023 |
| Quasi-model 7 | 0.003 | 0.011 | 48.808 | 0.939 | 22.997 |
| Quasi-model 8 | 0.003 | 0.053 | 50.539 | 0.938 | 27.035 |
| Quasi-model 9 | 0.003 | 0.035 | 50.548 | 0.938 | 23.467 |
| Quasi-model 10 | 0.003 | 0.079 | 48.320 | 0.939 | 22.909 |
| Quasi-model 11 | 0.003 | 0.026 | 52.617 | 0.938 | 25.563 |
| Quasi-model 12 | 0.003 | 0.072 | 64.592 | 0.94 | 23.044 |
| Quasi-model 13 | 0.003 | 0.048 | 49.439 | 0.939 | 23.163 |
| Quasi-model 14 | 0.003 | 0.036 | 52.636 | 0.938 | 23.976 |
| Quasi-model 15 | 0.003 | 0.092 | 49.705 | 0.94 | 24.756 |
| Quasi-model 16 | 0.003 | 0.086 | 47.594 | 0.939 | 23.137 |
| Quasi-model 17 | 0.003 | 0.044 | 51.398 | 0.939 | 23.258 |
| Quasi-model 18 | 0.003 | 0.026 | 48.505 | 0.939 | 23.666 |
| Quasi-model 19 | 0.003 | 0.027 | 52.786 | 0.939 | 23.818 |
| Quasi-model 20 | 0.003 | 0.064 | 51.879 | 0.937 | 26.801 |
| Quasi-model 21 | 0.003 | 0.057 | 51.173 | 0.939 | 23.316 |
| Quasi-model 22 | 0.003 | 0.059 | 48.812 | 0.939 | 23.019 |
| Quasi-model 23 | 0.003 | 0.022 | 49.142 | 0.939 | 22.911 |
| Quasi-model 24 | 0.003 | 0.072 | 53.461 | 0.939 | 23.186 |
| Quasi-model 25 | 0.003 | 0.075 | 45.627 | 0.940 | 23.351 |
| Quasi-model 26 | 0.003 | 0.055 | 50.046 | 0.939 | 26.182 |
| Quasi-model 27 | 0.003 | 0.060 | 48.366 | 0.939 | 22.987 |
| Quasi-model 28 | 0.003 | 0.051 | 47.495 | 0.940 | 23.064 |
| Quasi-model 29 | 0.003 | 0.056 | 48.691 | 0.939 | 25.840 |
| Quasi-model 30 | 0.003 | 0.009 | 51.691 | 0.937 | 25.368 |

*Model #4: Two compartments, covariate: creatinine clearance*

| Model | KS | KCP | KPC | KI | $V_c$ | $R^2$ | MSE |
|---|---|---|---|---|---|---|---|
| Pop-PK, NPAG | 0.015 | 2.375 | 0.577 | 0.079 | 6.300 | 0.994 | 4.431 |
| Pop-PK, MAP Bayesian | 0.014 | 3.391 | 1.118 | 0.181 | 10.251 | 0.967 | 86.877 |
| Quasi-model 1 | 0.012 | 4.489 | 1.365 | 0.221 | 13.073 | 0.953 | 55.227 |
| Quasi-model 2 | 0.008 | 3.86 | 1.594 | 0.104 | 16.573 | 0.953 | 52.373 |
| Quasi-model 3 | 0.008 | 4.344 | 1.778 | 0.238 | 14.625 | 0.958 | 33.516 |
| Quasi-model 4 | 0.007 | 4.571 | 3.425 | 0.069 | 21.918 | 0.953 | 34.163 |
| Quasi-model 5 | 0.008 | 4.681 | 2.036 | 0.171 | 15.542 | 0.946 | 78.297 |
| Quasi-model 6 | 0.005 | 3.621 | 2.187 | 0.199 | 22.434 | 0.95 | 36.385 |
| Quasi-model 7 | 0.008 | 4.166 | 1.526 | 0.265 | 18.064 | 0.951 | 173.209 |
| Quasi-model 8 | 0.006 | 5.101 | 2.686 | 0.105 | 19.324 | 0.941 | 42.983 |
| Quasi-model 9 | 0.008 | 4.558 | 1.602 | 0.084 | 16.002 | 0.946 | 85.586 |
| Quasi-model 10 | 0.011 | 4.582 | 1.536 | 0.240 | 15.635 | 0.954 | 69.444 |
| Quasi-model 11 | 0.007 | 4.871 | 2.487 | 0.199 | 18.661 | 0.948 | 30.265 |
| Quasi-model 12 | 0.007 | 3.505 | 1.844 | 0.189 | 19.765 | 0.954 | 146.328 |
| Quasi-model 13 | 0.010 | 4.325 | 1.605 | 0.086 | 15.354 | 0.95 | 102.499 |
| Quasi-model 14 | 0.006 | 4.084 | 2.010 | 0.192 | 19.585 | 0.945 | 57.984 |
| Quasi-model 15 | 0.005 | 4.484 | 2.729 | 0.162 | 20.426 | 0.942 | 73.442 |
| Quasi-model 16 | 0.008 | 2.400 | 1.208 | 0.145 | 17.496 | 0.967 | 27.066 |
| Quasi-model 17 | 0.011 | 4.953 | 1.095 | 0.040 | 14.573 | 0.946 | 39.911 |
| Quasi-model 18 | 0.005 | 4.854 | 2.230 | 0.143 | 21.081 | 0.950 | 56.700 |
| Quasi-model 19 | 0.009 | 4.222 | 1.792 | 0.114 | 16.699 | 0.951 | 60.435 |
| Quasi-model 20 | 0.006 | 4.595 | 1.969 | 0.169 | 18.049 | 0.946 | 79.587 |
| Quasi-model 21 | 0.005 | 3.568 | 1.866 | 0.274 | 19.966 | 0.951 | 60.527 |
| Quasi-model 22 | 0.006 | 4.797 | 2.980 | 0.300 | 20.256 | 0.942 | 39.818 |
| Quasi-model 23 | 0.006 | 5.387 | 3.581 | 0.157 | 22.467 | 0.943 | 34.263 |
| Quasi-model 24 | 0.013 | 3.546 | 1.218 | 0.146 | 11.164 | 0.973 | 49.977 |
| Quasi-model 25 | 0.005 | 4.991 | 4.043 | 0.214 | 22.371 | 0.950 | 24.227 |
| Quasi-model 26 | 0.010 | 3.688 | 1.365 | 0.191 | 12.801 | 0.962 | 69.528 |
| Quasi-model 27 | 0.007 | 4.030 | 2.219 | 0.151 | 18.741 | 0.957 | 45.340 |
| Quasi-model 28 | 0.006 | 4.800 | 1.945 | 0.282 | 17.921 | 0.944 | 36.518 |
| Quasi-model 29 | 0.009 | 5.589 | 1.610 | 0.036 | 18.753 | 0.951 | 93.079 |
| Quasi-model 30 | 0.008 | 2.916 | 0.883 | 0.176 | 14.019 | 0.971 | 61.228 |

**Supplementary table S10: Performance of the quasi-models for Subject 9**

*Model #1: One compartment, no covariate*

| Model | K | V | $R^2$ | MSE |
|---|---|---|---|---|
| Pop-PK, NPAG | 0.240 | 21.250 | 0.992 | 27.606 |
| Pop-PK, MAP | 0.240 | 21.050 | 0.984 | 26.543 |
| Quasi-model 1 | 0.250 | 20.239 | 0.984 | 92.895 |
| Quasi-model 2 | 0.262 | 17.743 | 0.985 | 156.758 |
| Quasi-model 3 | 0.226 | 22.622 | 0.984 | 29.618 |
| Quasi-model 4 | 0.236 | 22.273 | 0.984 | 55.678 |
| Quasi-model 5 | 0.245 | 20.063 | 0.984 | 31.392 |
| Quasi-model 6 | 0.234 | 22.083 | 0.984 | 30.370 |

| | | | | |
|---|---|---|---|---|
| Quasi-model 7 | 0.239 | 26.885 | 0.985 | 748.721 |
| Quasi-model 8 | 0.226 | 24.622 | 0.984 | 552.465 |
| Quasi-model 9 | 0.247 | 20.536 | 0.984 | 61.980 |
| Quasi-model 10 | 0.240 | 18.952 | 0.984 | 99.625 |
| Quasi-model 11 | 0.230 | 22.501 | 0.984 | 37.301 |
| Quasi-model 12 | 0.235 | 17.749 | 0.984 | 65.405 |
| Quasi-model 13 | 0.255 | 16.665 | 0.985 | 1869.622 |
| Quasi-model 14 | 0.241 | 18.219 | 0.984 | 293.096 |
| Quasi-model 15 | 0.219 | 22.199 | 0.983 | 44.871 |
| Quasi-model 16 | 0.243 | 22.133 | 0.984 | 33.596 |
| Quasi-model 17 | 0.235 | 20.760 | 0.984 | 104.395 |
| Quasi-model 18 | 0.227 | 23.572 | 0.984 | 107.300 |
| Quasi-model 19 | 0.252 | 21.990 | 0.985 | 88.908 |
| Quasi-model 20 | 0.254 | 23.190 | 0.985 | 52.625 |
| Quasi-model 21 | 0.252 | 23.582 | 0.985 | 771.321 |
| Quasi-model 22 | 0.230 | 21.958 | 0.984 | 150.082 |
| Quasi-model 23 | 0.235 | 19.995 | 0.984 | 241.024 |
| Quasi-model 24 | 0.238 | 21.255 | 0.984 | 26.125 |
| Quasi-model 25 | 0.228 | 23.013 | 0.984 | 247.559 |
| Quasi-model 26 | 0.236 | 23.174 | 0.984 | 115.670 |
| Quasi-model 27 | 0.222 | 22.320 | 0.984 | 33.348 |
| Quasi-model 28 | 0.214 | 24.808 | 0.984 | 155.909 |
| Quasi-model 29 | 0.238 | 22.496 | 0.984 | 78.532 |
| Quasi-model 30 | 0.228 | 24.052 | 0.984 | 469.683 |

*Model #2: Two compartments, no covariate*

| **Model** | **K** | **KCP** | **KPC** | **V** | **$R^2$** | **MSE** |
|---|---|---|---|---|---|---|
| Pop-PK, NPAG | 0.492 | 1.857 | 1.659 | 9.350 | 0.995 | 16.700 |
| Pop-PK, MAP Bayesian | 0.489 | 1.845 | 1.667 | 9.234 | 0.988 | 22.156 |
| Quasi-model 1 | 0.360 | 1.668 | 3.75 | 14.449 | 0.988 | 52.244 |
| Quasi-model 2 | 0.404 | 1.404 | 3.043 | 12.886 | 0.988 | 145.299 |
| Quasi-model 3 | 0.578 | 1.807 | 1.719 | 11.287 | 0.986 | 325.073 |
| Quasi-model 4 | 0.471 | 3.464 | 3.769 | 10.237 | 0.987 | 136.782 |
| Quasi-model 5 | 0.340 | 1.874 | 3.411 | 13.811 | 0.988 | 23.082 |
| Quasi-model 6 | 0.472 | 2.83 | 3.274 | 11.693 | 0.988 | 64.569 |
| Quasi-model 7 | 0.368 | 1.234 | 1.958 | 10.196 | 0.99 | 1322.221 |
| Quasi-model 8 | 0.285 | 0.572 | 2.087 | 19.260 | 0.99 | 102.488 |
| Quasi-model 9 | 0.349 | 1.303 | 2.064 | 13.156 | 0.99 | 248.914 |
| Quasi-model 10 | 0.388 | 2.901 | 4.678 | 11.857 | 0.986 | 186.467 |
| Quasi-model 11 | 0.365 | 1.449 | 2.635 | 14.546 | 0.989 | 18.442 |
| Quasi-model 12 | 0.342 | 1.044 | 4.030 | 16.168 | 0.987 | 23.909 |
| Quasi-model 13 | 0.356 | 2.234 | 3.220 | 13.057 | 0.989 | 56.026 |
| Quasi-model 14 | 0.372 | 2.349 | 3.613 | 10.655 | 0.987 | 45.648 |
| Quasi-model 15 | 0.432 | 2.317 | 2.903 | 11.036 | 0.988 | 30.522 |
| Quasi-model 16 | 0.360 | 1.114 | 2.005 | 13.099 | 0.9900 | 39.862 |
| Quasi-model 17 | 0.406 | 1.409 | 3.035 | 12.855 | 0.989 | 223.929 |

| | | | | | | |
|---|---|---|---|---|---|---|
| Quasi-model 18 | 0.673 | 1.801 | 0.932 | 6.957 | 0.979 | 195.212 |
| Quasi-model 19 | 0.471 | 3.456 | 3.767 | 10.384 | 0.987 | 142.640 |
| Quasi-model 20 | 0.339 | 1.868 | 3.919 | 13.789 | 0.988 | 36.921 |
| Quasi-model 21 | 0.467 | 2.828 | 3.266 | 11.660 | 0.988 | 106.967 |
| Quasi-model 22 | 0.431 | 3.599 | 3.974 | 11.458 | 0.988 | 38.406 |
| Quasi-model 23 | 0.285 | 0.572 | 2.108 | 19.260 | 0.990 | 119.557 |
| Quasi-model 24 | 0.372 | 1.341 | 2.242 | 10.954 | 0.990 | 64.256 |
| Quasi-model 25 | 0.390 | 2.898 | 4.566 | 11.819 | 0.987 | 221.401 |
| Quasi-model 26 | 0.366 | 2.202 | 3.288 | 12.330 | 0.989 | 121.609 |
| Quasi-model 27 | 0.394 | 2.042 | 1.861 | 11.682 | 0.988 | 391.949 |
| Quasi-model 28 | 0.354 | 0.935 | 1.915 | 13.469 | 0.990 | 49.764 |
| Quasi-model 29 | 0.330 | 1.191 | 3.215 | 15.741 | 0.988 | 35.015 |
| Quasi-model 30 | 0.382 | 1.766 | 2.256 | 13.942 | 0.990 | 18.822 |

*Model #3: One compartment, covariate: creatinine clearance*

| **Model** | **KS** | **KI** | **$V_c$** | **$R^2$** | **MSE** |
|---|---|---|---|---|---|
| Pop-PK, NPAG | 0.004 | 0.052 | 20.525 | 0.992 | 26.005 |
| Pop-PK, MAP Bayesian | 0.004 | 0.096 | 36.503 | 0.977 | 1930.343 |
| Quasi-model 1 | 0.003 | 0.100 | 78.121 | 0.977 | 16163.582 |
| Quasi-model 2 | 0.004 | 0.100 | 77.032 | 0.977 | 15867.909 |
| Quasi-model 3 | 0.005 | 0.099 | 76.452 | 0.977 | 15798.651 |
| Quasi-model 4 | 0.002 | 0.099 | 79.390 | 0.977 | 16320.148 |
| Quasi-model 5 | 0.002 | 0.097 | 79.442 | 0.977 | 16225.541 |
| Quasi-model 6 | 0.002 | 0.100 | 79.479 | 0.977 | 16435.030 |
| Quasi-model 7 | 0.002 | 0.097 | 79.155 | 0.977 | 16232.114 |
| Quasi-model 8 | 0.005 | 0.097 | 79.514 | 0.977 | 16254.626 |
| Quasi-model 9 | 0.004 | 0.100 | 79.357 | 0.977 | 16439.949 |
| Quasi-model 10 | 0.003 | 0.100 | 77.089 | 0.977 | 15813.633 |
| Quasi-model 11 | 0.003 | 0.100 | 72.783 | 0.977 | 14741.318 |
| Quasi-model 12 | 0.003 | 0.097 | 79.571 | 0.977 | 16291.543 |
| Quasi-model 13 | 0.003 | 0.098 | 77.461 | 0.977 | 15908.973 |
| Quasi-model 14 | 0.001 | 0.099 | 73.635 | 0.977 | 15188.758 |
| Quasi-model 15 | 0.004 | 0.099 | 74.543 | 0.977 | 15075.430 |
| Quasi-model 16 | 0.003 | 0.096 | 78.022 | 0.977 | 15691.112 |
| Quasi-model 17 | 0.004 | 0.095 | 79.382 | 0.976 | 16130.073 |
| Quasi-model 18 | 0.005 | 0.099 | 79.734 | 0.977 | 16462.576 |
| Quasi-model 19 | 0.002 | 0.097 | 79.733 | 0.977 | 16383.775 |
| Quasi-model 20 | 0.005 | 0.099 | 74.661 | 0.977 | 15282.424 |
| Quasi-model 21 | 0.003 | 0.097 | 76.989 | 0.977 | 15695.800 |
| Quasi-model 22 | 0.005 | 0.097 | 78.009 | 0.977 | 16008.559 |
| Quasi-model 23 | 0.002 | 0.100 | 79.421 | 0.977 | 16501.559 |
| Quasi-model 24 | 0.003 | 0.094 | 79.653 | 0.977 | 15797.891 |
| Quasi-model 25 | 0.004 | 0.094 | 78.279 | 0.976 | 15874.993 |
| Quasi-model 26 | 0.004 | 0.099 | 74.898 | 0.977 | 15275.141 |
| Quasi-model 27 | 0.005 | 0.095 | 79.405 | 0.976 | 16141.837 |
| Quasi-model 28 | 0.002 | 0.092 | 79.424 | 0.976 | 15865.659 |

| | | | | | |
|---|---|---|---|---|---|
| Quasi-model 29 | 0.005 | 0.091 | 79.676 | 0.976 | 15525.113 |
| Quasi-model 30 | 0.001 | 0.099 | 77.762 | 0.977 | 16027.284 |

*Model #4: Two compartments, covariate: creatinine clearance*

| Model | KS | KCP | KPC | KI | $V_c$ | $R^2$ | MSE |
|---|---|---|---|---|---|---|---|
| Pop-PK, NPAG | 0.014 | 3.419 | 1.099 | 0.18 | 10.300 | 0.988 | 7.315 |
| Pop-PK, MAP Bayesian | 0.014 | 3.391 | 1.118 | 0.181 | 10.252 | 0.976 | 7.363 |
| Quasi-model 1 | 0.013 | 3.157 | 3.189 | 0.201 | 16.414 | 0.989 | 529.846 |
| Quasi-model 2 | 0.013 | 3.182 | 3.262 | 0.193 | 17.159 | 0.989 | 607.965 |
| Quasi-model 3 | 0.012 | 0.458 | 4.627 | 0.264 | 17.574 | 0.987 | 35.319 |
| Quasi-model 4 | 0.012 | 3.077 | 3.089 | 0.198 | 16.868 | 0.989 | 524.862 |
| Quasi-model 5 | 0.012 | 2.919 | 3.024 | 0.182 | 16.356 | 0.989 | 508.390 |
| Quasi-model 6 | 0.013 | 3.112 | 3.120 | 0.191 | 16.108 | 0.989 | 519.691 |
| Quasi-model 7 | 0.013 | 2.888 | 3.021 | 0.182 | 15.417 | 0.989 | 519.748 |
| Quasi-model 8 | 0.014 | 2.980 | 3.250 | 0.191 | 16.680 | 0.989 | 552.896 |
| Quasi-model 9 | 0.014 | 3.142 | 3.086 | 0.188 | 16.514 | 0.989 | 629.830 |
| Quasi-model 10 | 0.012 | 3.185 | 3.049 | 0.198 | 16.276 | 0.989 | 538.061 |
| Quasi-model 11 | 0.001 | 0.826 | 2.628 | 0.280 | 16.033 | 0.989 | 32.942 |
| Quasi-model 12 | 0.009 | 0.706 | 2.694 | 0.298 | 18.400 | 0.988 | 293.612 |
| Quasi-model 13 | 0.022 | 0.947 | 4.729 | 0.289 | 16.667 | 0.986 | 82.486 |
| Quasi-model 14 | 0.006 | 0.545 | 1.738 | 0.304 | 18.070 | 0.989 | 167.200 |
| Quasi-model 15 | 0.007 | 1.029 | 3.482 | 0.294 | 15.798 | 0.989 | 178.237 |
| Quasi-model 16 | 0.015 | 0.887 | 4.643 | 0.277 | 17.035 | 0.986 | 96.474 |
| Quasi-model 17 | 0.006 | 0.572 | 3.511 | 0.254 | 20.238 | 0.986 | 26.113 |
| Quasi-model 18 | 0.011 | 0.360 | 1.052 | 0.284 | 16.531 | 0.989 | 23.742 |
| Quasi-model 19 | 0.021 | 0.693 | 2.532 | 0.291 | 18.019 | 0.988 | 50.920 |
| Quasi-model 20 | 0.009 | 1.041 | 3.646 | 0.312 | 16.488 | 0.988 | 33.384 |
| Quasi-model 21 | 0.007 | 1.463 | 4.085 | 0.310 | 13.917 | 0.986 | 88.063 |
| Quasi-model 22 | 0.015 | 1.260 | 5.075 | 0.291 | 17.021 | 0.986 | 31.053 |
| Quasi-model 23 | 0.014 | 1.018 | 3.413 | 0.304 | 15.571 | 0.987 | 44.687 |
| Quasi-model 24 | 0.013 | 0.222 | 4.129 | 0.253 | 20.159 | 0.987 | 186.474 |
| Quasi-model 25 | 0.007 | 1.265 | 4.339 | 0.293 | 16.523 | 0.986 | 29.710 |
| Quasi-model 26 | 0.008 | 0.550 | 2.730 | 0.284 | 16.723 | 0.989 | 57.429 |
| Quasi-model 27 | 0.013 | 1.202 | 3.542 | 0.314 | 16.159 | 0.987 | 26.736 |
| Quasi-model 28 | 0.010 | 0.832 | 4.231 | 0.290 | 13.840 | 0.986 | 462.577 |
| Quasi-model 29 | 0.016 | 1.940 | 5.403 | 0.313 | 15.823 | 0.985 | 35.517 |
| Quasi-model 30 | 0.012 | 0.476 | 1.749 | 0.319 | 15.729 | 0.989 | 37.328 |

**Supplementary table S11: Performance of the quasi-models for Subject 10**

*Model #1: One compartment, no covariate*

| Model | K | V | $R^2$ | MSE |
|---|---|---|---|---|
| Pop-PK, NPAG | 0.266 | 68.95 | 0.985 | 4.579 |
| Pop-PK, MAP | 0.367 | 61.445 | 0.831 | 28.284 |
| Quasi-model 1 | 0.336 | 60.789 | 0.834 | 26.685 |

| | | | | |
|---|---|---|---|---|
| Quasi-model 2 | 0.367 | 57.237 | 0.832 | 28.923 |
| Quasi-model 3 | 0.318000 | 64.675 | 0.834 | 27.269 |
| Quasi-model 4 | 0.294 | 68.053 | 0.834 | 27.010 |
| Quasi-model 5 | 0.298 | 68.454 | 0.833 | 27.770 |
| Quasi-model 6 | 0.354 | 59.167 | 0.833 | 27.689 |
| Quasi-model 7 | 0.293 | 68.609 | 0.833 | 27.783 |
| Quasi-model 8 | 0.298 | 66.901 | 0.834 | 28.364 |
| Quasi-model 9 | 0.318 | 63.089 | 0.833 | 26.429 |
| Quasi-model 10 | 0.349 | 59.702 | 0.833 | 28.762 |
| Quasi-model 11 | 0.332 | 61.26 | 0.834 | 26.617 |
| Quasi-model 12 | 0.309 | 65.944 | 0.834 | 26.142 |
| Quasi-model 13 | 0.356 | 60.359 | 0.833 | 26.864 |
| Quasi-model 14 | 0.332 | 61.948 | 0.834 | 27.499 |
| Quasi-model 15 | 0.367 | 57.874 | 0.832 | 26.897 |
| Quasi-model 16 | 0.322 | 62.970 | 0.834 | 25.978 |
| Quasi-model 17 | 0.319 | 63.644 | 0.834 | 26.057 |
| Quasi-model 18 | 0.294 | 68.011 | 0.834 | 27.089 |
| Quasi-model 19 | 0.326 | 64.117 | 0.834 | 27.527 |
| Quasi-model 20 | 0.318 | 67.011 | 0.832 | 28.593 |
| Quasi-model 21 | 0.321 | 62.971 | 0.834 | 26.025 |
| Quasi-model 22 | 0.334 | 63.292 | 0.834 | 26.704 |
| Quasi-model 23 | 0.293 | 69.726 | 0.833 | 27.56 |
| Quasi-model 24 | 0.308 | 65.294 | 0.834 | 26.543 |
| Quasi-model 25 | 0.309 | 65.437 | 0.834 | 26.879 |
| Quasi-model 26 | 0.321 | 62.620 | 0.834 | 26.018 |
| Quasi-model 27 | 0.334 | 63.852 | 0.834 | 30.776 |
| Quasi-model 28 | 0.336 | 62.327 | 0.834 | 26.696 |
| Quasi-model 29 | 0.319 | 62.919 | 0.834 | 26.313 |
| Quasi-model 30 | 0.303 | 65.486 | 0.834 | 26.604 |

*Model #2: Two compartments, no covariate*

| **Model** | **K** | **KCP** | **KPC** | **V** | **$R^2$** | **MSE** |
|---|---|---|---|---|---|---|
| Pop-PK, NPAG | 0.684 | 3.292 | 2.203 | 24.65 | 0.987 | 4.103 |
| Pop-PK, MAP Bayesian | 1.360 | 1.282 | 0.719 | 23.853 | 0.136 | 864.826 |
| Quasi-model 1 | 0.818 | 4.498 | 4.533 | 33.334 | 0.568 | 525.761 |
| Quasi-model 2 | 1.230 | 2.434 | 3.412 | 29.031 | 0.447 | 879.033 |
| Quasi-model 3 | 1.346 | 2.182 | 3.505 | 27.282 | 0.490 | 802.525 |
| Quasi-model 4 | 1.337 | 2.857 | 2.949 | 30.042 | 0.408 | 929.952 |
| Quasi-model 5 | 1.349 | 3.050 | 3.093 | 29.675 | 0.483 | 842.914 |
| Quasi-model 6 | 0.844 | 4.543 | 4.544 | 34.768 | 0.554 | 455.938 |
| Quasi-model 7 | 1.411 | 2.183 | 2.829 | 29.552 | 0.419 | 936.534 |
| Quasi-model 8 | 1.378 | 1.805 | 3.387 | 29.303 | 0.439 | 881.256 |
| Quasi-model 9 | 1.437 | 2.681 | 2.761 | 27.428 | 0.372 | 1029.35 |
| Quasi-model 10 | 1.389 | 2.405 | 2.777 | 25.669 | 0.369 | 994.131 |
| Quasi-model 11 | 1.360 | 1.822 | 2.986 | 28.435 | 0.474 | 784.102 |
| Quasi-model 12 | 1.234 | 2.053 | 3.429 | 30.236 | 0.446 | 901.496 |

| | | | | | | |
|---|---|---|---|---|---|---|
| Quasi-model 13 | 1.320 | 2.970 | 3.234 | 28.982 | 0.482 | 835.738 |
| Quasi-model 14 | 1.475 | 2.353 | 3.119 | 25.159 | 0.370 | 966.538 |
| Quasi-model 15 | 1.481 | 2.374 | 3.465 | 25.594 | 0.386 | 920.791 |
| Quasi-model 16 | 0.818 | 4.482 | 4.533 | 33.335 | 0.563 | 528.200 |
| Quasi-model 17 | 1.291 | 2.475 | 2.914 | 29.038 | 0.400 | 954.121 |
| Quasi-model 18 | 1.341 | 2.138 | 3.236 | 27.223 | 0.481 | 795.990 |
| Quasi-model 19 | 1.333 | 2.284 | 2.909 | 30.031 | 0.429 | 906.854 |
| Quasi-model 20 | 1.297 | 2.531 | 3.022 | 30.828 | 0.467 | 847.032 |
| Quasi-model 21 | 1.308 | 2.640 | 2.710 | 29.886 | 0.407 | 928.141 |
| Quasi-model 22 | 1.393 | 2.175 | 3.018 | 29.539 | 0.424 | 907.659 |
| Quasi-model 23 | 1.386 | 1.799 | 3.361 | 29.052 | 0.434 | 910.633 |
| Quasi-model 24 | 1.376 | 2.477 | 2.470 | 27.246 | 0.292 | 1107.884 |
| Quasi-model 25 | 1.396 | 3.743 | 2.911 | 28.128 | 0.409 | 944.231 |
| Quasi-model 26 | 1.333 | 2.363 | 3.524 | 30.360 | 0.494 | 873.237 |
| Quasi-model 27 | 1.348 | 3.171 | 3.437 | 29.579 | 0.480 | 882.499 |
| Quasi-model 28 | 1.358 | 2.990 | 2.951 | 27.813 | 0.469 | 855.140 |
| Quasi-model 29 | 1.242 | 1.464 | 3.193 | 27.988 | 0.427 | 882.819 |
| Quasi-model 30 | 1.425 | 2.351 | 3.085 | 28.259 | 0.492 | 827.174 |

*Model #3: One compartment, covariate: creatinine clearance*

| **Model** | **KS** | **KI** | **$V_c$** | **$R^2$** | **MSE** |
|---|---|---|---|---|---|
| Pop-PK, NPAG | 0.004 | 0.022 | 72.525 | 0.985 | 5.318 |
| Pop-PK, MAP Bayesian | 0.004 | 0.022 | 72.608 | 0.827 | 33.910 |
| Quasi-model 1 | 0.005 | 0.028 | 65.105 | 0.834 | 26.792 |
| Quasi-model 2 | 0.005 | 0.049 | 61.456 | 0.833 | 26.455 |
| Quasi-model 3 | 0.004 | 0.048 | 67.713 | 0.834 | 26.704 |
| Quasi-model 4 | 0.005 | 0.020 | 62.854 | 0.834 | 26.190 |
| Quasi-model 5 | 0.004 | 0.076 | 63.912 | 0.834 | 26.835 |
| Quasi-model 6 | 0.004 | 0.065 | 64.453 | 0.834 | 25.999 |
| Quasi-model 7 | 0.004 | 0.054 | 64.778 | 0.834 | 26.900 |
| Quasi-model 8 | 0.004 | 0.046 | 64.613 | 0.834 | 26.490 |
| Quasi-model 9 | 0.004 | 0.580 | 63.437 | 0.833 | 26.868 |
| Quasi-model 10 | 0.005 | 0.016 | 65.495 | 0.834 | 26.781 |
| Quasi-model 11 | 0.004 | 0.055 | 65.421 | 0.834 | 27.089 |
| Quasi-model 12 | 0.004 | 0.040 | 62.381 | 0.834 | 26.316 |
| Quasi-model 13 | 0.004 | 0.046 | 65.518 | 0.833 | 26.534 |
| Quasi-model 14 | 0.004 | 0.052 | 65.008 | 0.834 | 26.425 |
| Quasi-model 15 | 0.004 | 0.073 | 62.997 | 0.834 | 27.309 |
| Quasi-model 16 | 0.004 | 0.043 | 65.569 | 0.834 | 26.832 |
| Quasi-model 17 | 0.004 | 0.080 | 65.072 | 0.834 | 26.789 |
| Quasi-model 18 | 0.004 | 0.043 | 63.366 | 0.834 | 26.433 |
| Quasi-model 19 | 0.004 | 0.089 | 63.666 | 0.834 | 27.128 |
| Quasi-model 20 | 0.004 | 0.078 | 61.999 | 0.833 | 26.826 |
| Quasi-model 21 | 0.004 | 0.077 | 66.500 | 0.834 | 26.893 |
| Quasi-model 22 | 0.004 | 0.039 | 64.727 | 0.833 | 26.528 |
| Quasi-model 23 | 0.004 | 0.048 | 66.078 | 0.834 | 27.004 |

| | | | | | |
|---|---|---|---|---|---|
| Quasi-model 24 | 0.004 | 0.042 | 64.934 | 0.833 | 27.409 |
| Quasi-model 25 | 0.004 | 0.066 | 65.472 | 0.834 | 26.195 |
| Quasi-model 26 | 0.004 | 0.057 | 62.382 | 0.834 | 26.178 |
| Quasi-model 27 | 0.005 | 0.019 | 61.785 | 0.834 | 27.284 |
| Quasi-model 28 | 0.004 | 0.039 | 65.083 | 0.833 | 27.236 |
| Quasi-model 29 | 0.004 | 0.069 | 63.231 | 0.834 | 27.150 |
| Quasi-model 30 | 0.004 | 0.042 | 62.769 | 0.834 | 27.056 |

*Model #4: Two compartments, covariate: creatinine clearance*

| Model | KS | KCP | KPC | KI | $V_c$ | $R^2$ | MSE |
|---|---|---|---|---|---|---|---|
| Pop-PK, NPAG | 0.009 | 3.709 | 2.027 | 0.212 | 21.300 | 0.987 | 4.110 |
| Pop-PK, MAP Bayesian | 0.009 | 3.736 | 2.046 | 0.214 | 21.337 | 0.513 | 139.395 |
| Quasi-model 1 | 0.021 | 2.535 | 4.806 | 0.197 | 21.674 | 0.415 | 898.005 |
| Quasi-model 2 | 0.020 | 1.927 | 4.889 | 0.251 | 22.297 | 0.457 | 805.901 |
| Quasi-model 3 | 0.020 | 2.195 | 3.627 | 0.215 | 23.853 | 0.469 | 818.374 |
| Quasi-model 4 | 0.023 | 3.582 | 4.945 | 0.068 | 24.107 | 0.469 | 801.018 |
| Quasi-model 5 | 0.023 | 1.819 | 4.654 | 0.242 | 22.653 | 0.425 | 892.293 |
| Quasi-model 6 | 0.023 | 1.651 | 4.322 | 0.205 | 21.620 | 0.393 | 940.515 |
| Quasi-model 7 | 0.021 | 1.975 | 4.369 | 0.231 | 23.975 | 0.465 | 798.546 |
| Quasi-model 8 | 0.022 | 2.226 | 4.272 | 0.255 | 22.442 | 0.453 | 837.403 |
| Quasi-model 9 | 0.023 | 2.392 | 4.422 | 0.178 | 20.746 | 0.410 | 882.119 |
| Quasi-model 10 | 0.023 | 2.297 | 4.527 | 0.219 | 21.905 | 0.433 | 850.569 |
| Quasi-model 11 | 0.021 | 1.216 | 4.372 | 0.135 | 22.506 | 0.467 | 743.067 |
| Quasi-model 12 | 0.022 | 1.666 | 4.299 | 0.247 | 22.430 | 0.433 | 862.366 |
| Quasi-model 13 | 0.023 | 0.850 | 3.794 | 0.221 | 21.070 | 0.359 | 971.746 |
| Quasi-model 14 | 0.023 | 3.233 | 4.811 | 0.283 | 21.865 | 0.390 | 980.739 |
| Quasi-model 15 | 0.021 | 1.450 | 4.592 | 0.191 | 21.724 | 0.428 | 827.373 |
| Quasi-model 16 | 0.020 | 0.804 | 4.135 | 0.216 | 21.986 | 0.421 | 844.144 |
| Quasi-model 17 | 0.022 | 1.517 | 4.171 | 0.235 | 20.869 | 0.396 | 892.871 |
| Quasi-model 18 | 0.023 | 2.438 | 5.500 | 0.230 | 21.753 | 0.471 | 828.392 |
| Quasi-model 19 | 0.022 | 3.208 | 4.101 | 0.184 | 23.119 | 0.443 | 889.972 |
| Quasi-model 20 | 0.023 | 2.628 | 4.830 | 0.201 | 21.977 | 0.424 | 883.104 |
| Quasi-model 21 | 0.021 | 3.430 | 4.793 | 0.208 | 23.227 | 0.457 | 843.163 |
| Quasi-model 22 | 0.021 | 2.918 | 3.960 | 0.180 | 23.475 | 0.416 | 910.886 |
| Quasi-model 23 | 0.021 | 1.406 | 4.012 | 0.161 | 22.332 | 0.440 | 824.754 |
| Quasi-model 24 | 0.023 | 2.513 | 4.270 | 0.230 | 21.915 | 0.371 | 999.343 |
| Quasi-model 25 | 0.022 | 3.272 | 3.886 | 0.284 | 22.014 | 0.437 | 820.329 |
| Quasi-model 26 | 0.023 | 1.811 | 4.151 | 0.236 | 23.377 | 0.428 | 894.251 |
| Quasi-model 27 | 0.023 | 1.284 | 3.976 | 0.201 | 21.936 | 0.409 | 882.167 |
| Quasi-model 28 | 0.022 | 2.341 | 4.587 | 0.239 | 22.039 | 0.450 | 855.283 |
| Quasi-model 29 | 0.021 | 0.823 | 4.001 | 0.112 | 23.394 | 0.411 | 850.15 |
| Quasi-model 30 | 0.021 | 0.871 | 4.629 | 0.114 | 23.019 | 0.454 | 810.809 |

**Supplementary table S12: Performance of the quasi-models for Subject 11**

*Model #1: One compartment, no covariate*

| Model | K | V | $R^2$ | MSE |
|---|---|---|---|---|
| Pop-PK, NPAG | 0.266 | 22.150 | 0.976 | 101.473 |
| Pop-PK, MAP | 0.262 | 21.921 | 0.953 | 99.635 |
| Quasi-model 1 | 0.292 | 19.809 | 0.953 | 127.756 |
| Quasi-model 2 | 0.341 | 16.506 | 0.952 | 328.750 |
| Quasi-model 3 | 0.290 | 19.783 | 0.953 | 127.756 |
| Quasi-model 4 | 0.258 | 23.160 | 0.953 | 169.809 |
| Quasi-model 5 | 0.219 | 24.984 | 0.953 | 156.335 |
| Quasi-model 6 | 0.253 | 22.311 | 0.953 | 103.246 |
| Quasi-model 7 | 0.301 | 18.856 | 0.953 | 164.157 |
| Quasi-model 8 | 0.269 | 20.863 | 0.953 | 113.778 |
| Quasi-model 9 | 0.265 | 21.264 | 0.953 | 142.134 |
| Quasi-model 10 | 0.253 | 24.250 | 0.953 | 276.879 |
| Quasi-model 11 | 0.269 | 20.755 | 0.953 | 133.241 |
| Quasi-model 12 | 0.288 | 20.699 | 0.953 | 142.558 |
| Quasi-model 13 | 0.285 | 20.796 | 0.953 | 105.865 |
| Quasi-model 14 | 0.288 | 19.990 | 0.953 | 126.849 |
| Quasi-model 15 | 0.302 | 19.007 | 0.953 | 213.777 |
| Quasi-model 16 | 0.299 | 19.106 | 0.953 | 206.347 |
| Quasi-model 17 | 0.299 | 20.148 | 0.953 | 159.781 |
| Quasi-model 18 | 0.227 | 23.748 | 0.953 | 153.461 |
| Quasi-model 19 | 0.255 | 22.214 | 0.953 | 106.674 |
| Quasi-model 20 | 0.267 | 22.033 | 0.953 | 104.162 |
| Quasi-model 21 | 0.274 | 20.899 | 0.953 | 99.281 |
| Quasi-model 22 | 0.266 | 21.161 | 0.953 | 146.851 |
| Quasi-model 23 | 0.255 | 22.719 | 0.953 | 110.761 |
| Quasi-model 24 | 0.225 | 24.442 | 0.953 | 121.492 |
| Quasi-model 25 | 0.225 | 25.719 | 0.953 | 213.263 |
| Quasi-model 26 | 0.236 | 23.217 | 0.953 | 159.168 |
| Quasi-model 27 | 0.268 | 20.566 | 0.953 | 248.588 |
| Quasi-model 28 | 0.208 | 26.117 | 0.953 | 195.461 |
| Quasi-model 29 | 0.237 | 23.276 | 0.953 | 148.983 |
| Quasi-model 30 | 0.242 | 24.146 | 0.953 | 131.155 |

*Model #2: Two compartments, no covariate*

| Model | K | KCP | KPC | V | $R^2$ | MSE |
|---|---|---|---|---|---|---|
| Pop-PK, NPAG | 0.732 | 4.579 | 2.847 | 7.550 | 0.978 | 92.405 |
| Pop-PK, MAP Bayesian | 0.723 | 4.551 | 2.826 | 7.488 | 0.958 | 89.197 |
| Quasi-model 1 | 0.527 | 2.713 | 3.859 | 10.246 | 0.957 | 96.929 |
| Quasi-model 2 | 0.334 | 0.444 | 1.908 | 17.242 | 0.954 | 103.233 |
| Quasi-model 3 | 0.487 | 2.805 | 4.543 | 10.589 | 0.955 | 235.215 |
| Quasi-model 4 | 0.456 | 3.015 | 4.670 | 13.026 | 0.955 | 155.442 |
| Quasi-model 5 | 0.464 | 3.305 | 3.891 | 13.189 | 0.956 | 610.397 |
| Quasi-model 6 | 0.474 | 2.838 | 3.276 | 11.667 | 0.957 | 168.720 |
| Quasi-model 7 | 0.831 | 4.853 | 2.307 | 6.054 | 0.959 | 124.397 |
| Quasi-model 8 | 0.797 | 4.948 | 2.506 | 7.981 | 0.958 | 889.947 |

| | | | | | | |
|---|---|---|---|---|---|---|
| Quasi-model 9 | 0.448 | 2.171 | 3.607 | 12.719 | 0.956 | 123.976 |
| Quasi-model 10 | 0.505 | 3.081 | 3.829 | 11.330 | 0.956 | 163.962 |
| Quasi-model 11 | 0.361 | 0.996 | 2.244 | 15.008 | 0.955 | 100.766 |
| Quasi-model 12 | 0.347 | 1.060 | 2544.000 | 17.864 | 0.956 | 528.255 |
| Quasi-model 13 | 0.801 | 3.838 | 2.132 | 6.489 | 0.959 | 104.945 |
| Quasi-model 14 | 0.549 | 4.378 | 3.609 | 10.597 | 0.957 | 258.327 |
| Quasi-model 15 | 0.409 | 1.841 | 4.650 | 14.060 | 0.955 | 127.912 |
| Quasi-model 16 | 0.635 | 2.734 | 2.331 | 8.383 | 0.957 | 150.945 |
| Quasi-model 17 | 0.334 | 0.440 | 1.908 | 17.242 | 0.954 | 103.227 |
| Quasi-model 18 | 0.486 | 2.802 | 4.547 | 10.658 | 0.955 | 146.014 |
| Quasi-model 19 | 0.456 | 3.008 | 4.674 | 13.017 | 0.955 | 154.793 |
| Quasi-model 20 | 0.464 | 3.298 | 3.907 | 13.189 | 0.956 | 611.578 |
| Quasi-model 21 | 0.471 | 2.838 | 3.277 | 11.664 | 0.956 | 150.691 |
| Quasi-model 22 | 0.529 | 4.358 | 4.350 | 10.377 | 0.956 | 179.500 |
| Quasi-model 23 | 0.795 | 4.953 | 2.511 | 8.015 | 0.958 | 815.489 |
| Quasi-model 24 | 0.448 | 2.171 | 3.602 | 12.716 | 0.956 | 115.852 |
| Quasi-model 25 | 0.505 | 373.000 | 3.842 | 11.330 | 0.956 | 163.945 |
| Quasi-model 26 | 0.607 | 3.662 | 2.713 | 10.036 | 0.958 | 683.446 |
| Quasi-model 27 | 0.487 | 2.779 | 3.719 | 12.314 | 0.956 | 281.181 |
| Quasi-model 28 | 0.686 | 2.697 | 1.964 | 7.249 | 0.957 | 179.951 |
| Quasi-model 29 | 0.619 | 3.365 | 2.891 | 9.518 | 0.957 | 237.014 |
| Quasi-model 30 | 0.971 | 4.912 | 2.082 | 6.209 | 0.959 | 917.214 |

*Model #3: One compartment, covariate: creatinine clearance*

| **Model** | **KS** | **KI** | **$V_c$** | **$R^2$** | **MSE** |
|---|---|---|---|---|---|
| Pop-PK, NPAG | 0.004 | 0.026 | 21.825 | 0.976 | 101.110 |
| Pop-PK, MAP Bayesian | 0.003 | 0.027 | 26.123 | 0.953 | 146.414 |
| Quasi-model 1 | 0.004 | 0.013 | 24.500 | 0.953 | 170.588 |
| Quasi-model 2 | 0.004 | 0.066 | 22.198 | 0.953 | 124.029 |
| Quasi-model 3 | 0.004 | 0.028 | 20.592 | 0.953 | 122.371 |
| Quasi-model 4 | 0.003 | 0.095 | 21.022 | 0.953 | 102.489 |
| Quasi-model 5 | 0.004 | 0.063 | 20.301 | 0.953 | 102.558 |
| Quasi-model 6 | 0.004 | 0.031 | 24.312 | 0.953 | 214.268 |
| Quasi-model 7 | 0.005 | 0.019 | 20.998 | 0.953 | 115.970 |
| Quasi-model 8 | 0.005 | 0.008 | 22.186 | 0.953 | 107.427 |
| Quasi-model 9 | 0.004 | 0.044 | 22.531 | 0.953 | 103.386 |
| Quasi-model 10 | 0.003 | 0.100 | 19.528 | 0.953 | 128.274 |
| Quasi-model 11 | 0.003 | 0.066 | 25.328 | 0.953 | 183.048 |
| Quasi-model 12 | 0.003 | 0.084 | 23.259 | 0.953 | 118.236 |
| Quasi-model 13 | 0.004 | 0.058 | 20.936 | 0.953 | 121.303 |
| Quasi-model 14 | 0.004 | 0.039 | 21.455 | 0.953 | 171.447 |
| Quasi-model 15 | 0.004 | 0.024 | 22.180 | 0.953 | 100.296 |
| Quasi-model 16 | 0.003 | 0.075 | 21.687 | 0.953 | 106.323 |
| Quasi-model 17 | 0.003 | 0.072 | 23.252 | 0.953 | 108.526 |
| Quasi-model 18 | 0.004 | 0.049 | 20.302 | 0.953 | 117.863 |
| Quasi-model 19 | 0.003 | 0.071 | 21.048 | 0.953 | 99.798 |

| | | | | | |
|---|---|---|---|---|---|
| Quasi-model 20 | 0.005 | 0.014 | 22.622 | 0.953 | 151.291 |
| Quasi-model 21 | 0.004 | 0.071 | 19.804 | 0.953 | 124.283 |
| Quasi-model 22 | 0.004 | 0.035 | 23.531 | 0.953 | 116.217 |
| Quasi-model 23 | 0.004 | 0.076 | 19.260 | 0.953 | 142.133 |
| Quasi-model 24 | 0.004 | 0.015 | 22.844 | 0.953 | 116.793 |
| Quasi-model 25 | 0.004 | 0.024 | 21.988 | 0.953 | 100.736 |
| Quasi-model 26 | 0.004 | 0.010 | 21.970 | 0.953 | 108.947 |
| Quasi-model 27 | 0.003 | 0.077 | 21.900 | 0.953 | 119.534 |
| Quasi-model 28 | 0.004 | 0.021 | 22.876 | 0.953 | 108.982 |
| Quasi-model 29 | 0.004 | 0.046 | 21.188 | 0.953 | 99.517 |
| Quasi-model 30 | 0.003 | 0.077 | 20.530 | 0.953 | 161.467 |

*Model #4: Two compartments, covariate: creatinine clearance*

| **Model** | **KS** | **KCP** | **KPC** | **KI** | **$V_c$** | **$R^2$** | **MSE** |
|---|---|---|---|---|---|---|---|
| Pop-PK, NPAG | 0.004 | 2.085 | 2.375 | 0.264 | 10.500 | 0.978 | 92.342 |
| Pop-PK, MAP Bayesian | 0.004 | 2.089 | 2.361 | 0.264 | 10.510 | 0.957 | 161.934 |
| Quasi-model 1 | 0.004 | 4.928 | 5.426 | 0.301 | 11.511 | 0.955 | 237.251 |
| Quasi-model 2 | 0.003 | 2.793 | 4.378 | 0.251 | 12.629 | 0.955 | 197.933 |
| Quasi-model 3 | 0.005 | 2.058 | 4.461 | 0.120 | 14.446 | 0.956 | 100.612 |
| Quasi-model 4 | 0.002 | 4.060 | 4.818 | 0.344 | 12.883 | 0.955 | 520.767 |
| Quasi-model 5 | 0.004 | 3.744 | 5.307 | 0.137 | 13.476 | 0.956 | 117.756 |
| Quasi-model 6 | 0.008 | 3.149 | 2.381 | 0.173 | 8.334 | 0.958 | 108.690 |
| Quasi-model 7 | 0.004 | 0.525 | 4.412 | 0.046 | 18.994 | 0.954 | 104.869 |
| Quasi-model 8 | 0.003 | 2.268 | 3.442 | 0.227 | 14.085 | 0.955 | 103.936 |
| Quasi-model 9 | 0.008 | 5.137 | 4.453 | 0.105 | 10.106 | 0.955 | 223.256 |
| Quasi-model 10 | 0.004 | 1.322 | 4.558 | 0.113 | 15.932 | 0.955 | 123.264 |
| Quasi-model 11 | 0.006 | 2.055 | 4.036 | 0.051 | 14.150 | 0.956 | 127.437 |
| Quasi-model 12 | 0.007 | 4.374 | 5.189 | 0.060 | 10.126 | 0.956 | 117.745 |
| Quasi-model 13 | 0.005 | 0.795 | 5.335 | 0.007 | 18.366 | 0.954 | 97.326 |
| Quasi-model 14 | 0.006 | 3.957 | 5.348 | 0.155 | 12.243 | 0.956 | 133.900 |
| Quasi-model 15 | 0.003 | 1.661 | 5.781 | 0.136 | 18.660 | 0.55 | 133.122 |
| Quasi-model 16 | 0.008 | 3.071 | 3.888 | 0.036 | 11.825 | 0.956 | 130.281 |
| Quasi-model 17 | 0.007 | 4.818 | 4.453 | 0.048 | 11.958 | 0.955 | 171.460 |
| Quasi-model 18 | 0.006 | 3.509 | 2.954 | 0.196 | 10.400 | 0.957 | 94.461 |
| Quasi-model 19 | 0.002 | 1.577 | 5.591 | 0.227 | 15.890 | 0.954 | 108.326 |
| Quasi-model 20 | 0.004 | 2.743 | 4.418 | 0.324 | 11.393 | 0.955 | 114.088 |
| Quasi-model 21 | 0.004 | 5.765 | 5.184 | 0.325 | 9.721 | 0.956 | 96.681 |
| Quasi-model 22 | 0.006 | 2.427 | 5.278 | 0.074 | 14.883 | 0.956 | 111.772 |
| Quasi-model 23 | 0.001 | 1.723 | 3.699 | 0.321 | 13.575 | 0.956 | 102.592 |
| Quasi-model 24 | 0.006 | 3.605 | 3.199 | 0.132 | 10.018 | 0.957 | 422.481 |
| Quasi-model 25 | 0.005 | 3.343 | 4.942 | 0.138 | 12.800 | 0.955 | 199.790 |
| Quasi-model 26 | 0.004 | 2.052 | 3.712 | 0.170 | 14.204 | 0.957 | 147.176 |
| Quasi-model 27 | 0.007 | 4.007 | 4.197 | 0.185 | 9.658 | 0.956 | 97.329 |
| Quasi-model 28 | 0.001 | 2.167 | 4.675 | 0.244 | 13.112 | 0.954 | 312.944 |
| Quasi-model 29 | 0.003 | 1.320 | 3.389 | 0.181 | 15.005 | 0.955 | 98.360 |
| Quasi-model 30 | 0.003 | 2.501 | 4.309 | 0.286 | 14.642 | 0.956 | 195.419 |

**Supplementary table S13: Performance of the quasi-models for Subject 12**

*Model #1: One compartment, no covariate*

| Model | K | V | $R^2$ | MSE |
|---|---|---|---|---|
| Pop-PK, NPAG | 0.649 | 34.750 | 0.991 | 10.729 |
| Pop-PK, MAP | 0.648 | 34.565 | 0.982 | 10.744 |
| Quasi-model 1 | 0.598 | 37.946 | 0.980 | 13.619 |
| Quasi-model 2 | 0.622 | 34.816 | 0.981 | 11.621 |
| Quasi-model 3 | 0.632 | 36.479 | 0.981 | 13.548 |
| Quasi-model 4 | 0.657 | 34.588 | 0.983 | 10.873 |
| Quasi-model 5 | 0.602 | 39.011 | 0.981 | 17.660 |
| Quasi-model 6 | 0.654 | 35.563 | 0.982 | 14.504 |
| Quasi-model 7 | 0.599 | 38.806 | 0.980 | 17.077 |
| Quasi-model 8 | 0.652 | 33.686 | 0.982 | 11.051 |
| Quasi-model 9 | 0.668 | 34.189 | 0.982 | 11.879 |
| Quasi-model 10 | 0.652 | 34.096 | 0.982 | 10.755 |
| Quasi-model 11 | 0.627 | 37.331 | 0.981 | 13.242 |
| Quasi-model 12 | 0.675 | 32.031 | 0.984 | 13.457 |
| Quasi-model 13 | 0.653 | 32.323 | 0.982 | 16.079 |
| Quasi-model 14 | 0.587 | 40.240 | 0.979 | 17.839 |
| Quasi-model 15 | 0.652 | 34.940 | 0.983 | 11.406 |
| Quasi-model 16 | 0.581 | 38.914 | 0.979 | 17.276 |
| Quasi-model 17 | 0.639 | 35.714 | 0.981 | 11.327 |
| Quasi-model 18 | 0.631 | 37.362 | 0.982 | 21.058 |
| Quasi-model 19 | 0.633 | 36.307 | 0.981 | 11.496 |
| Quasi-model 20 | 0.648 | 35.428 | 0.983 | 10.846 |
| Quasi-model 21 | 0.641 | 35.073 | 0.982 | 11.048 |
| Quasi-model 22 | 0.603 | 37.123 | 0.980 | 13.544 |
| Quasi-model 23 | 0.599 | 36.916 | 0.980 | 14.809 |
| Quasi-model 24 | 0.645 | 36.012 | 0.982 | 13.768 |
| Quasi-model 25 | 0.644 | 34.716 | 0.982 | 10.852 |
| Quasi-model 26 | 0.587 | 39.618 | 0.980 | 14.791 |
| Quasi-model 27 | 0.637 | 36.732 | 0.981 | 12.821 |
| Quasi-model 28 | 0.604 | 37.401 | 0.981 | 12.017 |
| Quasi-model 29 | 0.633 | 37.365 | 0.982 | 17.743 |
| Quasi-model 30 | 0.613 | 36.678 | 0.980 | 13.557 |

*Model #2: Two compartments, no covariate*

| Model | K | KCP | KPC | V | $R^2$ | MSE |
|---|---|---|---|---|---|---|
| Pop-PK, NPAG | 1.276 | 1.065 | 1.164 | 14.150 | 0.996 | 5.119 |
| Pop-PK, MAP Bayesian | 1.280 | 1.075 | 1.150 | 14.170 | 0.992 | 5.127 |
| Quasi-model 1 | 0.922 | 1.480 | 2.899 | 25.251 | 0.984 | 28.250 |
| Quasi-model 2 | 1.088 | 1.465 | 1.579 | 19.831 | 0.985 | 37.796 |
| Quasi-model 3 | 0.960 | 1.006 | 1.540 | 23.310 | 0.988 | 29.109 |

| | | | | | |
|---|---|---|---|---|---|
| Quasi-model 4 | 1.185 | 1.734 | 2.353 | 19.390 | 0.991 | 14.472 |
| Quasi-model 5 | 0.860 | 1.553 | 2.060 | 29.240 | 0.985 | 30.400 |
| Quasi-model 6 | 1.083 | 2.836 | 2.841 | 18.286 | 0.983 | 28.561 |
| Quasi-model 7 | 0.837 | 0.509 | 1.206 | 25.907 | 0.990 | 20.565 |
| Quasi-model 8 | 1.475 | 2.276 | 1.777 | 13.534 | 0.985 | 21.145 |
| Quasi-model 9 | 1.254 | 2.915 | 2.762 | 20.810 | 0.985 | 21.821 |
| Quasi-model 10 | 1.207 | 1.968 | 1.725 | 17.360 | 0.992 | 16.738 |
| Quasi-model 11 | 0.997 | 0.839 | 0.794 | 16.882 | 0.992 | 6.882 |
| Quasi-model 12 | 0.997 | 1.838 | 2.302 | 23.721 | 0.986 | 32.366 |
| Quasi-model 13 | 1.086 | 1.515 | 2.628 | 21.830 | 0.985 | 43.573 |
| Quasi-model 14 | 0.926 | 0.967 | 1.073 | 20.652 | 0.992 | 22.490 |
| Quasi-model 15 | 1.254 | 0.981 | 1.277 | 14.257 | 0.992 | 7.032 |
| Quasi-model 16 | 1.442 | 1.482 | 3.085 | 21.006 | 0.985 | 17.549 |
| Quasi-model 17 | 1.086 | 1.472 | 1.589 | 19.881 | 0.986 | 57.780 |
| Quasi-model 18 | 0.922 | 2.447 | 4.012 | 23.225 | 0.982 | 25.618 |
| Quasi-model 19 | 1.183 | 1.728 | 2.370 | 19.379 | 0.991 | 16.029 |
| Quasi-model 20 | 0.788 | 0.998 | 2.055 | 29.463 | 0.985 | 26.904 |
| Quasi-model 21 | 1.078 | 2.843 | 3.069 | 20.066 | 0.982 | 29.050 |
| Quasi-model 22 | 0.837 | 0.502 | 1.219 | 25.917 | 0.989 | 22.148 |
| Quasi-model 23 | 1.475 | 2.276 | 1.770 | 13.556 | 0.985 | 28.514 |
| Quasi-model 24 | 1.130 | 2.187 | 2.720 | 18.367 | 0.986 | 15.305 |
| Quasi-model 25 | 1.207 | 1.816 | 1.715 | 1.207 | 0.992 | 18.943 |
| Quasi-model 26 | 1.146 | 2.300 | 2.913 | 17.087 | 0.984 | 13.165 |
| Quasi-model 27 | 0.910 | 1.012 | 2.330 | 22.130 | 0.985 | 13.181 |
| Quasi-model 28 | 1.112 | 2.439 | 3.563 | 22.838 | 0.987 | 34.958 |
| Quasi-model 29 | 1.163 | 2.996 | 2.543 | 17.569 | 0.983 | 21.672 |
| Quasi-model 30 | 1.360 | 2.369 | 2.036 | 16.591 | 0.990 | 13.906 |

*Model #3: One compartment, covariate: creatinine clearance*

| **Model** | **KS** | **KI** | **$V_c$** | **$R^2$** | **MSE** |
|---|---|---|---|---|---|
| Pop-PK, NPAG | 0.004 | 0.095 | 36.775 | 0.990 | 12.167 |
| Pop-PK, MAP Bayesian | 0.004 | 0.096 | 36.623 | 0.981 | 12.193 |
| Quasi-model 1 | 0.004 | 0.061 | 36.875 | 0.980 | 12.984 |
| Quasi-model 2 | 0.004 | 0.072 | 38.016 | 0.980 | 13.424 |
| Quasi-model 3 | 0.004 | 0.047 | 35.408 | 0.982 | 11.319 |
| Quasi-model 4 | 0.004 | 0.035 | 35.817 | 0.983 | 11.974 |
| Quasi-model 5 | 0.004 | 0.029 | 39.303 | 0.981 | 13.007 |
| Quasi-model 6 | 0.004 | 0.045 | 37.772 | 0.981 | 12.051 |
| Quasi-model 7 | 0.004 | 0.060 | 34.553 | 0.982 | 11.502 |
| Quasi-model 8 | 0.004 | 0.085 | 35.762 | 0.981 | 12.646 |
| Quasi-model 9 | 0.004 | 0.083 | 36.332 | 0.981 | 11.991 |
| Quasi-model 10 | 0.004 | 0.059 | 38.352 | 0.981 | 14.999 |
| Quasi-model 11 | 0.004 | 0.039 | 34.239 | 0.982 | 13.480 |
| Quasi-model 12 | 0.004 | 0.018 | 39.121 | 0.979 | 16.365 |
| Quasi-model 13 | 0.004 | 0.051 | 36.548 | 0.982 | 11.400 |
| Quasi-model 14 | 0.004 | 0.060 | 38.717 | 0.980 | 17.248 |

| | | | | | |
|---|---|---|---|---|---|
| Quasi-model 15 | 0.004 | 0.048 | 36.366 | 0.980 | 13.665 |
| Quasi-model 16 | 0.004 | 0.023 | 36.200 | 0.980 | 13.499 |
| Quasi-model 17 | 0.004 | 0.060 | 38.022 | 0.981 | 11.917 |
| Quasi-model 18 | 0.004 | 0.073 | 35.671 | 0.982 | 11.232 |
| Quasi-model 19 | 0.004 | 0.055 | 34.501 | 0.982 | 11.242 |
| Quasi-model 20 | 0.004 | 0.050 | 36.135 | 0.981 | 11.743 |
| Quasi-model 21 | 0.004 | 0.041 | 37.695 | 0.981 | 12.304 |
| Quasi-model 22 | 0.004 | 0.053 | 35.672 | 0.982 | 11.336 |
| Quasi-model 23 | 0.005 | 0.011 | 36.488 | 0.981 | 14.846 |
| Quasi-model 24 | 0.004 | 0.016 | 38.272 | 0.981 | 12.133 |
| Quasi-model 25 | 0.004 | 0.081 | 33.770 | 0.983 | 10.732 |
| Quasi-model 26 | 0.004 | 0.095 | 37.058 | 0.981 | 11.595 |
| Quasi-model 27 | 0.004 | 0.033 | 37.743 | 0.981 | 16.902 |
| Quasi-model 28 | 0.004 | 0.076 | 39.605 | 0.980 | 16.634 |
| Quasi-model 29 | 0.004 | 0.036 | 36.738 | 0.981 | 12.287 |
| Quasi-model 30 | 0.004 | 0.042 | 36.378 | 0.981 | 12.499 |

*Model #4: Two compartments, covariate: creatinine clearance*

| **Model** | **KS** | **KCP** | **KPC** | **KI** | **$V_c$** | **$R^2$** | **MSE** |
|---|---|---|---|---|---|---|---|
| Pop-PK, NPAG | 0.008 | 0.983 | 1.099 | 0.128 | 15.100 | 0.996 | 4.900 |
| Pop-PK, MAP Bayesian | 0.008 | 0.959 | 1.118 | 0.128 | 15.062 | 0.992 | 4.905 |
| Quasi-model 1 | 0.005 | 1.528 | 2.096 | 0.286 | 21.534 | 0.986 | 57.322 |
| Quasi-model 2 | 0.007 | 1.623 | 2.471 | 0.089 | 17.712 | 0.986 | 12.424 |
| Quasi-model 3 | 0.008 | 2.314 | 3.072 | 0.145 | 18.693 | 0.988 | 19.290 |
| Quasi-model 4 | 0.007 | 0.514 | 0.858 | 0.030 | 19.653 | 0.993 | 5.302 |
| Quasi-model 5 | 0.008 | 1.824 | 1.438 | 0.264 | 13.673 | 0.990 | 11.158 |
| Quasi-model 6 | 0.006 | 2.376 | 3.074 | 0.303 | 20.720 | 0.984 | 19.492 |
| Quasi-model 7 | 0.009 | 2.852 | 2.827 | 0.018 | 17.697 | 0.985 | 24.487 |
| Quasi-model 8 | 0.007 | 0.782 | 1.120 | 0.291 | 12.602 | 0.992 | 56.124 |
| Quasi-model 9 | 0.007 | 2.929 | 4.347 | 0.203 | 16.343 | 0.984 | 13.000 |
| Quasi-model 10 | 0.007 | 2.926 | 1.672 | 0.265 | 17.760 | 0.988 | 25.874 |
| Quasi-model 11 | 0.009 | 1.792 | 1.466 | 0.291 | 10.942 | 0.990 | 10.156 |
| Quasi-model 12 | 0.008 | 3.702 | 3.598 | 0.166 | 16.816 | 0.984 | 45.995 |
| Quasi-model 13 | 0.005 | 0.998 | 1.961 | 0.217 | 22.645 | 0.987 | 24.551 |
| Quasi-model 14 | 0.008 | 4.128 | 3.215 | 0.123 | 14.196 | 0.984 | 14.264 |
| Quasi-model 15 | 0.008 | 3.993 | 3.766 | 0.098 | 18.565 | 0.986 | 18.156 |
| Quasi-model 16 | 0.008 | 2.389 | 2.608 | 0.156 | 18.938 | 0.989 | 9.869 |
| Quasi-model 17 | 0.007 | 1.005 | 1.120 | 0.044 | 19.570 | 0.991 | 29.703 |
| Quasi-model 18 | 0.006 | 1.877 | 2.765 | 0.232 | 21.686 | 0.986 | 25.206 |
| Quasi-model 19 | 0.009 | 3.414 | 2.687 | 0.209 | 15.029 | 0.986 | 43.277 |
| Quasi-model 20 | 0.005 | 2.627 | 4.169 | 0.300 | 21.240 | 0.982 | 16.273 |
| Quasi-model 21 | 0.007 | 2.353 | 2.790 | 0.122 | 17.154 | 0.985 | 14.533 |
| Quasi-model 22 | 0.009 | 3.500 | 3.107 | 0.191 | 14.462 | 0.985 | 33.624 |
| Quasi-model 23 | 0.010 | 2.293 | 1.673 | 0.169 | 12.638 | 0.987 | 29.671 |
| Quasi-model 24 | 0.007 | 2.508 | 2.412 | 0.220 | 20.311 | 0.987 | 30.340 |
| Quasi-model 25 | 0.008 | 1.788 | 2.231 | 0.004 | 18.217 | 0.985 | 13.796 |

| | | | | | | | |
|---|---|---|---|---|---|---|---|
| Quasi-model 26 | 0.005 | 1.169 | 2.238 | 0.313 | 21.319 | 0.986 | 11.211 |
| Quasi-model 27 | 0.005 | 0.652 | 1.061 | 0.231 | 23.553 | 0.990 | 30.115 |
| Quasi-model 28 | 0.007 | 2.753 | 3.219 | 0.132 | 20.645 | 0.984 | 11.313 |
| Quasi-model 29 | 0.012 | 1.206 | 1.070 | 0.004 | 8.239 | 0.991 | 80.837 |
| Quasi-model 30 | 0.009 | 2.969 | 3.061 | 0.067 | 16.684 | 0.985 | 36.019 |
